# Syndecan-4 interacts directly with β-parvin and regulates the ILK-PINCH-β-parvin complex, the β-parvin-β-PIX-Rac1 axis, and cardiomyocyte geometry in a sex-dependent manner

**DOI:** 10.3389/fcell.2025.1569185

**Published:** 2025-08-29

**Authors:** Sabrina Bech Mathiesen, Thea Parsberg Støle, Andreas Romaine, Marianne Lunde, Marita Martinsen, Jan Magnus Aronsen, Ivar Sjaastad, William E. Louch, Geir Christensen, Cathrine Rein Carlson

**Affiliations:** ^1^ Institute for Experimental Medical Research, Oslo University Hospital and University of Oslo, Oslo, Norway; ^2^ Department of Molecular Medicine, Institute of Basic Medical Sciences, University of Oslo, Oslo, Norway; ^3^ Department of Pharmacology, Oslo University Hospital, Oslo, Norway

**Keywords:** syndecan-4 (Sdc4), β-parvin, Rac1, IPP complex, β-Pix, cardiomyocyte length, heart, sex-specific differences

## Abstract

Syndecan-4 is a ubiquitously expressed transmembrane proteoglycan that links the extracellular matrix to intracellular protein networks. It is located at stress-sensing structures in cardiomyocytes, including costameres and Z-discs, and in male mice, it is involved in the hypertrophic response to cardiac pressure overload. We have recently found female syndecan-4 KO cardiomyocytes, without challenge, to be smaller in area. Smaller cardiomyocytes with elongation defects have been observed in animal models with β-parvin deficiency, where the loss of this mechano-sensor disrupts the guanine nucleotide exchange factor (GEF) β-PIX-GTPase Rac1 axis, which is essential for proper cell elongation. β-parvin, together with integrin-linked kinase (ILK) and particularly interesting new cysteine-histidine-rich protein (PINCH), constitutes the IPP complex (ILK-PINCH-parvin), which is part of the integrin consensus adhesome. Interestingly, in a previous large cardiac interactome study, we have identified β-parvin, as well as ILK, β-PIX, and Rac1 as potential syndecan-4 partners. To better understand the syndecan-4-β-parvin association, we mapped their interaction and investigated the effect of syndecan-4 ablation on the IPP complex, the β-parvin-β-PIX-Rac1 axis, and cardiomyocyte geometry in both females and males. Interestingly, genetic ablation of syndecan-4 resulted in shorter cardiomyocytes in females only. The syndecan-4-β-parvin interaction was mapped to accessible sequences within the N-terminal, linker, and CH2 domains of β-parvin and the unique variable C2 cytoplasmic region of syndecan-4. Syndecan-4 ablation resulted in lower levels of membrane-localized β-parvin in both sexes and sex-specific differences in its associated partners ILK and PINCH, suggesting that syndecan-4 is linked to integrin signaling through the IPP complex. Finally, Rac1, known for its involvement in cell size regulation, and some of its regulators, β-PIX, RhoGDIα, and the serine/threonine kinase PAK, showed sex-specific alterations following syndecan-4 ablation. Altogether, our data suggest that syndecan-4 binds directly to β-parvin and regulates cardiomyocyte length, the IPP complex, and the β-parvin-β-PIX-Rac1 in a sex-dependent manner. These findings highlight a sex-specific role for syndecan-4 in cardiomyocyte structure, offering new insight into the molecular basis for sex differences in cardiac biology.

## 1 Introduction

Syndecan-4 is ubiquitously expressed and part of the syndecan family of four transmembrane proteoglycans (Elfenbein and Simons, 2013). These proteins extend large heparan sulfate chains into the extracellular matrix (ECM) from their N-terminal core domain but also span the plasma membrane, placing a short cytoplasmic tail in the cytosol (Elfenbein and Simons, 2013). The tail can be divided into three regions: the membrane-proximal region, C1, the middle variable region, V, and the membrane-distal region, C2 (Couchman et al., 2015). C1 and C2 are shared amongst the four family members, but the V-region is unique to each of the syndecans (Couchman et al., 2015). This transmembrane composition allows syndecan-4 to engage via its N-terminus to ECM components, both soluble and insoluble, and at the same time connect to intracellular proteins, including the cytoskeleton, through its C-terminus. This arrangement makes syndecan-4 an ideal sensor and transducer of changes in the ECM (Roper et al., 2012).

In cardiomyocytes, syndecan-4 localizes to stress-sensing structures, including costameres and Z-discs (VanWinkle et al., 2002), which link the ECM to the cytoskeleton (Sarantitis et al., 2012). Consistent with a role for syndecan-4 as a stress sensor and transducer, the most profound effects of syndecan-4 ablation are observed when the syndecan-4 knockout (KO) mice are challenged. Indeed, syndecan-4 KO mice do not develop the concentric hypertrophy that normally occurs upon pressure overload (Finsen et al., 2011), but develop more myocardial damage and larger infarcts after ischemia-reperfusion (Echtermeyer et al., 2011). Following myocardial infarction, syndecan-4 KO mice exhibit greater impairment of cardiac function and increased mortality due to cardiac rupture (Matsui et al., 2011). In the absence of such challenges, the syndecan-4 KO heart appears morphologically and functionally normal without any developmental defects (Finsen et al., 2011; Matsui et al., 2011). However, most of the research investigating the role of syndecan-4 and the effects of its ablation in cardiovascular disease has, by us and others, been performed in male mice.

We have recently found that female but not male syndecan-4 KO hearts exhibit smaller cardiomyocytes (Støle et al., 2022), suggesting a sex-dependent role for syndecan-4 in regulating cell size. Interestingly, smaller cardiomyocytes have been reported upon loss of the mechano-sensor β-parvin, although no sex differences were reported in this study (Thievessen et al., 2022). β-parvin has been proposed to drive cell elongation and serial sarcomere assembly through its interaction with β-PIX. This interaction, in turn, leads to the activation of the small GTPase Rac1, and thus regulation of cardiomyocyte size and shape (Thievessen et al., 2022). The parvin family consists of three parvin isoforms (α, β and γ), where α-parvin is ubiquitously expressed, β-parvin is mainly expressed in the heart and other muscles, and γ-parvin is restricted to the hematopoietic system (Korenbaum et al., 2001; Yamaji et al., 2001; Chu et al., 2006). β-parvin together with integrin-linked kinase (ILK) and particularly interesting new cysteine-histidine-rich protein (PINCH) constitute the IPP complex (ILK-PINCH-parvin). The IPP complex is part of the integrin consensus adhesome (Horton et al., 2016), where integrin binding to ILK (Hannigan et al., 1996; Pasquet et al., 2002) is linked with the actin cytoskeleton through PINCH and parvin (Vaynberg et al., 2018).

Interestingly, we have previously identified β-parvin as a potential syndecan-4 partner in a large interactome study, using two different affinity-purification techniques coupled to mass-spectrometry (AP-MS) approaches (Mathiesen et al., 2019). ILK, β-PIX (ARHGEF7) and Rac1 were also identified to co-precipitate with cardiac syndecan-4 (Mathiesen et al., 2019). Additionally, Rac1 activity has been found to associate with syndecan-4 in mouse embryonic fibroblasts (Bass et al., 2007).

In the present study, we have investigated the syndecan-4-β-parvin interaction in greater detail and studied the effect of syndecan-4 ablation on cardiomyocyte geometry, the IPP-complex, and the β-parvin-β-PIX-Rac1 axis, in females and males. Our data suggest that syndecan-4 binds directly to β-parvin and regulates cardiomyocyte geometry, the IPP complex, and the β-parvin-β-PIX-Rac1 axis in a sex-dependent manner.

## 2 Experimental procedures

### 2.1 Animal work

All animal work was pre-approved by the Norwegian Animal Research Committee (FOTS IDs: 29268, 7695, and 15445 for mice, and 30114 for rats) and conformed to the Guide for the Care and Use of Laboratory Animals (NIH publication 85–23, revised 2011, US). For subcellular fractions and lysates of mouse left ventricles (LVs), we employed 12–15-week-old female and male syndecan-4^−/−^ (KO) and syndecan-4^+/+^ (WT) mice with a C57BL/6J genetic background (Jackson Laboratory, Bar Harbor, Maine, United States), bred in-house. Heart weight (Støle et al., 2022) and body weight (Supplementary Table S1) did not differ between genotypes for respective sexes. For rat subcellular fractions and lysates, LVs from 2–4-month-old male Wistar rats (Janvier Labs, Le Genest-Saint-Isle, France) were used. All animals were euthanized by cardiac excision during deep surgical anesthesia induced by inhalation of a mixture of 5% isoflurane and 95% O_2_, where pedal withdrawal reflexes ceased in response to pinching with tweezers.

### 2.2 Rat and mouse cardiomyocyte isolation

Hearts were excised from adult male rats or male and female syndecan-4 KO and WT mice under deep surgical anesthesia as described above. For each heart, the aorta was subsequently cannulated on a constant flow Langendorff perfusion system coupled to a perfusion pump. Hearts were flushed with approximately 5–10 mL of isolation buffer (120 mM NaCl, 5.4 mM KCl, 0.5 mM MgCl_2_, 0.4 mM NaH_2_PO_4_, 25 mM HEPES and 5.5 mM D-glucose, pH 7.4) to clear the blood, at a flow rate of 2 mL/min. Isolation buffer containing 2 mg/mL collagenase type II (Worthington Biochemical Corporation, Lakewood, NJ, United States) was perfused through the heart for 7–15 min at 37°C. Following digestion, the LV was dissected from the right ventricle and minced into chunks in isolation buffer containing 1 mg/mL BSA. Chunks were then transferred to a falcon tube containing 0.2 mg DNase (LS002006, Worthington Biochemical Corporation), collagenase, and 250 µL BSA. Isolated cells were then filtered through a 200 µm filter and left to sediment before the Ca^2+^ concentration was gradually increased to 0.2 mM. Cardiomyocytes were used within 2 h of isolation.

### 2.3 Subcellular fractionation

Syndecan-4 KO and WT LVs, and isolated male adult primary rat cardiomyocytes were subjected to subcellular fractionation according to the manufacturer’s protocol (#2145, Sigma Merck, Darmstadt, Germany). The subcellular enriched fractions analyzed in Figure 2B, and Supplementary Figure S2C–E are from the same samples used in Støle et al., 2024.

### 2.4 Cardiomyocyte length and width measurements

Freshly isolated cardiomyocytes from the LV of female and male syndecan-4 KO and WT mice were fixed in 4% PFA, quenched in 150 mM glycine and thereafter permeabilized with 0.5% Triton X-100 (10 min at each step, at room temperature). Cardiomyocytes were plated on laminin (#L2020, mouse, Sigma Merck, Darmstadt, Germany) coated glass bottom dishes (No 1.5, Ø 14 mm, γ-irradiated, Martek Corporation, Columbia, MD, United States) and left to adhere for 1 hour. For visualization of the sarcolemma and nuclei, cells were stained with wheat germ agglutinin (WGA) 488 (1:400, #W11261, Thermo Fisher Scientific, Waltham, MA, United States) and DAPI 405 (0.1 mg/mL, #MBD0015, Sigma Merck), respectively. Imaging was performed on a ZEISS LSM 800 (Carl Zeiss AG, Oberkochen, Germany) confocal microscope with Airyscan mode, using a plan-apo 63X 1.4 NA oil objective. Cross-sectional images were obtained at the axial midpoint of the cell. Cardiomyocyte length was measured at the cell’s longest point, outlined by sarcolemma WGA staining. Width was defined at the midpoint of the cell’s length, following the direction of the Z-discs. Length and width measurements were manually performed on the same cells for which we have previously measured cross-sectional area (Støle et al., 2022). Cardiomyocytes with insufficient WGA staining detectable for manual selection of length and width measurements were excluded from the analysis. Measurements were performed in FIJI 1.52p (NIH).

### 2.5 Antibodies

Anti-SDC4 (2 µg per IP, KY/8.2, #550350, BD Pharmingen™, Franklin Lakes, NJ, United States), anti-rat IgG (2 µg per IP, sc-2026, Santa Cruz Biotechnology, Dallas, TX, United States), anti-SDC4_extr_ (1:1000, 1 x casein, order number #515448–1/-2 custom made by GenScript, Piscataway, NJ, United States), anti-SDC4 (1:1000, 1 x casein, order number #429716, custom made by GenScript), anti-biotin-HRP (1:5000, 1 x TBST, A-0185, Sigma Merck, Darmstadt, Germany), anti-FLAG (1:2000, 1 x casein, F1804, Sigma Merck), anti-β-parvin (1:500, 1 x casein, D-2, sc-374581, Santa Cruz Biotechnology), anti-ILK (1:1000, 1 x casein, #3862, Cell Signaling Technology, Danvers, MA, Unites States), anti-PINCH (1:1000, 1 x casein, #612710, BD Pharmingen), anti-Rac1 (1:1000, 1 x casein, #610651, BD Pharmingen), anti-β-PIX (1:1000, 1 x casein, #07–1450-I, Sigma Merck), anti-RhoGDIα (1:500, 1 x casein, sc-373725, Santa Cruz Biotechnology), anti-pSer199/294, pSer192/197-PAK1/2 (1:1000, 5% BSA, #2605, Cell Signaling), anti-PAK1 (1:1000, 5% BSA, #2602, Cell Signaling), anti-β1-integrin (1:1000, 1 x casein, AF2406, R&D systems), anti-GAPDH (1:500, 1 x casein, V-18, sc-20357, Santa Cruz Biotechnology), anti-GAPDH (1:500, 1 x casein, 0411, sc-47724, Santa Cruz Biotechnology), anti-NCX1 (1:1000, 5% milk, #3302, GenScript), anti-α-actinin (1:1000, 1 x casein, ab68167, abcam, Cambridge, United Kingdom), anti-Histone H3 (1:2000, 1 x casein, #4499, Cell Signaling), anti-mouse IgG horse radish peroxidase (HRP) (1:3000, 1 x TBST, NA931V, GE Healthcare, Marlborough, MA, United States), anti-rabbit IgG HRP (1:3000, 1 x TBST, NA934V, GE Healthcare), and anti-rat IgG HRP (1:3000, 1 x TBST, NA935, GE Healthcare).

### 2.6 Peptides and recombinant proteins

Peptides were made to >80% purity by GenScript (GenScript, Piscataway, NJ, United States). Peptides mimicking the cytosolic component of syndecan-4 and its scrambled control incorporated an ahx linker to prevent possible steric hindrance:

Biotin-ahx-SDC4_cyt_: RMKKKDEGSYDLGKKPIYKKAPTNEFYA (identical in human, rat and mouse), and

Biotin-ahx-SDC4_scram_: GTKYPKMDRGKLFKYKAKPEDNESAYIK

Full-length (FL) recombinant human C-Myc/DDK-β-parvin was obtained from OriGene (TP308490, Rockville, MD, United States). Recombinant rat 8xHIS-β-parvin (85% purity), rat 8xHIS-α-parvin (85% purity), and mouse 8xHIS-ILK (S343D) (75% purity) were custom-made by GenScript. Although ILK is likely a pseudokinase (Fukuda et al., 2009; Lange et al., 2009), one study suggests the S343D mutation generates a constitutively active kinase (Persad et al., 2001).

### 2.7 Cell culture and transient transfection

Human embryonic kidney 293 (HEK293) cells (ATCC CRL-1573™, ATCC, Manassas, VA, United States) were used due to ease of transfection. For culturing, Dulbecco’s modified Eagles medium (DMEM) (41965039, Gibco, Thermo Fisher Scientific, Waltham, MA, United States) with added 10% fetal bovine serum (FBS) and 1% penicillin/streptomycin (PS) (P0781, Sigma Merck, Darmstadt, Germany) was used, and cells were kept in a humidified incubator at 37°C in 5% CO_2_. For transient transfection, cells were kept without PS for 24 h and then transfected via the CaCl_2_ method as described previously (Jordan et al., 1996; Mathiesen et al., 2019). A total of 8 µg plasmid DNA was used per transfection. Lysates were prepared 24 h after transfection with ice-cold IP buffer (150 mM NaCl, 20 mM HEPES (pH 7.5), 1 mM EDTA, 1% Triton X-100 with cOmplete protease inhibitor cocktail (5056489001, Sigma Merck, Darmstadt, Germany)) and samples were transferred to ice immediately after lysis. Cloning was performed by GenScript in pcDNA3.1 or pCEP4 vectors. DNA constructs were:

3xFLAG-β-parvin FL (amino acids (aa) 1–365),

3xFLAG-β-parvin N-terminal (aa 1–87),

3xFLAG-β-parvin CH1 (aa 72–201),

3xFLAG-β-parvin linker (aa 193–261),

3xFLAG-β-parvin CH2 (aa 252–365),

3xFLAG-β-parvin Δlinker (aa 196–254 deleted),

3xFLAG-β-parvin ΔCH2 (aa 255–362 deleted),

3xFLAG-β-parvin Δlinker+ΔCH2 (aa 196–362 deleted),

SDC4 FL (aa 1–198), and

SDC4 Δcyt (aa 171–198 deleted).

All β-parvin constructs were built using rat sequences, while syndecan-4 constructs followed mouse sequences.

### 2.8 LV lysates

LV tissue was homogenized with TissueLyser (#85300, Qiagen Nordic) in ice-cold lysis buffer (20 mM Hepes (pH7.5), 150 mM NaCl, 1 mM EDTA and 0.5% Triton-X100). Complete EDTA-free protease inhibitor cocktail (#05056489001, Roche Applied Science, Merck, Darmstadt, Germany) and PhosSTOP (#04906837001, Roche Applied Science) were added to the lysis buffer. The homogenates were centrifuged at 14,000 rcf for 10 min at 4°C, and supernatants were stored at −80°C. Protein concentrations were determined by Micro BCA protein assay kit (#23235, Thermo Fisher Scientific, Waltham, MA, United States).

### 2.9 Immunoprecipitation

For immunoprecipitation (IP) experiments, 200 µL of HEK293, LV, or cardiomyocyte lysate was mixed with 2 µg of the given antibody and 50 µL protein A/G agarose beads (sc-2003, Santa Cruz Biotechnology, Dallas, TX, United States) and incubated overnight (O/N) at 4°C with rotation. Protein complexes were collected by three times washing in IP buffer and centrifugation at 4°C. The final pellet was boiled in 60 µL 2 x loading buffer (0.75 M sucrose, 3.75% SDS, 31.25 mM Tris-HCl (pH 6.8), 0.1 mM EDTA (pH 7.5), 100 mM DTT, 0.005% bromophenol blue) at 96°C and analyzed by immunoblotting.

### 2.10 Pull-down

For each pull-down, 75 µL monoclonal anti-biotin agarose beads (#A1559, Sigma Merck, Darmstadt, Germany) diluted 1:1 in phosphate-buffered saline (PBS) was mixed with 10 µL biotinylated peptide (1 mM) and 75 µL PBS under gentle rotation for 2 h at 4°C. Beads were collected by centrifugation (4°C) and washed once in PBS. 100 μL HEK cell lysate or 1 µg recombinant protein dissolved in 1 x PBS was added to the beads and the mixture was further incubated under rotation for 2 h at 4°C. Protein complexes were collected after three washes in IP buffer, by adding 2 x loading buffer and boiling the pellet for 5 min at 96°C. Pull-down of active GTP-bound Rac1 was performed with a commercially available kit (PAK02, Cytoskeleton, Denver, CO, United States) according to the manufacturer’s instructions, with 400–600 µg LV lysate as input. Pull-downs were visualized by immunoblot analysis.

### 2.11 Western blotting

Samples were loaded at equal protein concentrations and run on 18-well 4%–15% Criterion™ Tris-HCl precast gels (#3450028, Bio-Rad, Hercules, CA, United States) or 4%–15% Criterion™ TGX precast gels (#5671084, Bio-Rad). Proteins were blotted onto a PVDF membrane (#1704157, Bio-Rad, or #0301004001, Sigma Merck, Darmstadt, Germany) via the semi-dry TransBlot^®^ Turbo Transfer System (#1704150, Bio-Rad). The membranes were blocked in either 5% milk, 5% BSA, or 1 x casein in TBST (Tris-buffered saline with 1% Tween-20 (#1610781, Bio-Rad)) for 1 h before being incubated with the primary antibody O/N at 4°C. After three times 5 min washes in TBST and 1 h incubation with HRP-conjugated secondary antibodies followed by an additional three TBST washes, the membranes were developed in ECL Prime (RPN 2232, GE Healthcare, Marlborough, MA, United States). Reprobing was done by stripping with Restore™ Western blot Stripping Buffer (#21059, Thermo Fisher Scientific, Waltham, MA, United States). Equal protein loading was verified by Revert 700 protein staining (Licor) (#926–11021, LI-COR Biosciences, Lincoln, NE, United States) or ProBlue SafeStain (Coomassie) (#G00PB001, Giotto Biotech, FI, Italy).

### 2.12 ELISA

For ELISA, the wells were coated with recombinant β-parvin and performed as previously described in Mathiesen et al., 2019.

### 2.13 Peptide overlay

The cytoplasmic tail of syndecan-4 (mouse) or FL β-parvin (rat) protein was synthesized as 20′mer overlapping peptides with 3 amino acids offset onto a cellulose membrane by a Multipep automated peptide synthesizer (CEM Corporation, Matthews, NC, United States) (Frank and Overwin, 1996). After membrane blocking, antibodies, or a solution with recombinant protein or biotin-ahx-SDC4 were added and incubated O/N at 4°C. Membranes were developed as described for immunoblotting.

### 2.14 Biacore/surface plasmon resonance (SPR) analysis

SPR analysis was performed using Biacore X100. Biotinylated syndecan-4 cytoplasmic tail (biotin-ahx-SDC4_cyt_) (ligand) was immobilized on SA chips (BR10032, Cytiva, Marlborough, MA, United States) at 100–200 resonance units (RU) using SW-buffer (2 M NaCl, 100 mM NaOH) diluted in isopropanol (1:1). Recombinant β-parvin or α-parvin (analyte) were diluted over a range of concentrations (26.5–277.7 nM) in 1x RB buffer (0.01 Hepes, pH 7.4, 150 mM NaCl, 3 mM EDTA, 0.005% v/v Surfactant P20) (BR100826, Cytiva) and injected over the sensor surface at a flow rate of 30 μL/min for 180 s. Dissociation time was 600 s at the same flow rate. Obtained sensorgrams were analyzed by Biacore X100 evaluation software, using the 1:1 Langmuir interaction model (described in detail (Shi et al., 2025)).

### 2.15 Real-time PCR

RNA extraction and real-time PCR of female and male syndecan-4^+/+^ LVs (WT) was performed as previously described (Støle et al., 2022) where the relative gene expression was calculated from a standard curve and normalized to the housekeeping gene 60S ribosomal protein L32 (RPL32).

### 2.16 Statistics

Data are presented as mean ± SEM. The normality distribution of each dataset was analyzed with Shapiro-Wilk tests. Normally distributed data were tested with unpaired two-tailed *t*-tests, and non-normally distributed data with two-tailed Mann-Whitney *U*-tests or Kruskal-Wallis tests (GraphPad Prism 9.4.1, La Jolla, CA, United States). P < 0.05 was accepted as significant. Immunoblot semi-quantification was performed in FIJI 1.52p (NIH). Figure assembly and illustrations were made in Adobe Illustrator CS6 (Adobe Inc., San Jose, CA, United States).

## 3 Results

### 3.1 Syndecan-4 ablation leads to shorter cardiomyocytes in female mice only

**FIGURE 1:**
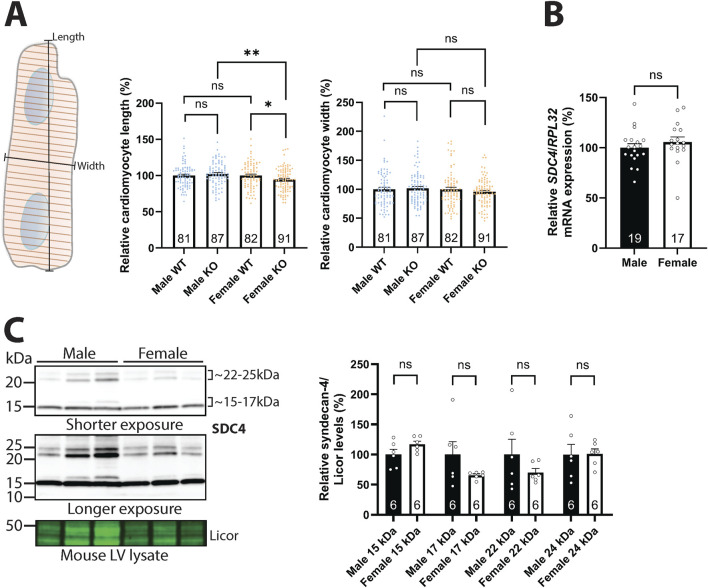
Syndecan-4 ablation leads to shorter cardiomyocytes in female mice only. **(A)** An illustration of the length and width measurements is given in the left panel. Cardiomyocyte length (middle panel) and width (right panel) of male and female syndecan-4 KO and WT mice (n = 81–91 cells from n = 3 mice in each group). Female and male KO cells are related to their respective WT (set to 100%). **(B)** Relative *SDC4* mRNA levels in WT female and male LVs, normalized to *RPL32* (n = 17–19 hearts). **(C)** Immunoblot of syndecan-4 in WT male and female mouse LV lysates (n = 6 hearts). Syndecan-4 positive bands are annotated with the approximate molecular weight on the right with brackets. Licor was used as a protein loading control (20 µg). Specificity of the syndecan-4 bands using antibody-blocking peptides is shown in Supplementary Figure S1B. All values are presented as mean percentages ±SEM and differences between the groups were analyzed by Mann-Whitney *U*-tests **(A)** or unpaired two-tailed *t*-tests **(B,C)** due to non-normal or normal distribution analyzed by Shapiro-Wilk testing (*p < 0.05, **p < 0.01, ns: not significant).

We have previously found that female syndecan-4 KO cardiomyocytes are smaller in area compared to female WT (Støle et al., 2022). Closer inspection revealed that these female syndecan-4 KO cells were on average 6 µm (5.5%) shorter compared to female WT cells, while the thickness was not altered (Figure 1A, left and right panel). In contrast, no significant changes were observed between male syndecan-4 KO and WT cardiomyocytes (Figure 1A), suggesting a sex-dependent role for syndecan-4 in regulating cardiomyocyte geometry. No differences in length or width were observed between female and male WT cardiomyocytes (Figure 1A). We next investigated syndecan-4 gene expression and protein levels in the left ventricles (LVs) of female and male WT mice. Using RT-PCR, we found that syndecan-4 expression was unaltered in females vs. males (Figure 1B). At the protein level multiple bands appeared with immunoblotting (Supplementary Figure S1A), however, only the syndecan-4 core protein at ∼22–25 kDa (Manon-Jensen et al., 2010; Finsen et al., 2011; Mathiesen et al., 2019) and shed syndecan-4 fragment at ∼15–17 kDa (Strand et al., 2013) were confirmed positive with antibody epitope-blocking experiments (Supplementary Figure S1B). Consistent with gene expression data (Figure 1B), no statistically significant differences were detected in the levels of the syndecan-4 core protein and shed fragments in females vs. males (Figure 1C).

### 3.2 Syndecan-4 binds to β-parvin in cardiomyocytes

**FIGURE 2:**
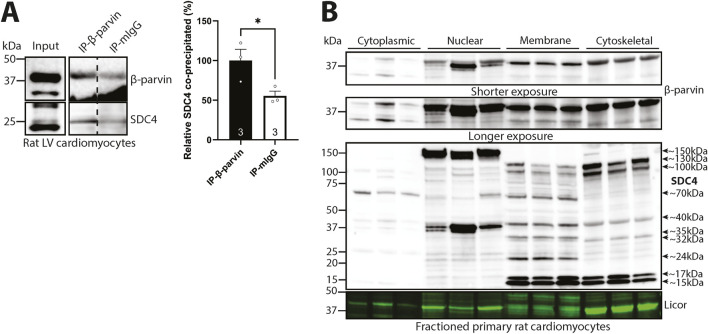
Syndecan-4 binds to β-parvin in cardiomyocytes. **(A)** Immunoprecipitation and immunoblotting using anti-β-parvin (D-2) in adult cardiomyocyte lysates from male rats. Co-precipitation of syndecan-4 was detected with a custom-made antibody against the cytoplasmic domain of syndecan-4. Non-relevant mouse IgG was used as a negative control (n = 3 hearts). Lysate input is shown on the left. Dotted line indicates representative lanes are a montage from the same exposed blot. Values are presented as mean ± SEM where differences between the groups were tested with an unpaired two-tailed *t*-test due to normal distribution analyzed by Shapiro-Wilk testing (*p < 0.05). Specificity of β-parvin bands using antibody-blocking peptides was confirmed in Supplementary Figure S2A. **(B)** Immunoblots of β-parvin and syndecan-4 in subcellular enriched fractions of adult male rat cardiomyocytes. Syndecan-4 positive bands are annotated with the approximate molecular weight on the right with arrows (n = 3 rat hearts). Licor was used to show equal protein loading within fractions (20 µg). Specificity of β-parvin and syndecan-4 bands using antibody-blocking peptides, and subcellular compartment markers are shown in Supplementary Figure S2C–E.

To investigate the mechanism by which syndecan-4 regulates cardiomyocyte length, we first verified that the syndecan-4-β-parvin interaction occurred in cardiomyocytes. Using β-parvin antibodies in male rat cardiomyocyte lysates, we were able to co-precipitate the weak core protein of syndecan-4 (SDC4) at ∼25 kDa (antibody epitope mapped in (Finsen et al., 2011)) (Figure 2A). Notably, the β-parvin antibody recognized two bands of ∼35 and ∼42 kDa in the cardiomyocyte lysate input, but only the 42 kDa band was precipitated (Figure 2A, upper panel). Since we only detected precipitation of the 42 kDa β-parvin variant, it could be hypothesized that the two β-parvin variants may fold differently, although this remains to be shown. The 35 kDa variant may be due to alternative translation initiation, which can result in β-parvin with a theoretical molecular mass of ∼35 kDa (Sepulveda and Wu, 2006). The specificity of the 35 and 42 kDa bands was confirmed in antibody epitope-blocking experiments where both bands disappeared or became weaker, suggesting they were of β-parvin origin (Supplementary Figure S2A, LV lysate from mouse and rat). Moreover, epitope mapping showed that the β-parvin antibody recognized the epitope described by the manufacturer, and importantly, it did not recognize α-parvin (Supplementary Figure S2B).

To further investigate the localization of β-parvin and syndecan-4 in cardiomyocytes, we subcellular fractioned primary male rat cardiomyocytes before immunoblotting. The two β-parvin bands of 35 kDa and 42 kDa were detected in all four subcellular fractions, although at lower levels in the cytoplasm than the nuclear, membrane, and cytoskeletal-enriched fractions (Figure 2B, upper two panels). However, the 35 kDa band was mainly present in the cytoplasmic fraction, whereas the 42 kDa β-parvin band was mainly present in the other fractions (specificity of the two β-parvin positive bands in the subcellular enriched fractions was confirmed in Supplementary Figure S2C). Syndecan-4 was also detected in all four subcellular-enriched fractions, although in different forms, as we have previously shown (Støle et al., 2024) (Figure 2B, lower panel). The core protein of syndecan-4 at ∼22–25 kDa (Manon-Jensen et al., 2010; Finsen et al., 2011; Mathiesen et al., 2019) was mainly detected in the nuclear and membrane-enriched compartments, while the shedded cytoplasmic tail at ∼15–17 kDa was detected in the membrane and cytoskeletal-enriched fractions. Additionally, we also detected large syndecan-4-positive bands at ∼32, ∼35, ∼40, ∼70, ∼100, ∼130 and ∼150 kDa in the various subcellular-enriched fractions, indicated with arrows and approximate molecular weights, which may be modified SDS-resistant homo- or hetero-oligomers of syndecan-4 (Choi et al., 2005). As previously shown in cardiomyocyte subcellular fractions (Støle et al., 2024), all syndecan-4-positive bands, except for the 40 kDa cytoskeleton and 70 kDa membrane bands, disappeared or were weaker with antibody-blocking experiments, suggesting true-positive specificity against syndecan-4 (Supplementary Figure S2D). Enrichment of fractions was confirmed by subcellular compartment markers (Supplementary Figure S2E).

### 3.3 Syndecan-4 binds directly to β-parvin through its cytoplasmic V and C2 domains

**FIGURE 3:**
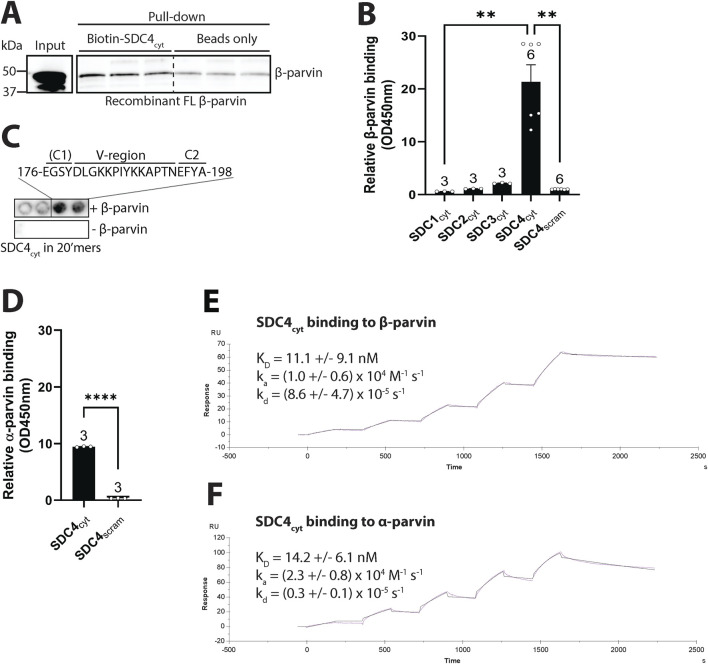
Syndecan-4 binds directly to β-parvin through its cytoplasmic V and C2 domains. **(A)** Biotin-ahx-SDC4_cyt_ was tested for binding against recombinant FL human β-parvin in a pull-down experiment. Precipitated β-parvin was detected by immunoblot analysis (n = 3 replicates). Beads were used as a negative control. Dotted line indicates representative lanes are a montage from the same exposed blot. **(B)** The biotinylated cytoplasmic tail of syndecans 1–4 (SDC1-4_cyt_) were tested for binding against recombinant FL human β-parvin in an ELISA-based assay (n = 3-6 replicates). A biotinylated SDC4_cyt_ scrambled peptide (SDC4_scram_) was used as a negative control. **(C)** 20′mers overlapping peptides of the cytoplasmic domain of syndecan-4 (mouse) overlaid with recombinant FL β-parvin and binding detected with β-parvin antibodies (n = 4 membranes) (upper membrane). Peptide membranes without β-parvin incubation were used as a negative control (lower membrane). The syndecan-4 C1 region is in parentheses because the identified β-parvin binding site only partially includes this sequence. **(D)** SDC4_cyt_ was tested for binding against recombinant FL rat α-parvin in an ELISA assay. SDC4_scram_ was used as a negative control (n = 3 replicates). **(E,F)** SPR analysis was done by immobilizing biotin-ahx-SDC4_cyt_ on an SA chip and measuring the response while injecting a range of concentrations of recombinant **(E)** FL rat β-parvin or **(F)** FL rat α-parvin (n = 4 replicates). Values in B and D are presented as mean ± SEM where differences between the groups were tested with a Kruskal-Wallis test **(B)** or an unpaired two-tailed *t*-test **(D)** due to non-normal or normal distribution analyzed by Shapiro-Wilk testing (**p < 0.01, ****p < 0.0001).

To test for a direct interaction between β-parvin and syndecan-4, we performed a pull-down experiment with a biotinylated peptide covering the cytoplasmic tail of syndecan-4 (SDC4_cyt_) and recombinant full-length (FL) human β-parvin. Immunoblotting with β-parvin antibodies showed that recombinant β-parvin precipitated with syndecan-4 (Figure 3A). To investigate whether the interaction was specific only to syndecan-4 or also to other syndecans, we performed an ELISA-based assay coated with recombinant β-parvin overlayed with the cytoplasmic domains of syndecan-1-4 (SDC1-4_cyt_) or a scrambled control peptide (SDC4_scram_). Only syndecan-4 bound stronger than the negative scrambled control (Figure 3B), suggesting the interaction with β-parvin is specific only to syndecan-4. To map the β-parvin binding in syndecan-4, SDC4_cyt_ was synthesized as 20-mer overlapping peptides with three residues offset on membranes. By overlaying the membrane with recombinant FL β-parvin protein and immunoblotting with β-parvin antibodies, we found that β-parvin bound to the region of SDC4_cyt_ distal to the membrane, which encompassed mainly the V and C2 regions (Figure 3C).

Due to the high homology between β- and α-parvin, and the co-precipitation of α-parvin with cardiac syndecan-4 observed in our previous interactome study (Mathiesen et al., 2019), we also assessed the binding of SDC4_cyt_ to recombinant α-parvin. An ELISA-based assay showed that SDC4_cyt_ also bound recombinant α-parvin (Figure 3D). To assess differences in binding affinity and kinetics between α- and β-parvin to SDC4_cyt_, we performed surface plasmon resonance. A range of concentrations of recombinant α- or β-parvin was injected over immobilized SDC4_cyt_ on SA chips and analyzed with the fit of a 1:1 interaction model (Langmuir). The dissociation equilibrium constant (*K*_D_) for the β-parvin-syndecan-4 interaction was 11.1 ± 9.1 nM with an association rate constant (*k*_a_) = (1.0 ± 0.6) x 10^4^ M^-1^ s^-1^ and a dissociation rate constant (*k*_d_) = (8.6 ± 4.7) x 10^–5^ s^-1^ (Figure 3E). For the α-parvin-SDC4_cyt_ interaction, the *K*_D_ was 14.2 ± 6.1 nM, with *k*_a_ = (2.3 ± 0.8) x 10^4^ M^-1^ s^-1^ and *k*_d_ = (0.3 ± 0.1) x 10^–5^ s^-1^ (Figure 3F). Thus, despite SDC4_cyt_ binding both parvins with similar affinities, its faster dissociation rate from β-parvin suggests a more dynamic interaction than with α-parvin, which may reflect their distinct biological roles. For instance, α-parvin cannot compensate for β-parvin’s role in leading edge protrusions and serial sarcomere assembly in neonatal rat ventricular cardiomyocytes (Thievessen et al., 2022). Overall, our data suggest that syndecan-4 and β-parvin bind directly in cardiomyocytes. The interaction was specific to syndecan-4, and likely facilitated through the unique V-C2 region of syndecan-4. Syndecan-4 also appeared to bind α-parvin.

### 3.4 Syndecan-4 cytoplasmic tail interacts with several domains of β-parvin

**FIGURE 4:**
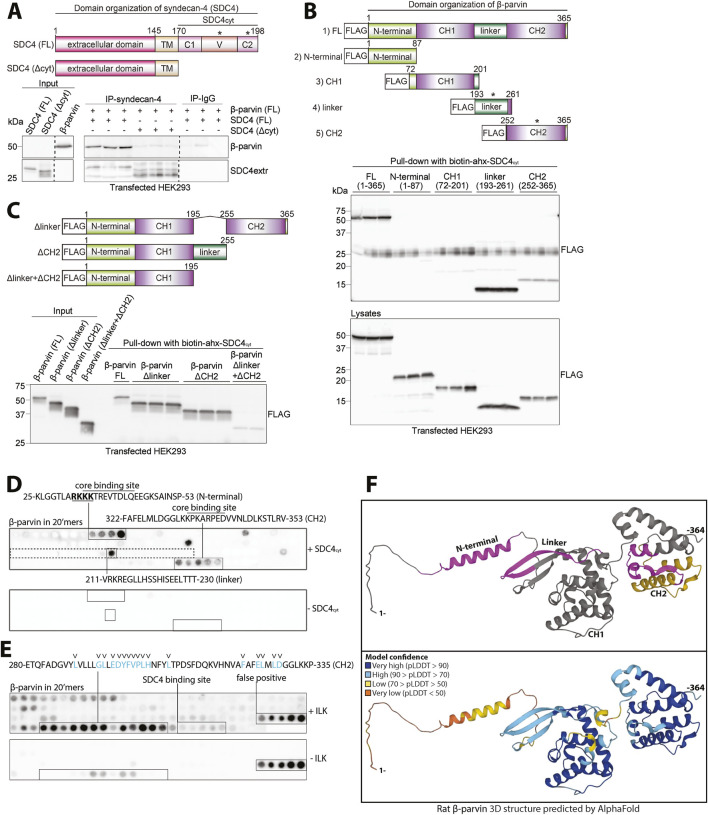
Syndecan-4 cytoplasmic tail interacts with several domains of β-parvin. **(A)** An illustration of the domain organization of syndecan-4 is given in the upper panel. Lysates from HEK293 cells co-transfected with FL rat β-parvin and with either mouse FL syndecan-4 (SDC4 FL) or syndecan-4 lacking its cytoplasmic tail (SDC4Δcyt) were used for immunoprecipitation of syndecan-4. Precipitation of syndecan-4 and β-parvin was detected by immunoblotting (n = 3 biological replicates). Dotted lines indicate representative lanes are a montage from the same exposed blot. Asterisks in the illustration indicate where binding was detected. **(B,C)** The biotinylated syndecan-4 cytoplasmic tail (biotin-ahx-SDC4_cyt_) was tested for binding against **(B)** five FLAG-tagged rat β-parvin constructs each covering a specific β-parvin domain (illustrated in the upper panel in B, asterisks indicate where binding was detected) and **(C)** rat FL or β-parvin deletions without either linker (Δlinker), CH2 domain (ΔCH2) or both linker and the CH2 domain (Δlinker+ΔCH2) (illustrated in upper panel in C). Precipitation of FLAG-tagged β-parvin FL protein or versions was detected with FLAG antibodies (n = 3 biological replicates). **(D)** 20′mers overlapping peptides covering FL β-parvin (rat) overlaid with biotin-ahx-SDC4_cyt_ and binding detected with biotin-HRP antibodies (n = 4 membranes). Peptide membranes without biotin-ahx-SDC4_cyt_ incubation were used as a negative control. The peptide sequence corresponding to the linker domain in β-parvin is boxed (dotted square). Overlined sequences correspond to the syndecan-4 core binding site (common sequence of the positive peptides). The nuclear localization signal of β-parvin (Olski et al., 2001) which syndecan-4 binds to at the N-terminal is underlined and in bold. **(E)** 20′mers overlapping peptides corresponding to FL β-parvin protein overlaid with recombinant ILK (S343D) and binding detected with ILK antibodies (n = 4 membranes). The blue letters and arrowheads mark β-parvin residues predicted to be involved in ILK binding based on the α-parvin-ILK interaction (Stiegler et al., 2012). Peptide membranes without ILK incubation were used as a negative control. **(F)** AlphaFold prediction of the protein folding structure of rat β-parvin (Varadi et al., 2021). Syndecan-4 and ILK binding sites are highlighted in magenta and yellow, respectively (top panel). The per-residue model confidence is estimated on a scale from 0–100 pLDDT, given on a color scale (lower panel).

To verify that β-parvin binds to the cytoplasmic tail of syndecan-4, we used HEK293 cells, which exhibited low levels of endogenous syndecan-4 and β-parvin (Figure 4A, input). Lysates from HEK293 cells co-transfected with rat β-parvin and either mouse FL syndecan-4 (SDC4 FL) or syndecan-4 without the cytoplasmic tail (SDC4Δcyt) (constructs schematic illustrated in Figure 4A, upper panel) were used to perform IP of syndecan-4. As expected, the β-parvin interaction was lost when the cytoplasmic tail of syndecan-4 was absent (Figure 4A, lower panel).

The parvins are characterized by an N-terminal region and two calponin homology (CH) domains separated by a linker region (Olski et al., 2001) (illustrated in Figure 4B, upper panel). Therefore, to map the corresponding syndecan-4 binding in β-parvin, five flag-tagged β-parvin constructs were designed and expressed in HEK293 cells. These were as follows: 1) full-length β-parvin (FL), 2) N-terminal domain, 3) CH1 domain, 4) linker domain, and 5) CH2 domain (illustrated in Figure 4B, upper panel). Pull-down experiments with the biotinylated SDC4_cyt_ peptide showed that syndecan-4 bound equally well to the linker domain as to the FL β-parvin protein (Figure 4B, upper immunoblot panel). Some syndecan-4 binding was also observed to the CH2 domain, whereas the N-terminal domain and the CH1 domain did not show any syndecan-4 binding (Figure 4B, upper immunoblot panel). Notably, the N-terminal domain migrated at approximately twice the expected mass and also yielded fainter lower molecular weight bands (Figure 4B, lower immunoblot panel). This could suggest dimerization, a trait previously proposed for the CH1 domain of α-parvin (Wang et al., 2008) and a possible hindrance for syndecan-4 binding. Next, we investigated whether deletion of the linker domain (Δlinker), CH2 domain (ΔCH2), or both domains (Δlinker + ΔCH2) abolished syndecan-4 binding (illustrated in Figure 4C, upper panel). Pull-down experiments with biotinylated SDC4_cyt_ showed that deletion of both domains (Δlinker + ΔCH2) was necessary to reduce syndecan-4 binding, however, some weak binding was still retained within the construct (Figure 4C, immunoblot panel). The latter observation suggests that there might be additional binding sites outside the linker and CH2 domain.

To map the syndecan-4-β-parvin interaction more precisely, full-length β-parvin was synthesized as 20′mer overlapping peptides with three residue offsets on membranes. By overlaying the membrane with a biotinylated SDC4_cyt_ peptide and immunoblotting with HRP-conjugated biotin antibodies, we found syndecan-4 binding to 25-KLGGTLARKKKTREVTDLQEEGKSAINSP-53 in the N-terminal domain, 211-VRKREGLLHSSHISEELTTT-230 in the linker domain and 322-FAFELMLDGGLKKPKARPEDVVNLDLKSTLRV-353 in the CH2 domain of β-parvin (Figure 4D). When we overlaid SDC4_cyt_ onto 20-mer overlapping α-parvin peptides, we found syndecan-4 binding to 31-GKLGGTLARRKKAKEVSEFQEEGMNAINL-59 in the N-terminal domain and 217-VVQKREGILQSRQIQEEITGNTE-239 in the linker domain of α-parvin, but no binding in the CH2 domain (Supplementary Figure S3A). Alignment of the three syndecan-4 binding sites in β-parvin with the corresponding sequences in α-parvin, showed similar sequences (Supplementary Figure S3B). However, the presence of a proline in the corresponding region of the SDC4_cyt_-β-parvin (CH2) core binding site in α-parvin might explain the lack of syndecan-4 binding to this region (Supplementary Figure S3B, arrowhead in lower panel).

Notably, the CH2 domain of β-parvin has also been described to bind to ILK, a partner in the IPP complex (Yamaji et al., 2001). To analyze whether the ILK and syndecan-4 binding to β-parvin overlapped, the β-parvin membranes were overlaid with recombinant ILK (S343D). Recombinant ILK bound a few amino acids upstream of the syndecan-4 core binding site (Figure 4E, residues previously suggested to be essential for the ILK-β-parvin interaction are shown in blue and with arrowheads (Stiegler et al., 2012), indicating that syndecan-4 and ILK are potentially able to bind to the β-parvin CH2 domain concomitantly.

Lastly, the identified binding sites were pinned down in the 3-dimensional rat β-parvin structure predicted by AlphaFold (Varadi et al., 2021). The three syndecan-4 binding sites (magenta) and the ILK binding site (yellow) all appeared to be located on the surface of β-parvin (Figure 4F, upper panel). The N-terminal and linker binding sites of syndecan-4 were in proximity, whereas the CH2 binding site was more distant and closer to the ILK binding site (Figure 4F, upper panel). Notably, high model confidence of the β-parvin structure was predicted by AlphaFold, except for its N-terminal domain and a part of the linker region (Figure 4F, high confidence in blue, lower panel). AlphaFold prediction of α-parvin showed similarities with the β-parvin structure (Supplementary Figure S3C).

Altogether, our data indicate that syndecan-4 binds directly to β-parvin through several binding sites and that syndecan-4 might be able to bind β-parvin while in the IPP complex.

### 3.5 Syndecan-4 is required for partial membrane anchoring of β-parvin in both sexes

**FIGURE 5:**
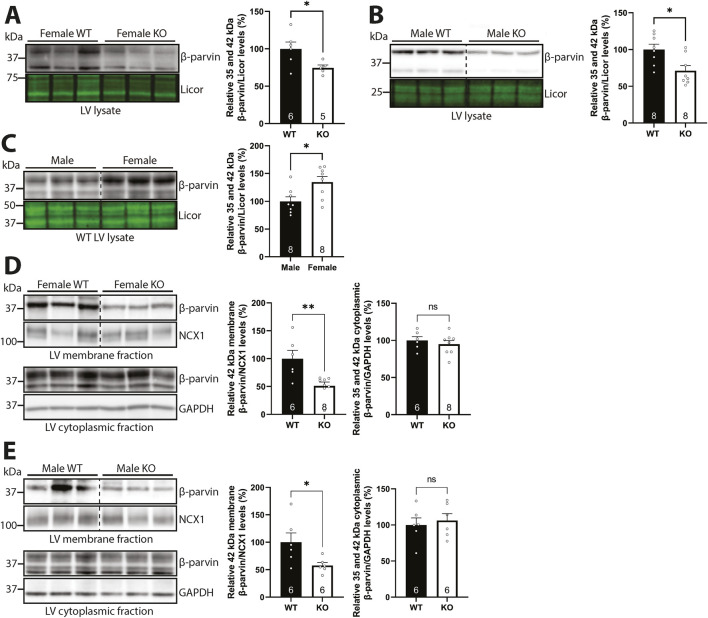
Syndecan-4 is required for partial membrane anchoring of β-parvin in both sexes. Immunoblots of β-parvin in **(A)** female and **(B)** male syndecan-4 KO and WT LVs, and **(C)** female and male WT LVs (n = 5-8 hearts). Specificity of β-parvin bands using antibody-blocking peptides is shown in Supplementary Figure S2A, left panel. **(D,E)** Immunoblots of β-parvin in membrane- and cytoplasmic-enriched fractions of LV tissue from female and male syndecan-4 KO and WT mice (n = 6-8 hearts). Enrichment of fractions is shown in Supplementary Figure S4F,G. All values are presented as mean percentages ±SEM, normalized to licor or subcellular marker levels, before being related to levels in lysates or subcellular fractions of female or male WT LVs (set to 100%). Dotted lines in B-E indicate representative lanes are a montage from the same exposed blot. Differences between the groups were analyzed by unpaired two-tailed *t*-tests **(A-E)** or Mann-Whitney *U*-tests **(D)** due to normal or non-normal distribution analyzed by Shapiro-Wilk testing (*p < 0.05, **p < 0.01, ns: not significant).

Next, we investigated the effect of syndecan-4 ablation on β-parvin levels. Lysates from male and female WT and syndecan-4 KO LVs were immunoblotted for β-parvin. In the absence of syndecan-4, the levels of β-parvin were lower in both sexes (25% in females and 29% in males) (Figures 5A,B). Notably, the levels of β-parvin were 34.5% higher in female WT compared to male WT LVs (Figure 5C), indicating the absolute reduction of β-parvin may be larger in female KO vs. male KO (Figure 5A vs. 5B). Consistent with the rat cardiomyocyte fractionation data (Figure 2B), the 42 kDa β-parvin band was prominent in the mouse membrane-enriched fraction (Figures 5D,E, upper panels), while both the 35 and 42 kDa bands were present in the cytoplasmic fractions (Figures 5D,E, lower panels). The different variants likely form due to alternative translational initiation sites at the N-terminus (Sepulveda and Wu, 2006). Quantification of respective bands revealed that the levels of membrane-located β-parvin were lower in both sexes (49% in females and 42% in males) in the absence of syndecan-4 (Figures 5D,E, upper panels), suggesting syndecan-4 may have a membrane anchoring role for β-parvin. The levels of cytoplasmic β-parvin were unaltered in both sexes (lower panels).

Taken together, our results suggest that syndecan-4 is required for the partial membrane anchoring of β-parvin in both sexes.

### 3.6 Syndecan-4 ablation results in sex-specific alterations in ILK and PINCH

**FIGURE 6:**
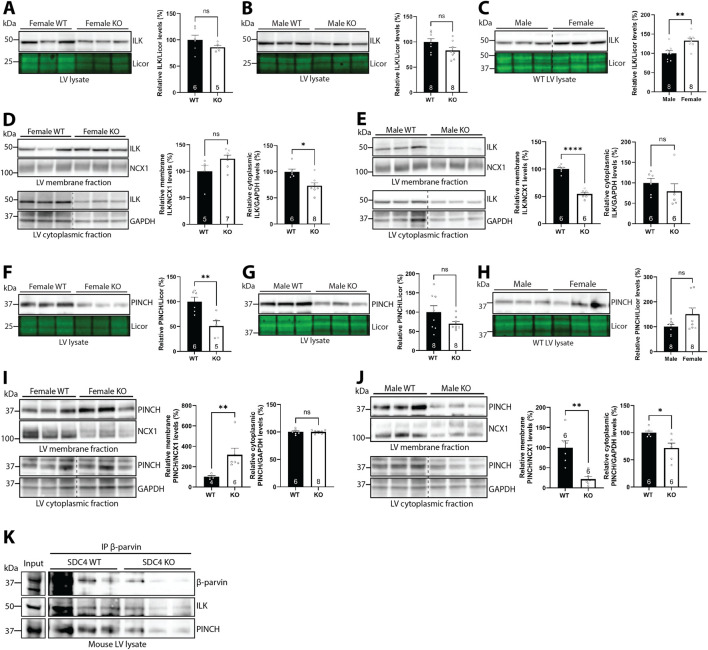
Syndecan-4 ablation results in sex-specific alterations in ILK and PINCH. Immunoblots of ILK in **(A–C)** LV lysates and **(D,E)** membrane and cytoplasmic-enriched fractions from female and male syndecan-4 KO and WT hearts (n = 5-8 hearts). Immunoblots of PINCH in **(F–H)** LV lysates and **(I,J)** membrane and cytoplasmic-enriched fractions from female and male syndecan-4 KO and WT hearts (n = 4-8 hearts). Enrichment of fractions is shown in Supplementary Figure S4F,G. Dotted lines in C-E and I-J indicate representative lanes are a montage from the same exposed blot. **(K)** Immunoprecipitation of β-parvin in male syndecan-4 WT and KO lysate, and immunoblotting using anti-β-parvin (D-2), ILK and PINCH (n = 3 hearts). Lysate input is shown on the left. All values are presented as mean percentages ±SEM, normalized to licor or subcellular marker levels, before being related to levels in LVs or subcellular fractions of female or male WT LVs (set to 100%) **(A–J)**. Differences between the groups were analyzed by unpaired two-tailed *t*-tests **(A–G,I,J)** or Mann-Whitney U-tests **(D,E,H,I)** due to normal or non-normal distribution analyzed by Shapiro-Wilk testing (*p < 0.05, **p < 0.01, ****p < 0.0001, ns: not significant).

Since β-parvin is part of the IPP complex with PINCH and ILK, we next investigated the effect of syndecan-4 ablation on ILK and PINCH. Previous work has shown that the assembly of the IPP complex protects the individual protein components from proteasomal degradation (Fukuda et al., 2003), and thus we expected similar changes in ILK and PINCH protein levels as β-parvin. Like β-parvin (Figures 5A–C), ILK LV levels trended towards being lower in both sexes (14% reduction in females and 17% reduction in males) in the absence of syndecan-4 (Figures 6A,B). ILK levels were 33% higher in female vs. male WT LVs (Figure 6C). However, fractionation analysis showed that ILK levels indeed varied between the sexes. While the level of membrane-located ILK trended towards being higher (23.9%) in female syndecan-4 KO (Figure 6D, upper panel), it was lower (45.5%) in males (Figure 6E, upper panel). Moreover, while female syndecan-4 KO exhibited 26.4% lower cytoplasmic ILK levels (Figure 6D, lower panel), these were not significantly altered in males (Figure 6E, lower panel). To assess whether the differential levels of ILK between the sexes could be due to differences in β1-integrin, a well-known ILK binding partner (Wu and Dedhar, 2001), we immunoblotted for β1-integrin. However, β1-integrin levels were not significantly different between genotypes or sexes, neither in the LVs nor the membrane-enriched fractions (Supplementary Figure S4A–E).

Like β-parvin (Figures 5A–C) and ILK (Figures 6A–C), PINCH levels were also lower in both sexes (49.4% reduction in females and 30% reduction in males) of syndecan-4 KO LVs (Figures 6F,G, trend in males), and there was a trend towards higher (50.8%) PINCH levels in female vs. male WT (Figure 6H). Like ILK, the level of membrane-located PINCH was also higher (216%) in the female KO, but lower (78.5%) in males (Figures 6I,J, upper panels, respectively), whereas cytoplasmic PINCH was lower (28.2%) in male syndecan-4 KO only (Figure 6J, lower panel). Enrichment of subcellular fractions was confirmed by subcellular compartment markers (Supplementary Figure S4F–G). A summary of percentage changes in IPP protein levels corresponding to Figures 5, 6 can be found in Supplementary Table S2.

Lastly, to test whether syndecan-4 was necessary for forming the IPP-complex, we immunoprecipitated β-parvin in male WT and syndecan-4 KO lysates. Lower levels of β-parvin were precipitated in the syndecan-4 KO, reflecting the lower protein levels present in these hearts (Figure 5B). Lower levels of ILK and PINCH was also co-precipitated in the KO, however, relative to the β-parvin levels precipitated, ILK and PINCH binding did not seem to be perturbed (Figure 6K).

Overall, our results suggest that syndecan-4 may be involved in maintaining the levels and localization of ILK and PINCH in a sex-specific manner. For example, whereas membrane-located ILK and PINCH levels trended towards being higher in female syndecan-4 KO, their levels were lowered in males. However, the assembly of the IPP complex did not seem to depend on syndecan-4.

### 3.7 Syndecan-4 ablation results in sex-specific alterations in subcellular levels of Rac1 protein and activity

**FIGURE 7:**
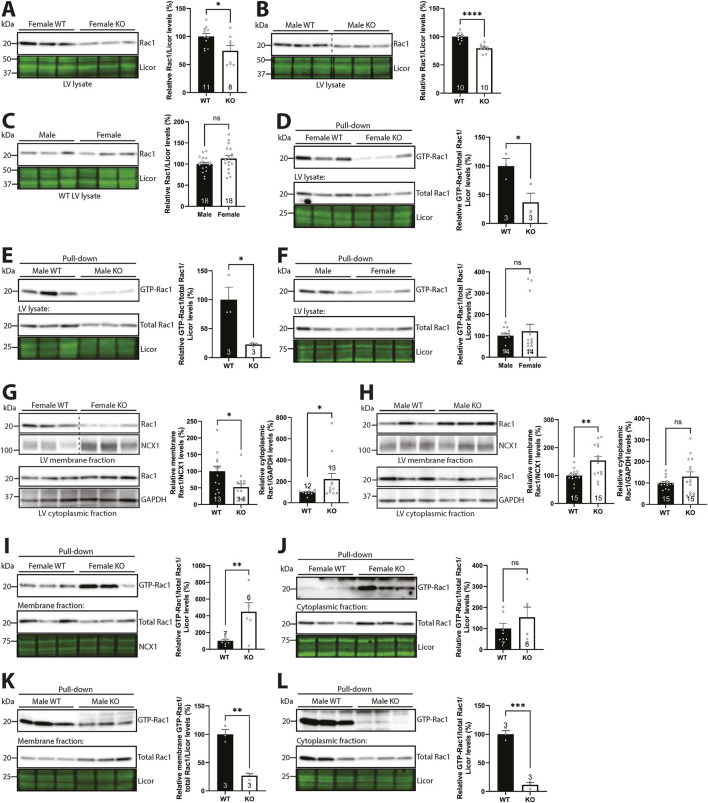
Syndecan-4 ablation results in sex-specific alterations in levels of membrane-located Rac1 and activity. Immunoblot of Rac1 in **(A)** female and **(B)** male syndecan-4 KO and WT LVs, and **(C)** female and male WT LVs (n = 8–18 hearts). Pull-down of GTP-bound active Rac1 in **(D)** female and **(E)** male syndecan-4 KO and WT LV lysates and **(F)** male and female WT mice (upper panels) and total Rac1 levels (bottom panels) (n = 3–14 hearts). Immunoblot of Rac1 in membrane- and cytoplasmic-enriched fractions of LVs from **(G)** female and **(H)** male syndecan-4 KO and WT mice (n = 12–15 hearts). Pull-down of GTP-bound active Rac1 in **(I,J)** female and **(K,L)** male membrane- and cytoplasmic-enriched fractions from syndecan-4 KO and WT LV lysates (upper panels) and total Rac1 levels (bottom panels) (n = 3-7 hearts). All values are presented as mean percentages ±SEM, normalized to licor or subcellular marker levels, before being related to levels in lysates or subcellular fractions of female or male WT LVs (set to 100%). Dotted lines in B and G indicate representative lanes are a montage from the same exposed blot. Differences between the groups were analyzed by unpaired two-tailed *t*-tests **(A–E,H,I,K,L)** or Mann-Whitney *U*-tests **(F,G,J)** due to normal or non-normal distribution analyzed by Shapiro-Wilk testing (*p < 0.05, **p < 0.01, ***p < 0.001, ****p < 0.0001, ns: not significant).

Since β-parvin has been found to be necessary for longitudinal cardiomyocyte growth through Rac1 (Thievessen et al., 2022), we next investigated whether Rac1 was also affected in the syndecan-4 KO. Immunoblotting showed lower Rac1 protein levels in both female (25%) and male (21%) syndecan-4 KO compared to their respective WT controls (Figures 7A,B, respectively). No differences were observed between the WT sexes (Figure 7C).

Rac1 belongs to the Rho-family of small GTPases, which are able to switch between inactive and active states via the binding of GDP or GTP, respectively (Bosco et al., 2009). To investigate whether the activity of Rac1 mirrored differences in its protein levels, we performed pull-down binding assays of GTP-bound active Rac1. Indeed, immunoblotting of Rac1 showed that female and male syndecan-4 KO LVs also exhibited lower (63% in females and 77% in males) levels of GTP-bound Rac1 (i.e., lower Rac1 activity) (Figures 7D,E, respectively). Equal levels of GTP-bound Rac1 were observed between the WT sexes (Figure 7F).

Since it is generally believed that the activation of Rac1, i.e., GTP attachment, happens at the membrane, and syndecan-4 has previously been found to be required for localizing Rac1 activity to the leading edge of fibroblasts (Bass et al., 2007; Bustelo et al., 2012), we next investigated the subcellular localization of Rac1 in the presence and absence of syndecan-4. Immunoblotting of Rac1 in membrane- and cytoplasmic-enriched fractions of syndecan-4 KO and WT LVs revealed that while the membrane-located Rac1 levels were 47.8% lower in female syndecan-4 KO (Figure 7G, upper panel), it was 54.1% higher in males (Figure 7H, upper panel). However, the Rac1 activity pattern was surprisingly different. Female syndecan-4 KO exhibited 348.8% higher levels of membrane-located GTP-bound Rac1/Rac1 (Figure 7I). Although not significant, a 53.3% increase in GTP-bound Rac1/Rac1 was also observed in the cytoplasmic-enriched fraction (Figure 7J). In contrast, 73.0% and 88.3% lower GTP-bound Rac1/Rac1 levels were observed in membrane- and cytoplasmic-enriched fractions, respectively, in male syndecan-4 KO LVs (Figures 7K,L, respectively). A summary of percentage changes in Rac1 protein or GTP-Rac1 pull-down levels in the syndecan-4 KO compared to respective WT animals corresponding to Figure 7 can be found in Supplementary Table S3.

Overall, although syndecan-4 ablation resulted in lower Rac1 activity in both sexes, sex-specific alterations in the subcellular levels of Rac1 protein and activity were observed.

### 3.8 Syndecan-4 ablation results in sex-specific alterations in Rac1 regulators

**FIGURE 8:**
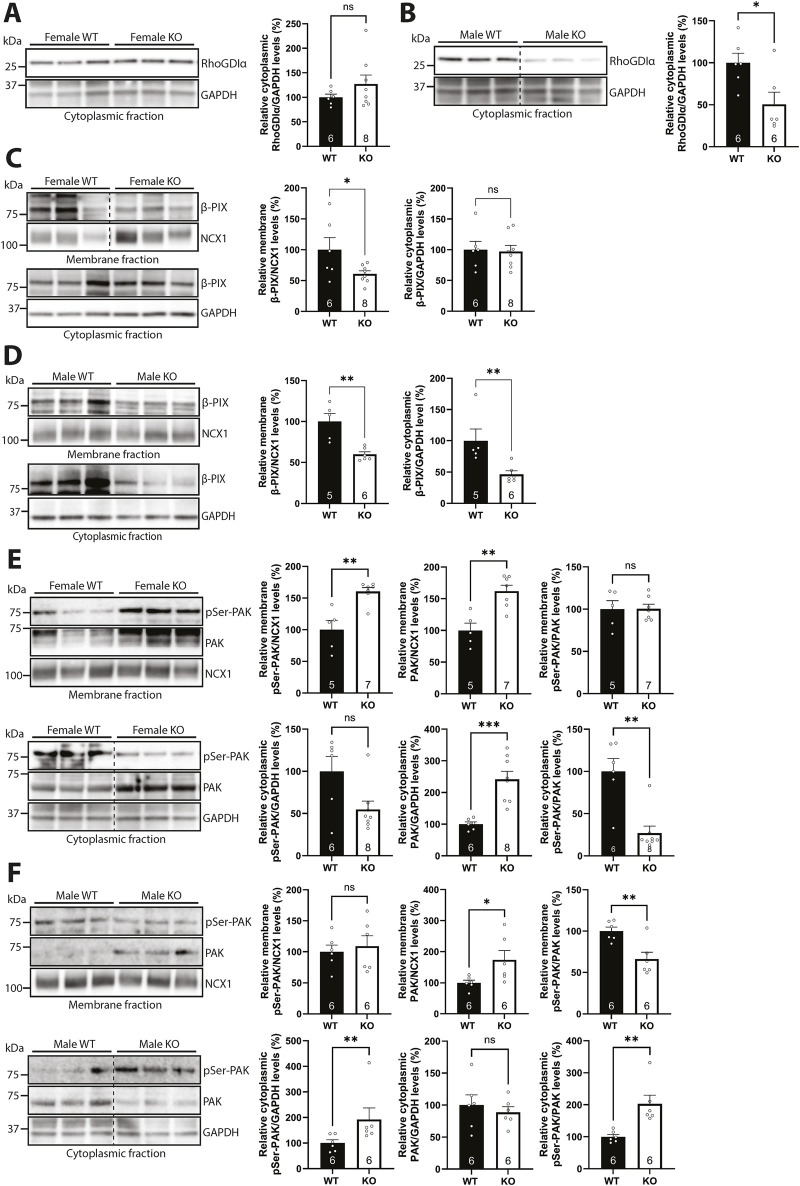
Syndecan-4 ablation results in sex-specific alterations in Rac1 regulators. **(A,B)** Immunoblot of RhoGDIα in cytoplasmic-enriched fractions from LVs of **(A)** female and **(B)** male syndecan-4 KO and WT mice (n = 6-8 hearts). **(C,D)** Immunoblot of β-PIX in membrane- and cytoplasmic-enriched fractions from **(C)** female and **(D)** male syndecan-4 KO and WT LVs (n = 5-8 hearts). **(E,F)** Immunoblot of phosphorylated PAK (pSer-PAK) (∼75 kDa) and PAK (∼70 kDa) in membrane- and cytoplasmic-enriched fractions from **(E)** female and **(F)** male syndecan-4 KO and WT mice (n = 5-8 hearts). Enrichment of fractions is shown in Supplementary Figure S4F,G. Dotted lines in B, C, E and F indicate representative lanes are a montage from the same exposed blot. All values are presented as mean percentages ±SEM. Immunoblots were normalized to subcellular marker levels, before being related to levels in subcellular fractions of female or male WT LVs (set to 100%). Differences between the groups were analyzed using unpaired two-tailed *t*-tests (A, C, D, E, and F) or Mann-Whitney *U*-tests (B, D, E, and F) due to normal or non-normal distribution analyzed by Shapiro-Wilk testing (*p < 0.05, **p < 0.01, ***p < 0.001, ns: not significant).

To better understand the sex-specific alterations in the subcellular levels of Rac1 protein and activity levels in the syndecan-4 KO heart, we first investigated Rho guanine dissociation inhibitor α (RhoGDIα), which is described to maintain GDP-bound inactive Rac1 in the cytoplasm (Bustelo et al., 2012). The levels of cytoplasmic RhoGDIα trended towards being higher (26.9%) in female syndecan-4 KO (Figure 8A) but were lower (49.5%) in male KO (Figure 8B), consistent with more Rac1 able to move to the membrane. Since RhoGDIα is a cytoplasmic protein (Dovas and Couchman, 2005), RhoGDIα was not detected in the membrane-enriched fractions (data not shown). RhoGDIα LV protein levels were not significantly altered between the genotypes and sexes (Supplementary Figure S5A–C).

We next investigated possible mechanisms that could result in differential Rac1 activation at the membrane. Guanine nucleotide exchange factors (GEFs) are able to activate Rac1 by stimulating the release of GDP, allowing GTP to bind, thus activating Rac1 (Matsuda et al., 2008). One such GEF is β-PIX, which has been shown to link β-parvin to Rac1 activation (Matsuda et al., 2008; Thievessen et al., 2022), and which we have previously identified as a possible partner to syndecan-4 in the cardiac syndecan-4 interactome (Mathiesen et al., 2019). Immunoblotting showed that β-PIX levels were lower (39% in females and 40% in males) in membrane-enriched fractions of syndecan-4 KO LVs of both sexes (Figures 8C,D, upper panels), suggesting that β-PIX levels alone are not the only mechanism underlying the sex-specific differences in Rac1 activity. However, sex-specific differences were observed in the cytoplasmic-enriched fractions, as the β-PIX level was unaltered in female KO, but 53.4% lower in male KO vs. respective WT controls (Figures 8C,D, lower panels). The β-PIX LV levels did not vary significantly between genotypes or sexes (Supplementary Figure S5D–F).

Although membrane-located β-PIX levels were lower in both sexes in the absence of syndecan-4 (Figures 8C,D), the levels of β-PIX available for Rac1 binding, and thus Rac1 activation, could still be different between the sexes. The serine/threonine kinase PAK has, for example, been found to occupy β-PIX, but its autophosphorylation releases it, allowing β-PIX to bind and activate Rac1 (ten Klooster et al., 2006). While immunoblotting showed higher levels (62% in females and 73% in males) of membrane-located PAK in syndecan-4 KO of both sexes (Figures 8E,F, upper panels, middle graph), suggesting less β-PIX is available for Rac1 activation, female syndecan-4 KO surprisingly also had 60.4% higher levels of phosphorylated PAK (pSer-PAK) in the membrane-enriched fraction (Figure 8E, upper panel, left graph). The latter could perhaps represent a compensatory mechanism to release more β-PIX to activate membrane-bound Rac1 in the female KO cardiomyocytes. In contrast, the cytoplasmic pSer-PAK/PAK ratio was found to be 73.0% lower in female KO, but 102.8% higher in male KO vs. respective WT controls (Figures 8E,F, lower panels). This difference could, in theory, allow a higher Rac1 activation in this subcellular fraction in males. The pSer-PAK/PAK LV levels were not significantly altered between genotypes and sexes (Supplementary Figure S5G–I). A summary of percentage changes in protein and phosphorylation levels of Rac1 regulators corresponding to Figure 8 can be found in Supplementary Table S4.

Taken together, the data indicate that cytoplasmic RhoGDIα levels were lower in male syndecan-4 KO, which may account for the higher membrane-bound Rac1 protein levels. Although β-PIX was lower in the membrane-enriched fractions of both sexes, the availability of β-PIX for Rac1 activation appeared to be higher in female syndecan-4 KO.

## 4 Discussion

**FIGURE 9:**
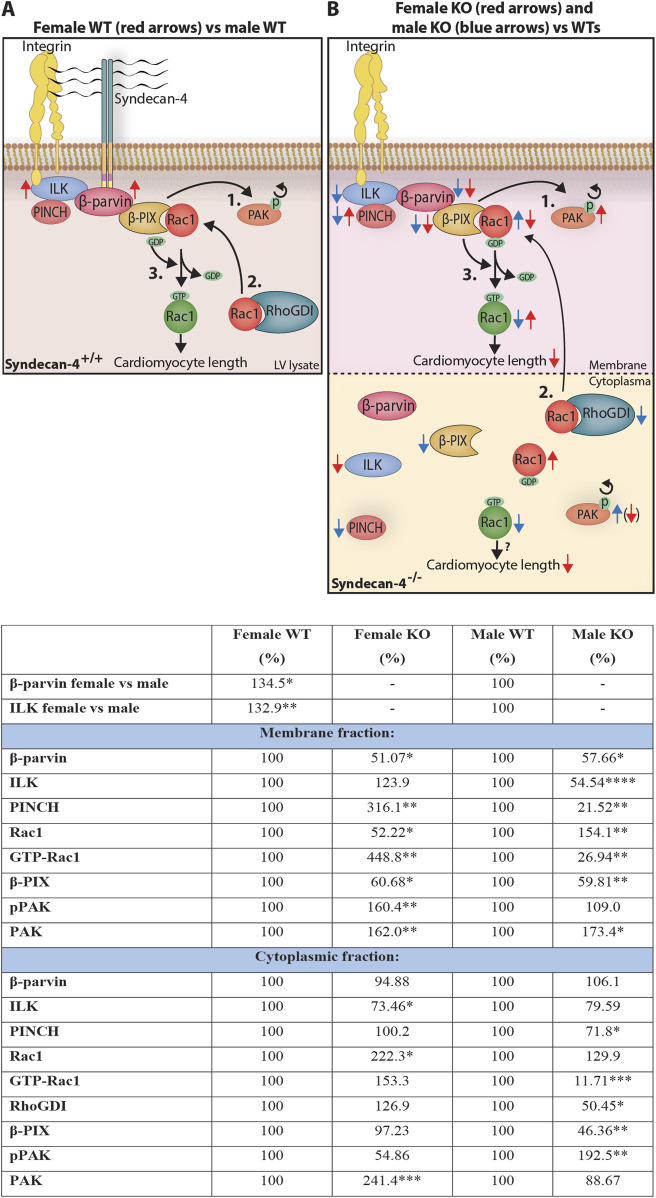
Sex differences in cardiomyocyte length and the syndecan-4-β-parvin-β-PIX-Rac1 axis. Illustration: An illustration of the differences observed in the syndecan-4-β-parvin-β-PIX-Rac1 axis in **(A)** WT LV lysates, and **(B)** LV membrane- and cytoplasmic-enriched fractions in syndecan-4 KO vs. WT. It has been described in the literature that the autophosphorylation of PAK causes its delocalization from β-PIX (1.), allowing Rac1 to bind upon its release from cytoplasmic RhoGDI (2.). β-PIX facilitates the exchange from GDP to GTP (3.), activating Rac1 which contributes to cardiomyocyte elongation. In A, red arrows represent changes in female compared to male WT hearts. In B, red and blue arrows represent KO alterations compared to respective WT hearts. Active (green) and inactive (red) Rac1 are distinguished by GTP and GDP attachment, respectively. Although Rac1 activation is believed to occur at the membrane, we also observed active Rac1 in the cytoplasm. Arrow in parenthesis indicates a strong trend only. See text for further discussion. Table: Changes in relative protein levels as illustrated in **(A,B)**. Differences between groups were tested with unpaired two-tailed t-tests or Mann-Whitney U-tests due to normal or non-normal distribution, respectively, analyzed by Shapiro-Wilk testing (*p < 0.05, **p < 0.01, ***p < 0.001, ****p < 0.0001).

Here we have shown that genetic ablation of syndecan-4 leads to shorter cardiomyocytes in female hearts only. In contrast, the geometry of male syndecan-KO cardiomyocytes was unaltered. The sex-specific difference in cardiomyocyte length was accompanied by alterations in the IPP complex and the β-parvin-β-PIX-Rac1 axis, the latter of which has been reported to control cardiomyocyte length (Thievessen et al., 2022). The syndecan-4-β-parvin interaction was mapped to surface-exposed sequences of the N-terminal, linker, and CH2 domains of β-parvin and the unique V-C2 cytoplasmic region of syndecan-4. Syndecan-4 ablation resulted in lower levels of membrane-localized β-parvin and its binding partner β-PIX in both sexes. However, Rac1, known for its involvement in cell size regulation, and its regulators, RhoGDIα and the serine/threonine kinase PAK, were differentially altered between the KO sexes. A summary of the observed sex differences in the WT and syndecan-4 KO heart is presented in Figures 9A,B.

Syndecan-4 was found to bind to three different sites in β-parvin. Interestingly, one of these sites was identified in the CH2 domain of β-parvin. This domain has been suggested to be essential for the recruitment of β-parvin to focal adhesions through its interaction with ILK (Yamaji et al., 2001), but also through the focal adhesion components paxillin and α-actinin (Yamaji et al., 2004; Stiegler et al., 2012). Since syndecan-4 is involved in the formation of focal adhesions in fibroblasts and also connects to both paxillin and α-actinin (Woods et al., 1986; Woods et al., 2000; Denhez et al., 2002; Greene et al., 2003), we speculate that syndecan-4 is involved in targeting β-parvin, or perhaps the whole IPP complex, to adhesion sites such as costameres in cardiomyocytes. This is supported by our findings showing that syndecan-4 and ILK bound to different amino acid sequences in the CH2 domain of β-parvin, suggesting syndecan-4 can bind β-parvin while in the IPP complex.

We also observed syndecan-4 binding to the unusually long 60 amino acid linker domain of β-parvin, which has been proposed to have an additional role beyond merely serving as a linker between the CH domains (Gimona et al., 2002). For example, it has been proposed that this linker domain may serve as an intramolecular regulator of the CH1 domain by masking its binding sites (Vakaloglou et al., 2012). Thus, we cannot exclude that syndecan-4 also has a regulatory role for other interactions, by, e.g., freeing up the CH1 or CH2 domain, while occupying the linker domain. The CH1 domain has, for instance, been shown to be involved in Rac1 activation through α-PIX (Mishima et al., 2004).

Syndecan-4 also showed binding to the N-terminal domain of β-parvin by peptide arrays. The syndecan-4 binding site overlapped with a putative nuclear localization signal (NLS) (Olski et al., 2001), suggesting that syndecan-4 may also be involved in the nuclear translocation of β-parvin, or alternatively, in preventing its nuclear entry. Considering its short cytoplasmic tail (28 amino acids), it is unlikely that syndecan-4 binds to all three sites in β-parvin simultaneously unless this is facilitated by the 3-dimensional folding of β-parvin and/or through syndecan-4 oligomerization (Choi et al., 2005). AlphaFold predicted that all three syndecan-4 binding sites are exposed on the surface of β-parvin, and that only the N-terminal and linker regions may be in close enough proximity to form one binding site. However, the N-terminal domain is located in an area of low model confidence, which often overlaps with intrinsically disordered regions (Ruff and Pappu, 2021), suggesting that the N-terminal region might fold differently *in vivo*.

Although the ability of syndecan-4 and β-parvin to directly interact (amino acid to amino acid) is unlikely to differ between the sexes, post-translational modifications of either protein, potentially modulating the interaction, may be sex-dependent, which we have not investigated in this study. We did, however, find that syndecan-4 seems to be required for the partial membrane anchoring of β-parvin in both sexes. In contrast to β-parvin, we found sex-specific alterations in the levels and localization of PINCH and ILK when syndecan-4 was ablated. It will be interesting to confirm our subcellular fractionation results with complementary methods, such as immunofluorescent staining. Nonetheless, our findings indicate that the IPP complex is differentially altered between the sexes and that female and male syndecan-4 KO likely have different mechano-transduction properties. The IPP complex forms in the cytosol before localizing to the membrane (Zhang et al., 2002; Legate et al., 2006), and it is reported that its components are more susceptible to degradation if they fail to assemble (Fukuda et al., 2003). Interestingly, PKCα, a well-known binding partner to the unique variable region of syndecan-4 (Keum et al., 2004), has been found to be important in the IPP-complex assembly, where inhibition of PKCα prevents PINCH binding to ILK (Zhang et al., 2002). PKCα membrane localization and activation are lower when syndecan-4 is absent (Keum et al., 2004), and it is therefore likely that syndecan-4 has at least an indirect role in the assembly of the IPP complex.

Both syndecan-4 and the integrins localize to focal adhesions and costameres (Ross and Borg, 2001; VanWinkle et al., 2002), and the two protein families have been suggested to cooperate in various adhesion events (Saoncella et al., 1999; Whiteford and Couchman, 2006; Morgan et al., 2007; Whiteford et al., 2007). Syndecan-4 has also been found to be involved in endocytosis and recycling of integrin (Bass et al., 2011), where syndecan-4 phosphorylation is a control point for the recycling process (Morgan et al., 2013). However, despite a persistent effort from the community, it has been difficult to pinpoint proteins that directly link the integrins and syndecans. It is possible that the direct syndecan-4-β-parvin interaction we have identified in this study bridges syndecan-4 and integrin signaling.

Furthermore, we presently showed that our previous finding of smaller cardiomyocytes in female syndecan-4 KO (Støle et al., 2022) is due to an average 6 µm reduction in cardiomyocyte length. Cardiomyocyte elongation has been found to be regulated by Rac1 through β-parvin binding to β-PIX (Thievessen et al., 2022). Rac1 has also been shown to be activated by the β-parvin-β-PIX axis in C2C12 cells (Matsuda et al., 2008) and to be involved in cardiomyocyte elongation during embryonic development (Leung et al., 2014). Consistent with a role for Rac1 in cardiomyocyte elongation, we found sex-specific alterations in the subcellular levels of Rac1 protein and activity upon syndecan-4 ablation. Surprisingly, female syndecan-4 KO exhibited higher levels of membrane-located GTP-bound Rac1 (i.e., higher Rac1 activity), perhaps reflecting a compensatory mechanism in an attempt to increase cardiomyocyte length. In contrast, the membrane-located GTP-bound Rac1/Rac1 levels were lower in syndecan-4 KO males. We also found sex differences in regulators of Rac1 activity upon syndecan-4 ablation. The level of RhoGDIα, which keeps Rac1 inactive in the cytoplasm (Bustelo et al., 2012), was lower in male syndecan-4 KO vs. female, and could thus explain the higher level of membrane-bound Rac1 protein in KO males. However, β-PIX, which regulates the GDP-GTP exchange, and thus Rac1 activation, was lower in the membrane fractions of both syndecan-4 KO sexes, suggesting β-PIX is likely merely involved in the overall lower levels of GTP-bound Rac1 and not the sex-dependent reduction in cardiomyocyte length upon syndecan-4 ablation. We also investigated the levels of the serine/threonine kinase PAK, which competitively binds to β-PIX, but is released from β-PIX upon phosphorylation (pSer-PAK), allowing more β-PIX to bind and activate Rac1 (ten Klooster et al., 2006). Consistent with the higher membrane-located Rac1 activity, female syndecan-4 KO LVs had higher levels of both pSer-PAK and PAK in the membrane-enriched fraction. In contrast, male syndecan-4 KO had lower levels of pSer-PAK/PAK in the membrane-enriched fraction, but high levels in the cytoplasmic-enriched fraction, which in theory could allow a higher Rac1 activation in this fraction in males.

We cannot exclude the possibility that other Rho GTPases upstream of Rac1, such as RhoA and RhoG (Elfenbein and Simons, 2013), GEFs or other regulators of Rac1 activity are differentially regulated between the syndecan-4 KO sexes, contributing to the maintained cardiomyocyte length in males. We and others have previously found that both RhoA and RhoG associate with syndecan-4 (Thodeti et al., 2003; Brooks et al., 2012; Shin et al., 2013; Mathiesen et al., 2019). RhoA and RhoG activity is regulated by the phosphorylation state of RhoGDIα, which again is regulated by the syndecan-4 binding partner PKCα (Keum et al., 2004; Elfenbein et al., 2009; Dovas et al., 2010). Interestingly, PAK can also phosphorylate RhoGDIα contributing to the dissociation of Rac1 (Dovas et al., 2010). We did not investigate alterations in syndecan-4 binding to GEF Tiam1 (Keller-Pinter et al., 2017), since Tiam1 was not identified in the cardiac syndecan-4 interactome (Mathiesen et al., 2019).

Although we have not investigated the physiological consequences of the molecular sex differences we observed in the syndecan-4 KO, we speculate that it may result in a heart that is less able to cope with an increase in stressors, more so in females than males. β-parvin has previously been found to be necessary for actin cytoskeleton organization and serial sarcomere assembly, and its loss results in reduced force generation during contraction (Thievessen et al., 2022). The same effect may be seen in the female syndecan-4 KO mice, upstream of β-parvin, which would reveal a novel role for syndecan-4 in contractile force generation in the female heart. Other sex-specific differences in syndecan-4 KO mice have also been found where females have, for example, been shown to display an increase in fatty acids and lipids with a high-fat diet compared to males, and have delayed wound healing (Echtermeyer et al., 2001; De Luca et al., 2019). Shed syndecan-4 has also been shown to associate with myocardial infarction in women only (Solbu et al., 2018). Despite the sex differences observed in cardiomyocyte length at baseline, we cannot exclude the possibility that syndecan-4 is involved in cardiomyocyte size regulation in both sexes upon stress. Without challenge, syndecan-4 KO (Finsen et al., 2011), as well as the β-parvin KO (Thievessen et al., 2022) and cardiomyocyte-specific Rac1 KO (Satoh et al., 2006) mice do not have a cardiac phenotype. However, upon challenges such as aortic banding, exercise training, or angiotensin treatment, the hearts of syndecan-4 (Finsen et al., 2011), β-parvin (Thievessen et al., 2022), and cardiomyocyte-specific Rac1 KO (Satoh et al., 2006) mice are not able to compensate with hypertrophy development. It is thus possible that the syndecan-4-β-parvin-β-PIX-Rac1 axis is involved in cardiomyocyte length regulation in both sexes upon hypertrophic stimulation or overload of the adult heart. It is also possible that cardiomyocyte size is regulated through the syndecan-4-α-parvin interaction under pathological conditions. α-parvin expression is reported to be low in the adult heart muscle (Thievessen et al., 2022), but is found to be essential for heart development (Montanez et al., 2009). Notably, α-parvin co-precipitated with cardiac syndecan-4 in our previous interactome study (Mathiesen et al., 2019), and here, we confirmed their interaction by mapping syndecan-4 binding to its N- and linker domains.

While the alterations in the molecular pathways tested coexist with a phenotype of shorter cardiomyocytes in the female syndecan-4 KO heart, we have not tested for a direct relationship between the two. Manipulating β-parvin or Rac1 in syndecan-4 KO cardiomyocytes prior to geometry assessments may yield answers to such questions. However, we postulate that syndecan-4 is necessary, at least partially, for the membrane anchoring of β-parvin, and thus, the overexpression of β-parvin in a syndecan-4 KO cardiomyocyte may not be sufficient to rescue the phenotype. Additionally, the membrane-anchoring of Rac1 has also been shown to depend on post-translational modifications, such as prenylation of the C-terminal tail (Winter-Vann and Casey, 2005; Bustelo et al., 2007). Whether syndecan-4 is involved in such modifications and whether they are sex-dependent remains to be elucidated.

Overall, we have shown that ablation of syndecan-4 leads to shorter cardiomyocytes in females only. The sex-specific difference in cardiomyocyte length was accompanied by sex-specific alterations in the IPP complex, β-parvin-β-PIX-Rac1 axis, and Rac1 regulators. The role of the syndecan-4-α/β-parvin interactions in the pressure-overloaded heart will be an interesting area for future studies.

## Data Availability

The original contributions presented in the study are included in the article/Supplementary Material, further inquiries can be directed to the corresponding author.

## Abbreviations

β-PIX, Rho guanine nucleotide exchange factor 7; CH, calponin homology; ECM, extracellular matrix; FL, full-length; HRP, horse radish peroxidase; IPP, ILK-PINCH-parvin; ILK, integrin-linked-kinase; LV, left ventricle; O/N, overnight; PINCH, particularly interesting new cysteine-histidine-rich protein; Rac1, ras-related C3 botulinum toxin substrate 1; SDC4, syndecan-4; SDC4cyt, cytoplasmic domain of syndecan-4.
