# Supplementary material for: Syndecan-4 interacts directly with β-parvin and regulates the ILK-PINCH-β-parvin complex, the β-parvin-β-PIX-Rac1 axis, and cardiomyocyte geometry in a sex-dependent manner
Source: Front Cell Dev Biol. 2025 Aug 29;13:1569185. doi: 10.3389/fcell.2025.1569185 (PMC12447578; doi:10.3389/fcell.2025.1569185)
Supplement: Supplementary file 1 [file Table1.pdf]

Supplementary Table 1. Body weight, in grams (g), of male and female syndecan-4 KO and WT mice, 12-15 weeks of age (n=11-26). The range (min-max), standard deviation, and SEM are given. Differences between groups were tested with unpaired two-tailed t-tests due to normal distribution, analyzed by Shapiro-Wilk testing (****p < 0.0001 male WT vs female WT, ††††p < 0.0001 male KO vs female KO).

	**Male WT** (n=23)	**Male KO** (n=19)	**Female WT** (n=26)	**Female KO** (n=11)
**Mean (g)**	27.25	27.95	20.59****	20.38††††
**Range (min-max)**	24.2-30.7	23.5-30.4	18.7-22.8	17.5-21.9
**Std. Deviation**	1.75	1.79	1.14	1.34
**SEM**	0.36	0.41	0.22	0.40

**Supplementary Table 2:** Relative protein levels in KO LVs compared to respective WT animals (Set to 100%), corresponding to figures 5-6, and supplementary figure 4. Differences between groups were tested with unpaired two-tailed t-tests or Mann-Whitney U-tests due to normal or non-normal distribution, respectively, analyzed by Shapiro-Wilk testing (*p< 0.05, **p< 0.01, ****p < 0.0001).				
	**Female WT (%)**	**Female KO (%)**	**Male WT (%)**	**Male KO (%)**
**LV lysate, β-parvin (Fig. 5A)**	100	74.57*	-	-
**LV lysate, β-parvin (Fig. 5B)**	-	-	100	71.13*
**LV lysate, β-parvin (Fig. 5C)**	134.5*	-	100	-
**LV membrane fraction, β-parvin (Fig. 5D, upper panel)**	100	51.07**	-	-
**LV cytoplasmic fraction, β-parvin (Fig. 5D, lower panel)**	100	94.88	-	-
**LV membrane fraction, β-parvin (Fig. 5E, upper panel)**	-	-	100	57.66*
**LV cytoplasmic fraction, β-parvin (Fig. 5E, lower panel)**	-	-	100	106.1
**LV lysate, ILK (Fig. 6A)**	100	86.02	-	-
**LV lysate, ILK (Fig. 6B)**	-	-	100	83.20
**LV lysate, ILK (Fig. 6C)**	132.9**	-	100	
**LV membrane fraction, ILK (Fig. 6D, upper panel)**	100	123.9	-	-
**LV cytoplasmic fraction, ILK (Fig. 6D, lower panel)**	100	73.46*	-	-
**LV membrane fraction, ILK (Fig. 6E, upper panel)**	-	-	100	54.54****
**LV cytoplasmic fraction, ILK (Fig. 6E, lower panel)**	-	-	100	79.59
**LV lysate, PINCH (Fig. 6F)**	100	50.65**	-	-
**LV lysate, PINCH (Fig. 6G)**	-	-	100	70.07
**LV lysate, PINCH (Fig. 6H)**	150.8	-	100	-
**LV membrane fraction, PINCH (Fig. 6I, upper panel)**	100	316.1**	-	-
**LV cytoplasmic fraction, PINCH (Fig. 6I, lower panel)**	100	100.2	-	-

**LV membrane fraction, PINCH (Fig. 6J, upper panel)**	-	-	100	21.52**
**LV cytoplasmic fraction, PINCH (Fig. 6J, lower panel)**	-	-	100	71.80*
**LV lysate, β1-integrin (Suppl. Fig. 4A)**	100	112.3		
**LV lysate, β1-integrin (Suppl. Fig. 4B)**			100	106.9
**LV lysate, β1-integrin (Suppl. Fig. 4C)**	129.4		100	
**LV membrane fraction, β1-integrin (Suppl. Fig. 4D)**	100	117.3		
**LV membrane fraction, β1-integrin (Suppl. Fig. 4E)**			100	110.0

Supplementary Table 3: Relative Rac1 protein levels in LV lysate or GTP-Rac1 pull-downs compared to respective WT animals (set to 100%), corresponding to figure 7. Differences between groups were tested with unpaired two-tailed t-tests or Mann-Whitney U-tests due to normal or non-normal distribution, respectively, analyzed by Shapiro-Wilk testing (*p< 0.05, **p< 0.01, ***p< 0.001, ****p < 0.0001).

	**Female WT (%)**	**Female KO (%)**	**Male WT (%)**	**Male KO (%)**
**LV lysate, Rac1 (Fig. 7A)**	100	74.57*	-	-
**LV lysate, Rac1 (Fig. 7B)**	-	-	100	79.43****
**LV lysate, Rac1 (Fig. 7C)**	113.3	-	100	-
**Pull-down in LV, Rac1 (active) (Fig. 7D)**	100	36.71*	-	-
**Pull-down in LV, Rac1 (active) (Fig. 7E)**	-	-	100	22.63*
**Pull-down in LV, Rac1 (active) (Fig. 7F)**	121.2	-	100	-
**LV membrane fraction, Rac1 (Fig. 7G, upper panel)**	100	52.22*	-	-
**LV cytoplasmic fraction, Rac1 (Fig. 7G, lower panel)**	100	222.3*	-	-
**LV membrane fraction, Rac1 (Fig. 7H, upper panel)**	-	-	100	154.1**
**LV cytoplasmic fraction, Rac1 (Fig. 7H, lower panel)**	-	-	100	129.9
**Pull-down in membrane fraction, Rac1 (active) (Fig. 7I)**	100	448.8**	-	-
**Pull-down in cytoplasmic fraction, Rac1 (active) (Fig. 7J)**	100	153.3	-	-
**Pull-down in membrane fraction, Rac1 (active) (Fig. 7K)**	-	-	100	26.94**
**Pull-down in cytoplasmic fraction, Rac1 (active) (Fig. 7L)**	-	-	100	11.71****

Supplementary Table 4: Relative protein levels in KO LVs compared to respective WT animals (Set to 100%), corresponding to figure 8 and supplementary figure 5. Differences between groups were tested with unpaired two-tailed t-tests or Mann-Whitney U-tests due to normal or non-normal distribution, respectively, analyzed by Shapiro-Wilk testing (*p< 0.05, **p< 0.01, ***p< 0.001, ****p < 0.0001).

	**Female WT (%)**	**Female KO (%)**	**Male WT (%)**	**Male KO (%)**
**LV cytoplasmic fraction, RhoGDIα (Fig. 8A)**	100	126.9	-	-
**LV cytoplasmic fraction, RhoGDIα (Fig. 8B)**	-	-	100	50.45*
**LV lysate, RhoGDIα (Suppl. Fig. 5A)**	100	59.74	-	-
**LV lysate, RhoGDIα (Suppl. Fig. 5B)**	-	-	100	108.7
**LV lysate, RhoGDIα (Suppl. Fig. 5C)**	139.5	-	100	-
**LV membrane fraction, β-PIX (Fig. 8C, upper panel)**	100	60.68*	-	-
**LV cytoplasmic fraction, β-PIX (Fig. 8C, lower panel)**	100	97.23	-	-
**LV membrane fraction, β-PIX (Fig. 8D, upper panel)**	-	-	100	59.81**
**LV cytoplasmic fraction, β-PIX (Fig. 8D, lower panel)**	-	-	100	46.63**
**LV lysate, β-PIX (Suppl. Fig. 5D)**	100	110.5	-	-
**LV lysate, β-PIX (Suppl. Fig. 5E)**	-	-	100	118.2
**LV lysate, β-PIX (Suppl. Fig. 5F)**	102.4	-	100	-
**LV membrane fraction, pSerPAK (Fig. 8E, upper panel)**	100	160.4**	-	-
**LV membrane fraction, PAK (Fig. 8E, upper panel)**	100	162.0**	-	-
**LV membrane fraction, pSerPAK/PAK (Fig. 8E, upper panel)**	100	100.5	-	-
**LV cytoplasmic fraction, pSerPAK (Fig. 8E, lower panel)**	100	54.86	-	-
**LV cytoplasmic fraction, PAK (Fig. 8E, lower panel)**	100	241.4***	-	-
**LV cytoplasmic fraction, pSerPAK/PAK (Fig. 8E, lower panel)**	100	26.99**	-	-
**LV membrane fraction, pSerPAK (Fig. 8F, upper panel)**	-	-	100	109.0
**LV membrane fraction, PAK (Fig. 8F, upper panel)**	-	-	100	173.4*
**LV membrane fraction, pSerPAK/PAK (Fig. 8F, upper panel)**	-	-	100	66.18**

**LV cytoplasmic fraction, pSerPAK (Fig. 8F, lower panel)**	-	-	100	192.5**
**LV cytoplasmic fraction, PAK (Fig. 8F, lower panel)**	-	-	100	88.67
**LV cytoplasmic fraction, pSerPAK/PAK (Fig. 8F, lower panel)**	-	-	100	202.8**
**LV lysate, pPAK (Suppl. Fig. 5G)**	100	71.76	-	-
**LV lysate, PAK (Suppl. Fig. 5G)**	100	81.84	-	-
**LV lysate, pPAK (Suppl. Fig. 5H)**	-	-	100	93.48
**LV lysate, PAK (Suppl. Fig. 5H)**	-	-	100	82.18
**LV lysate, pPAK (Suppl. Fig. 5I)**	120.5	-	100	-
**LV lysate, PAK (Suppl. Fig. 5I)**	99.01	-	100	-
