# Supplementary material for: Syndecan-4 interacts directly with β-parvin and regulates the ILK-PINCH-β-parvin complex, the β-parvin-β-PIX-Rac1 axis, and cardiomyocyte geometry in a sex-dependent manner
Source: Front Cell Dev Biol. 2025 Aug 29;13:1569185. doi: 10.3389/fcell.2025.1569185 (PMC12447578; doi:10.3389/fcell.2025.1569185)

Full length blots for figure 1C

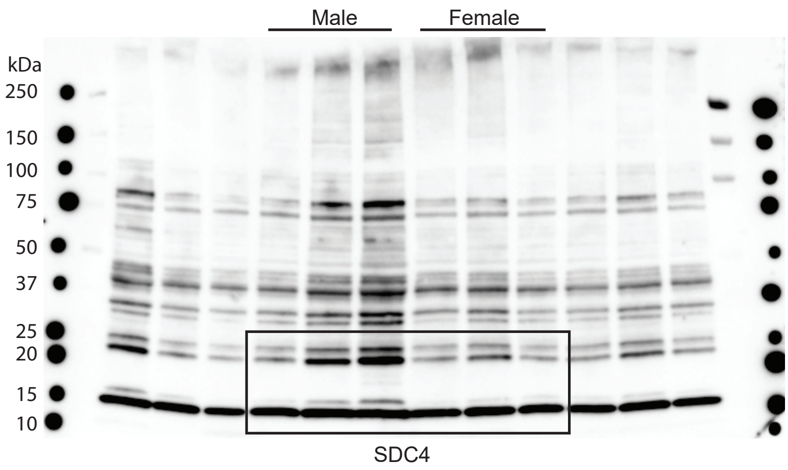

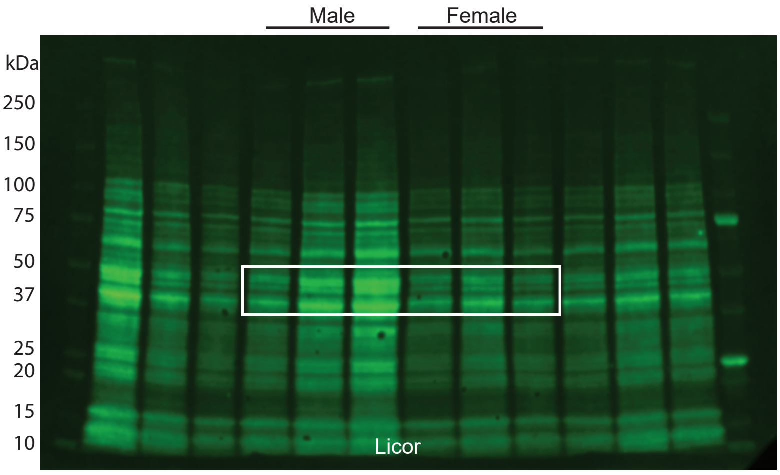

Full length blots for figure 2A

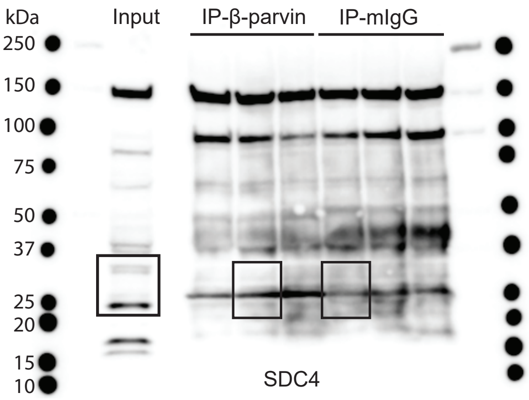

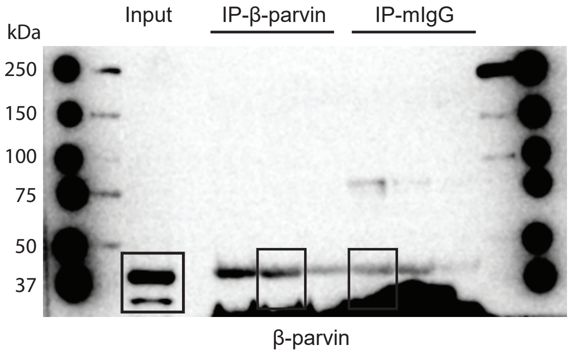

Full length blots for figure 2B

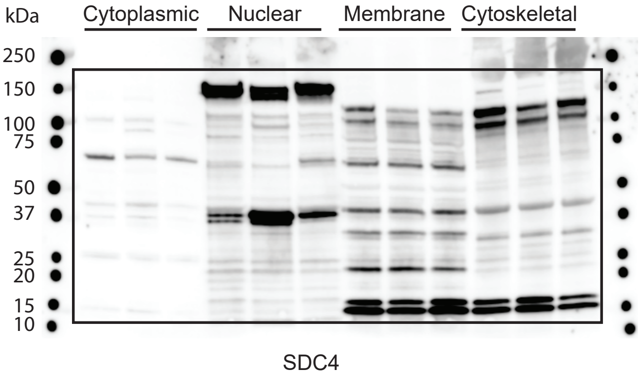

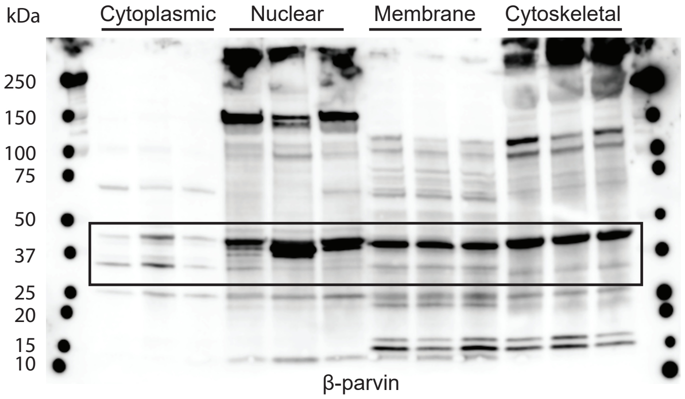

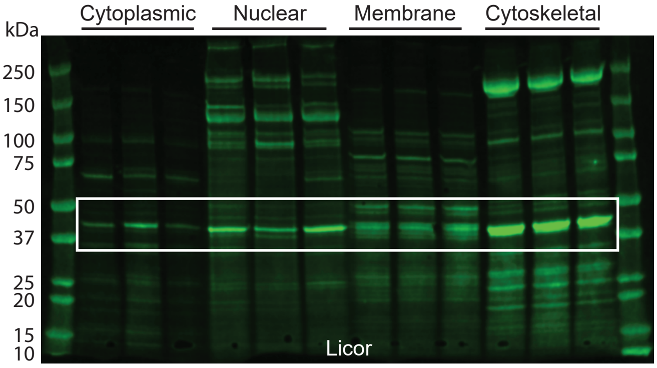

Full length blots for figure 3A

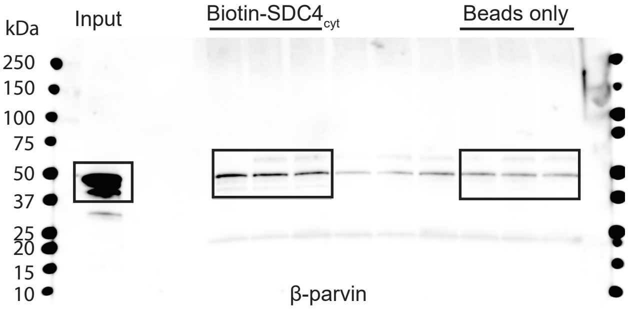

Full length blots for figure 4A

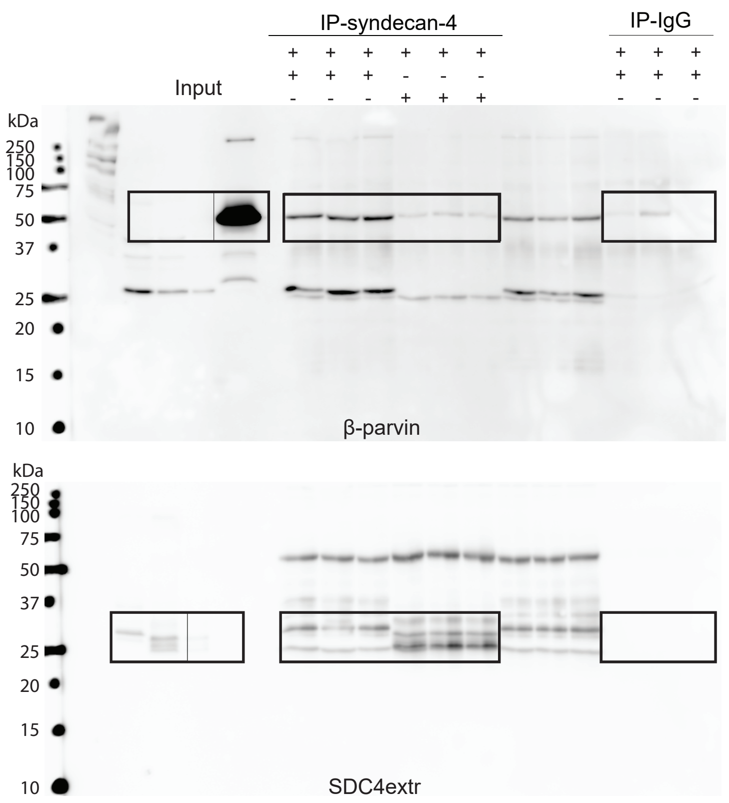

Full length blots for figure 4C

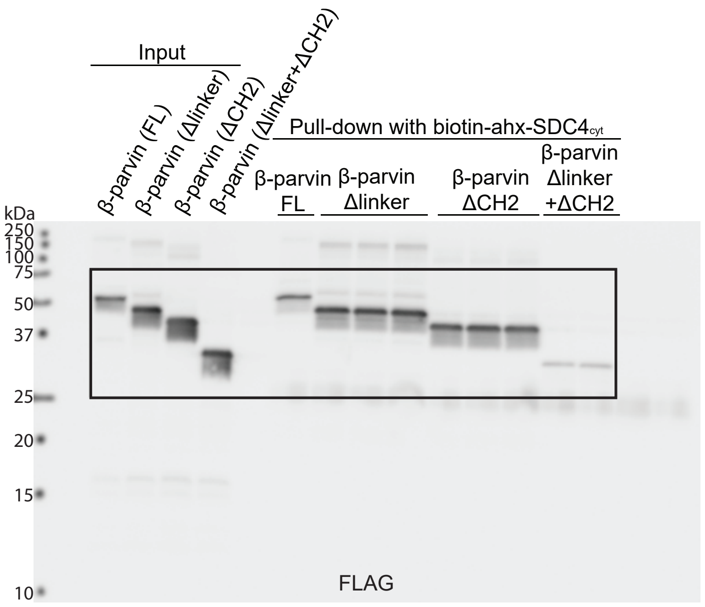

Full length blots for figure 4B

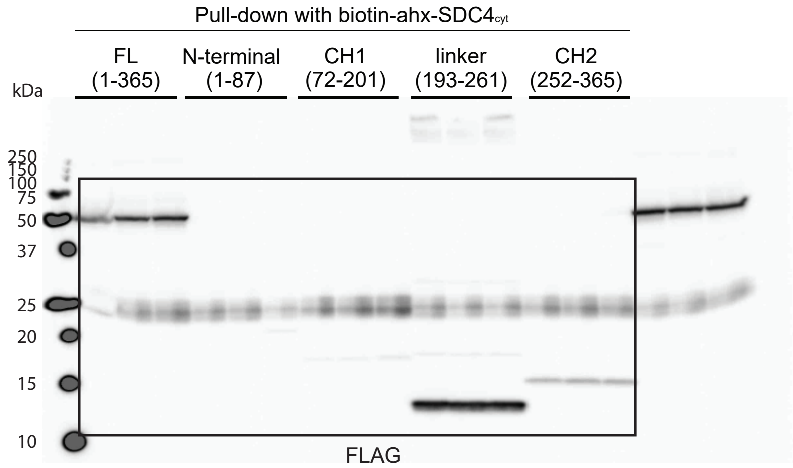

Lysates

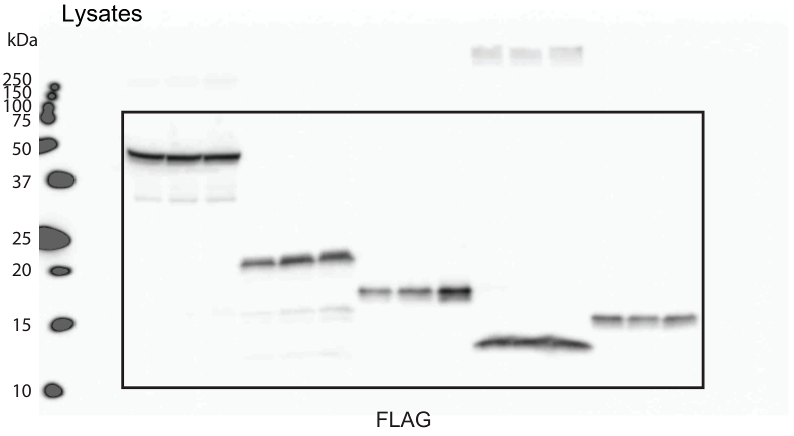

Full length blots for figure 5A

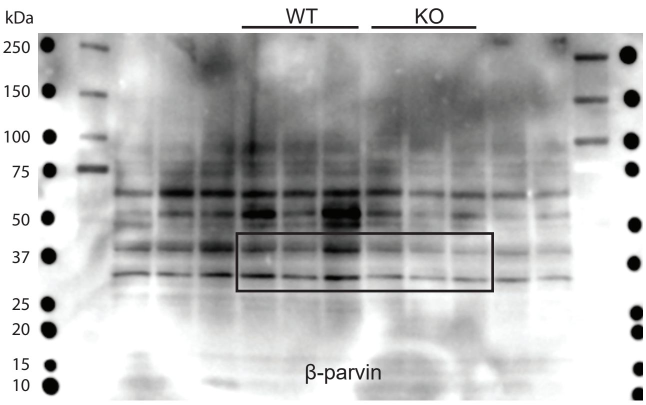

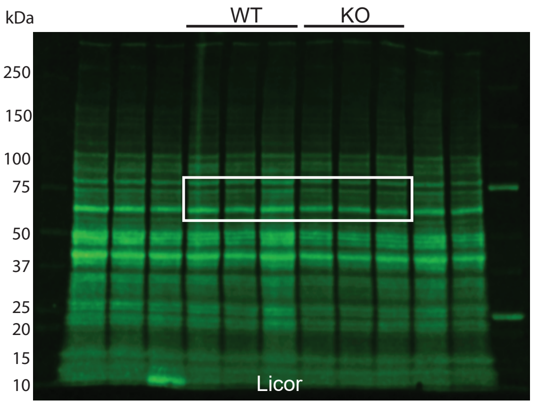

Full length blots for figure 5B

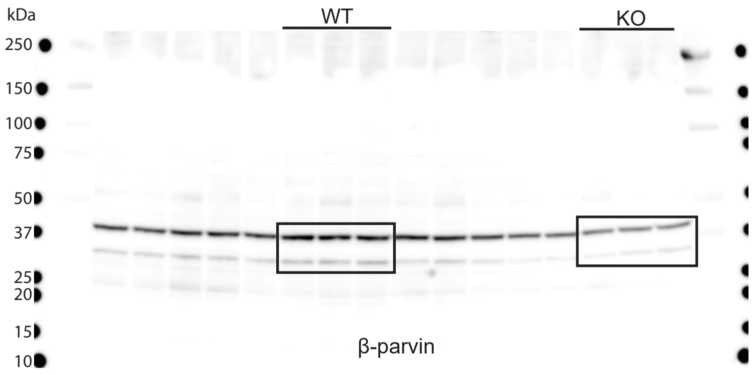

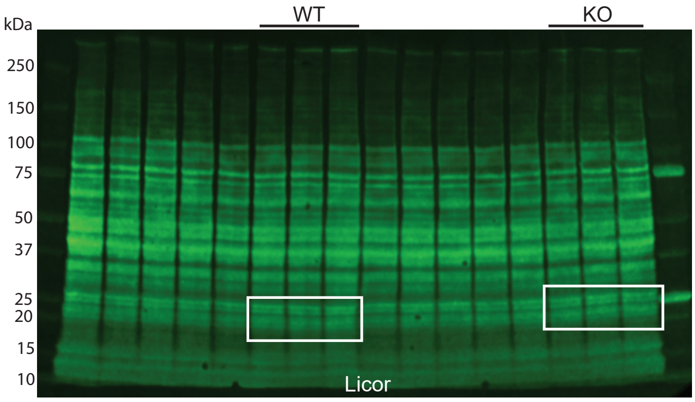

Full length blots for figure 5C

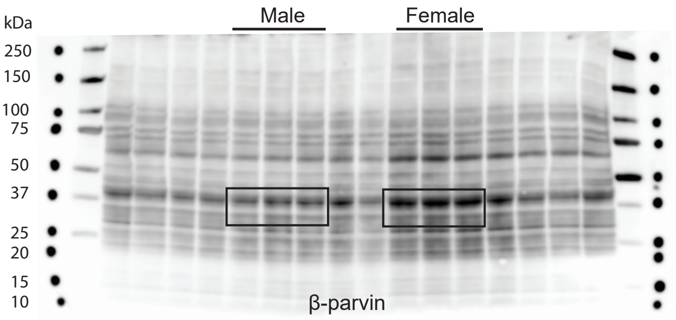

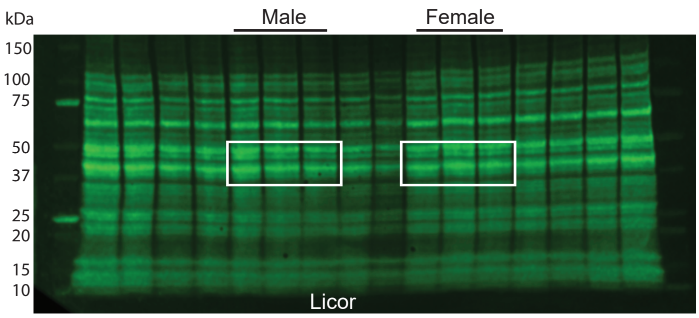

Full length blots for figure 5D

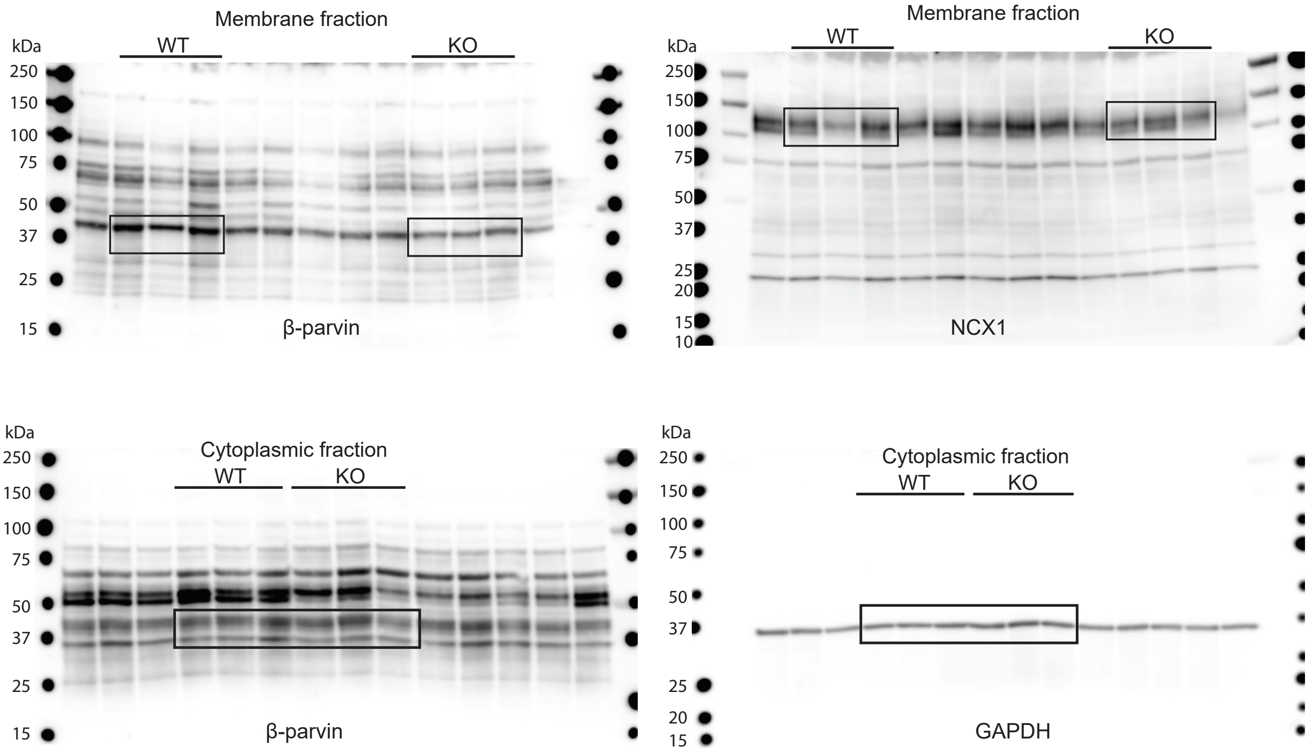

Full length blots for figure 5E

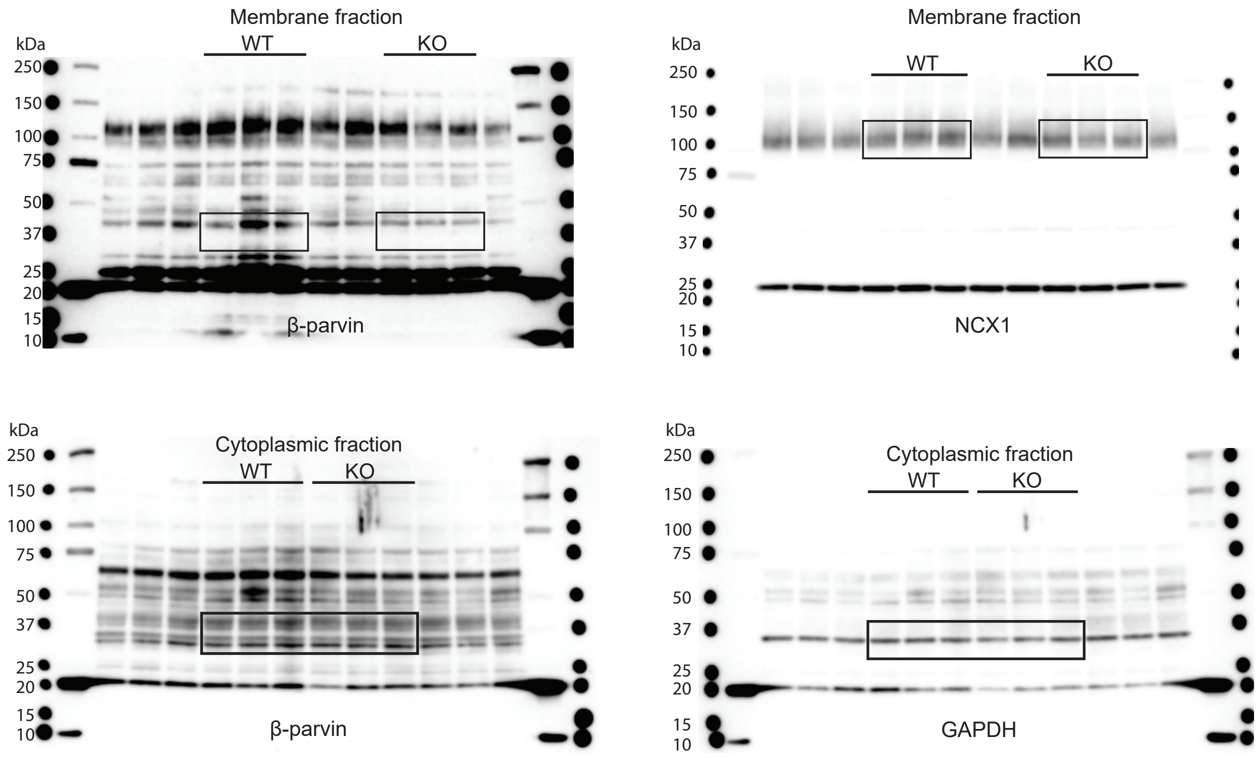

Full length blots for figure 6A

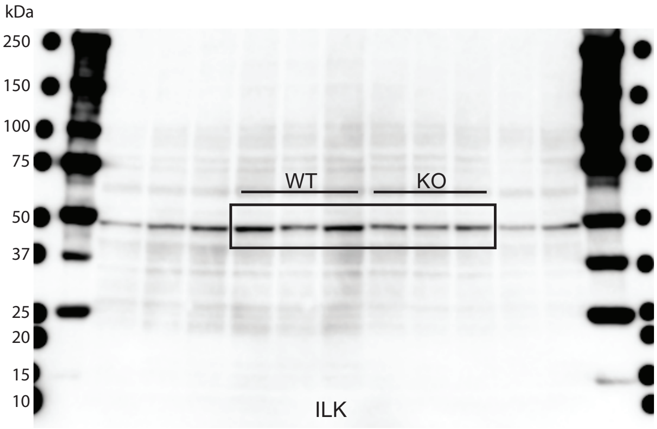

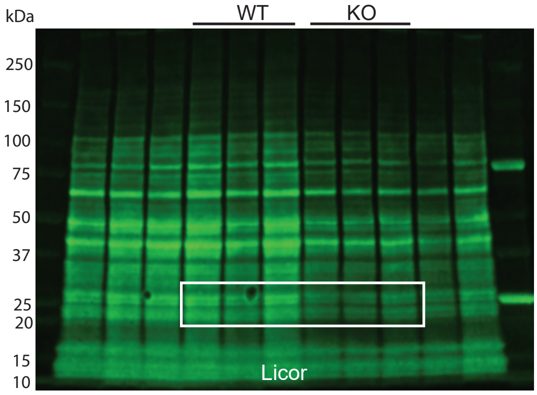

Full length blots for figure 6B

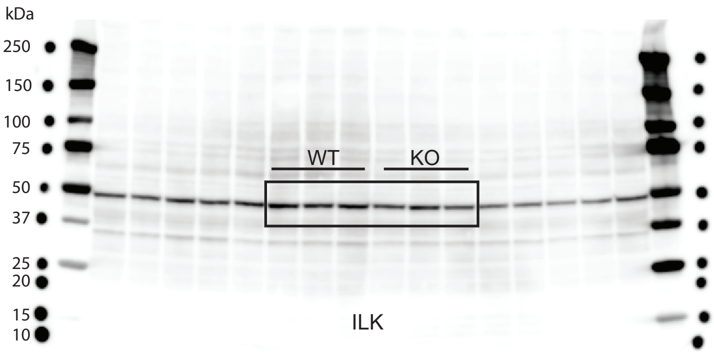

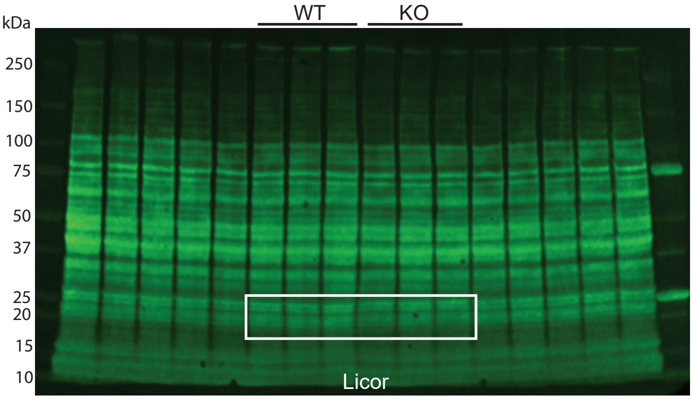

Full length blots for figure 6C

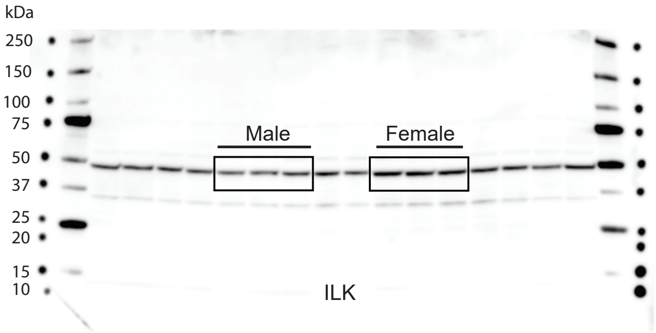

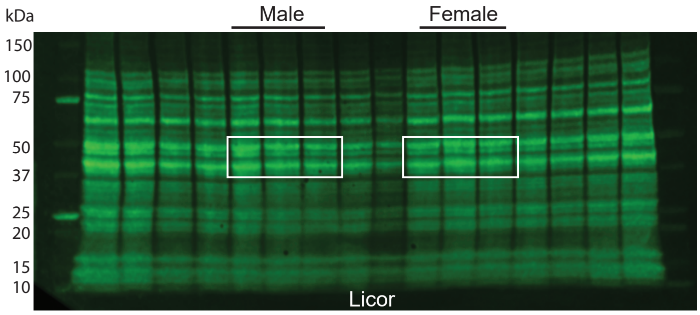

Full length blots for figure 6D

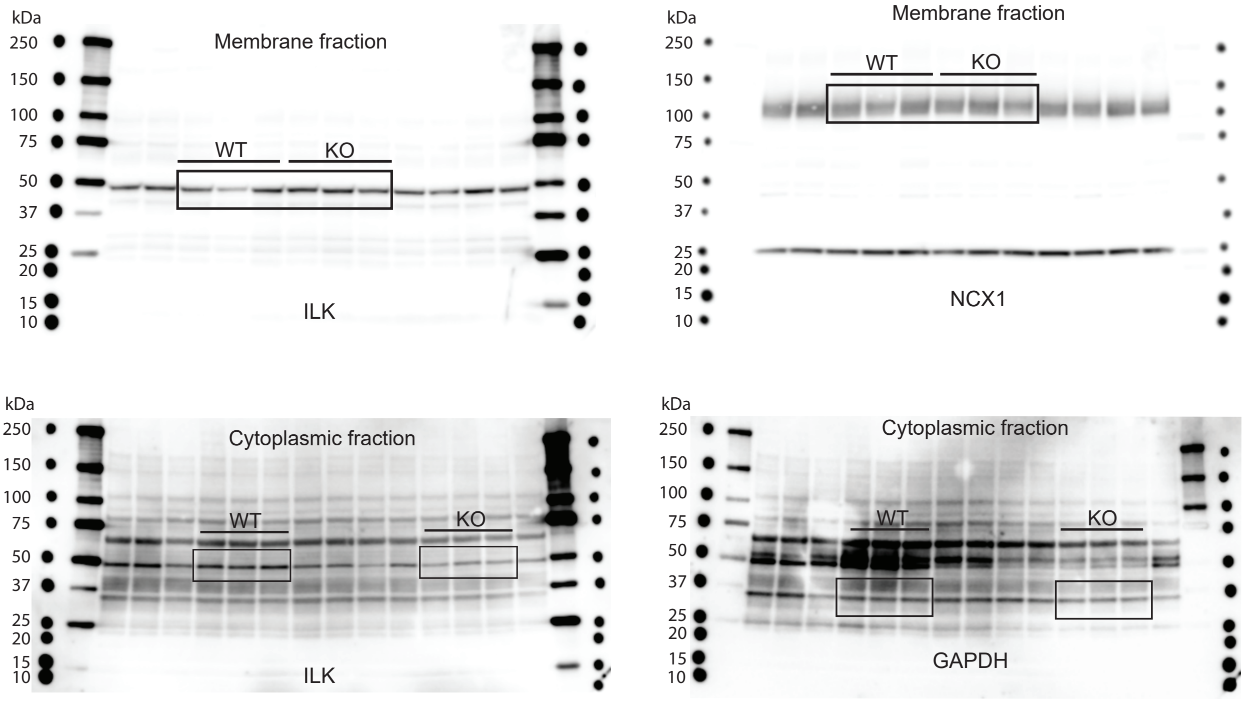

Full length blots for figure 6E

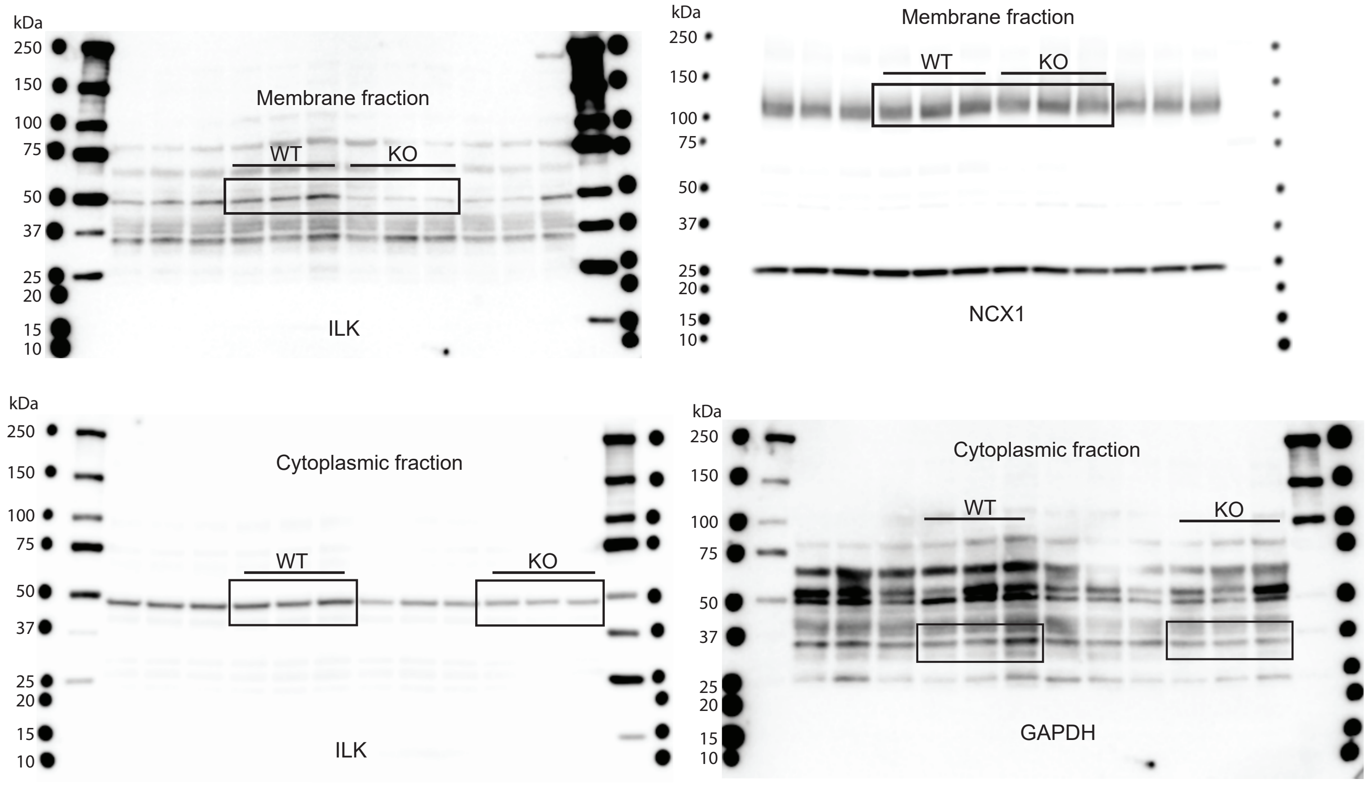

Full length blots for figure 6F

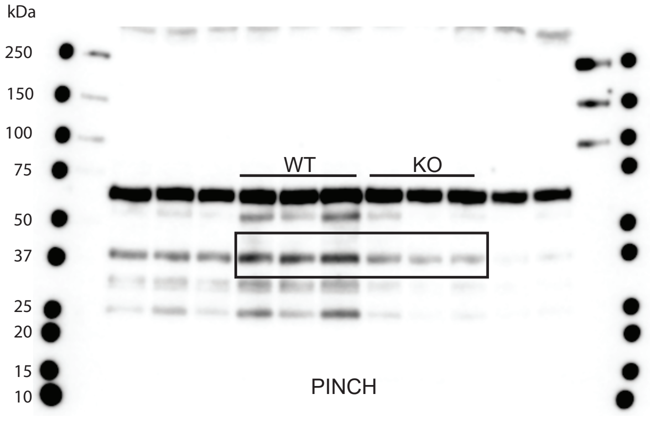

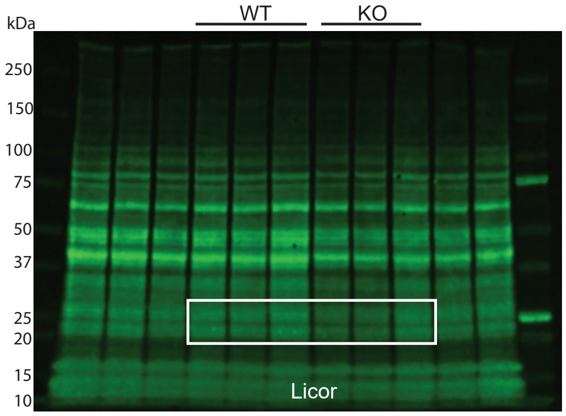

Full length blots for figure 6G

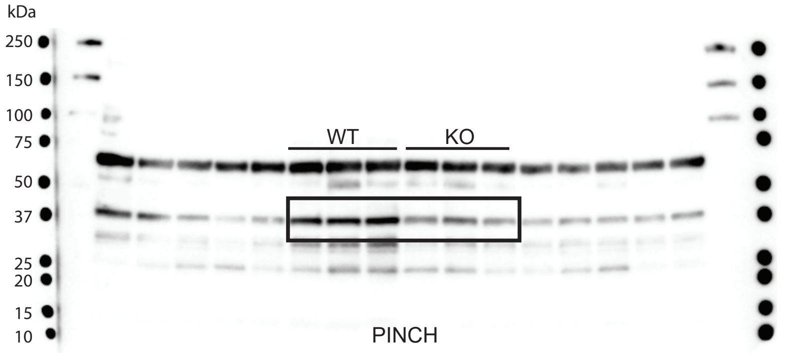

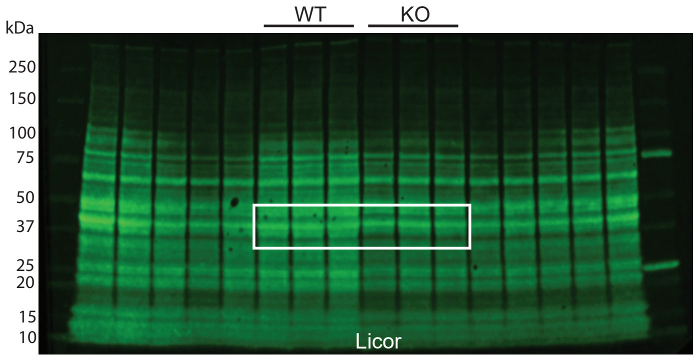

Full length blots for figure 6H

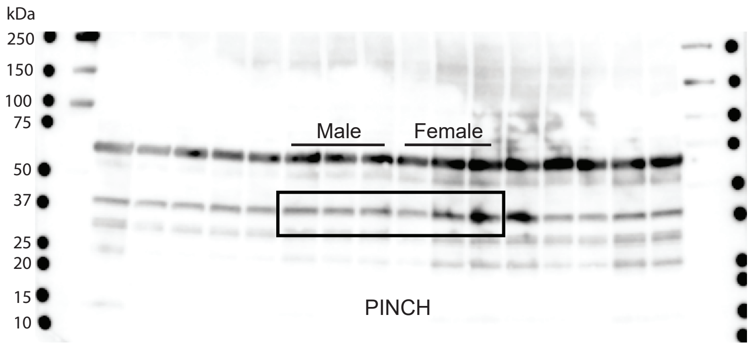

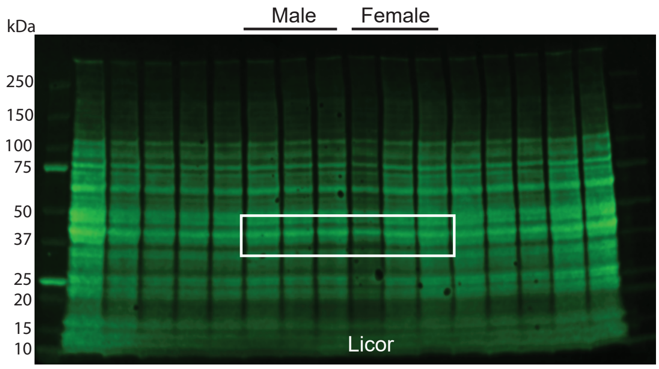

Full length blots for figure 6I

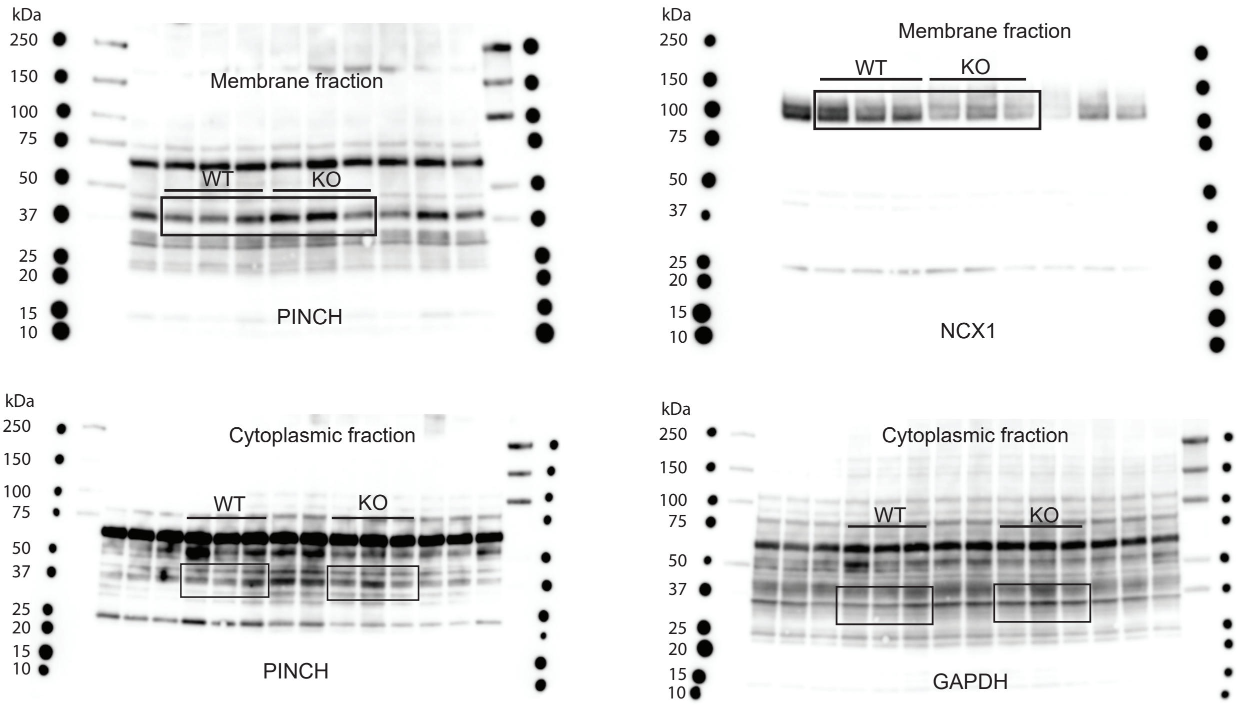

Full length blots for figure 6J

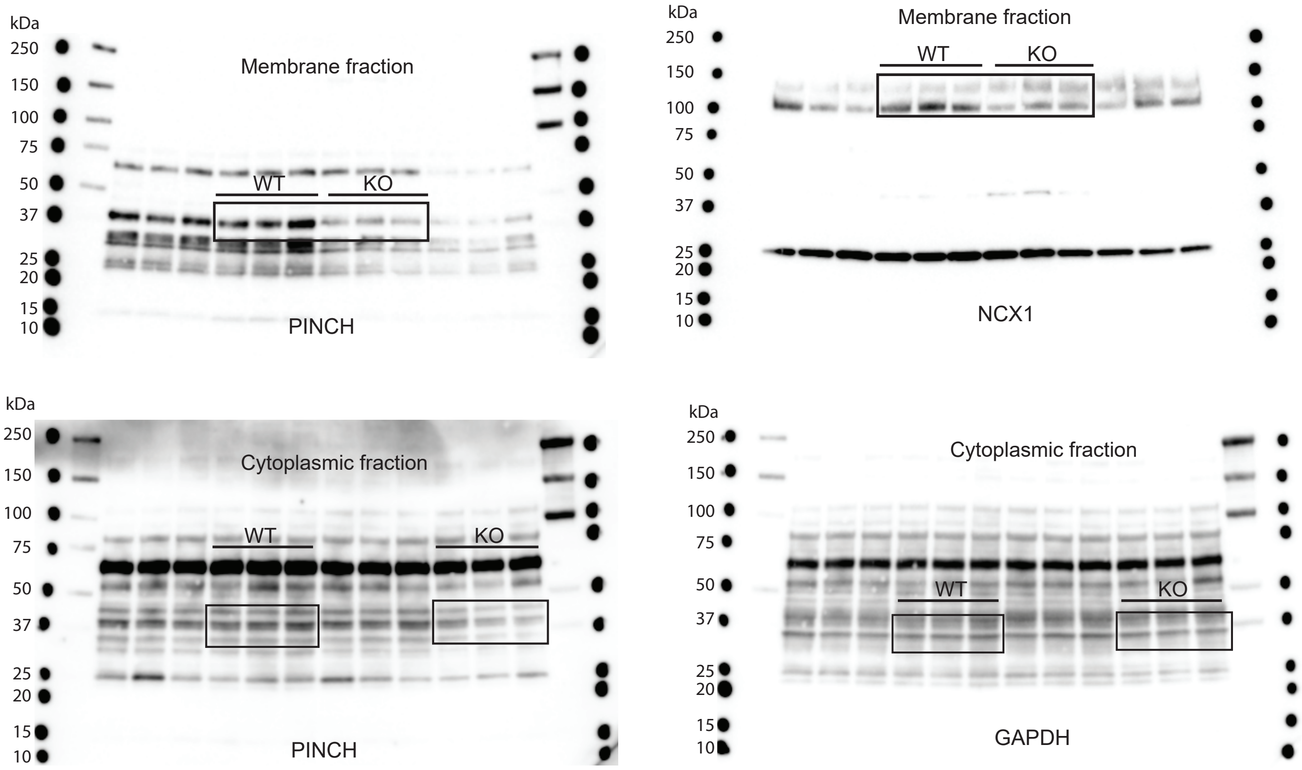

Full length blots for figure 6K

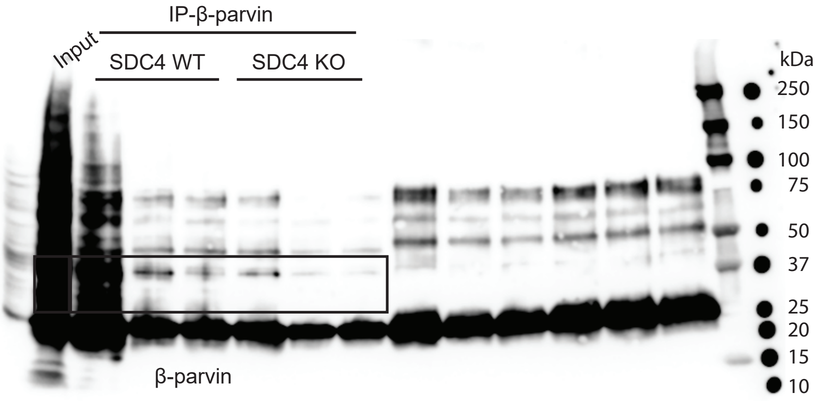

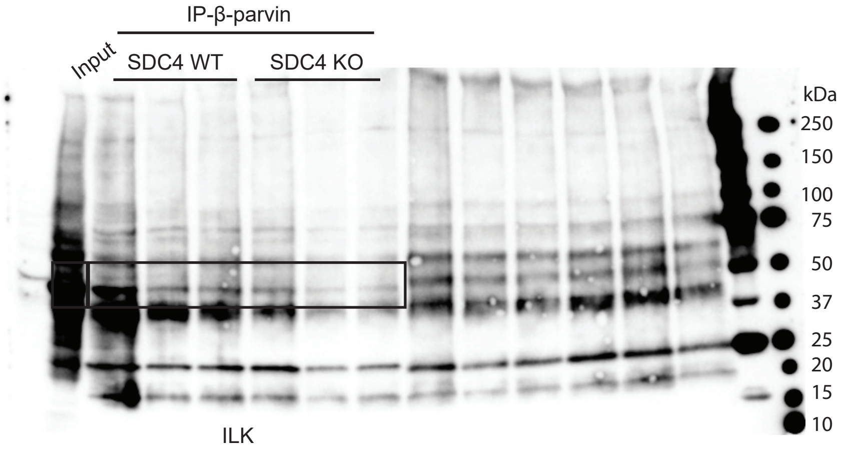

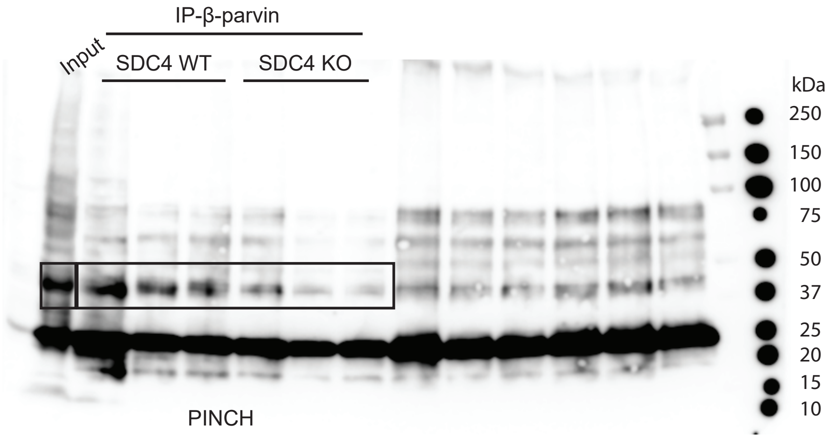

Full length blots for figure 7A

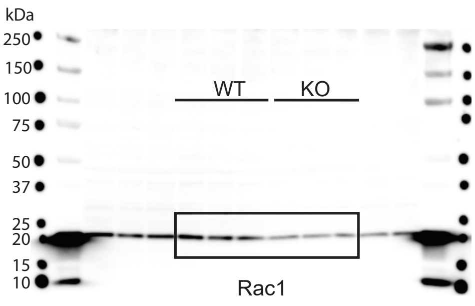

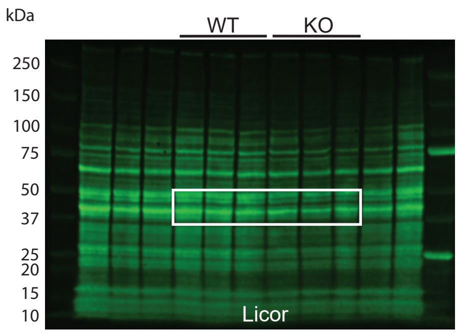

Full length blots for figure 7B

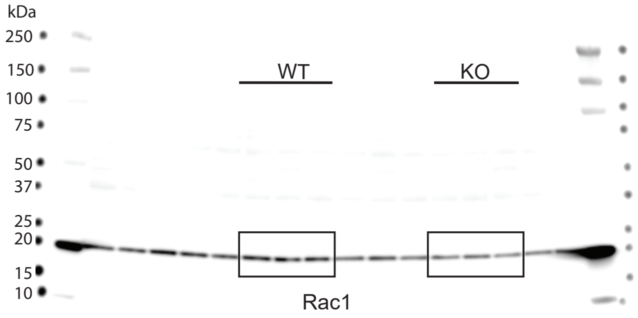

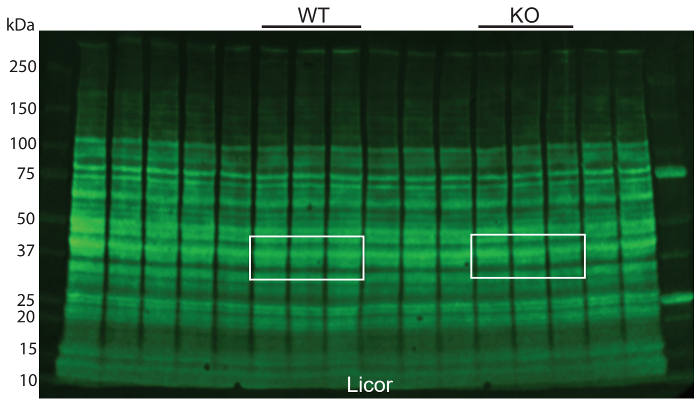

Full length blots for figure 7C

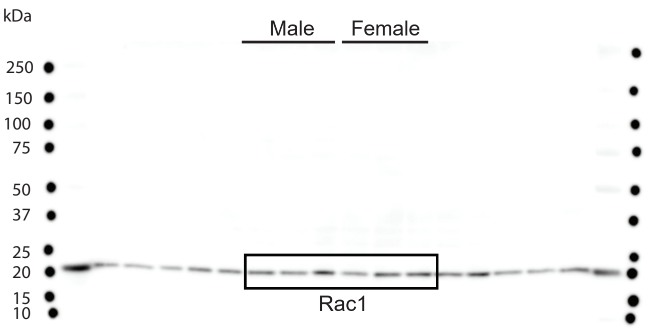

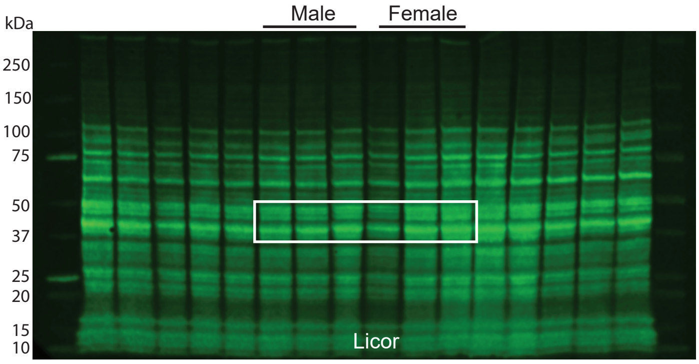

Full length blots for figure 7D

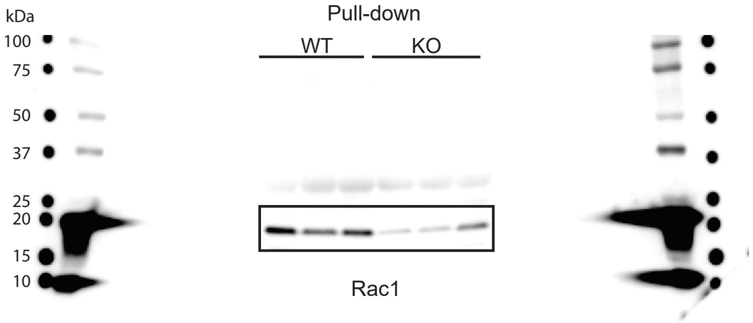

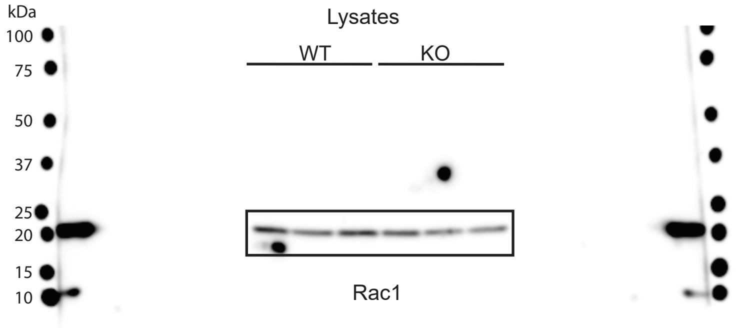

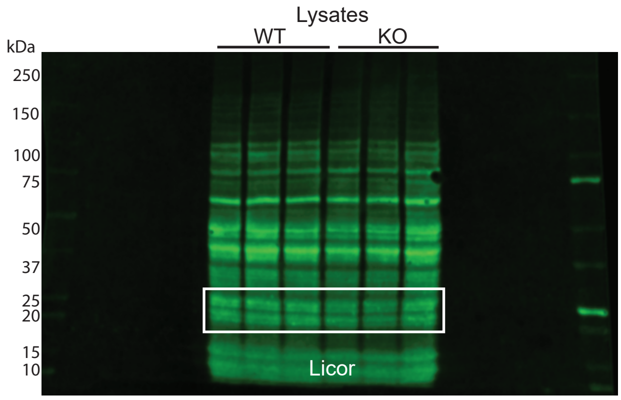

Full length blots for figure 7E

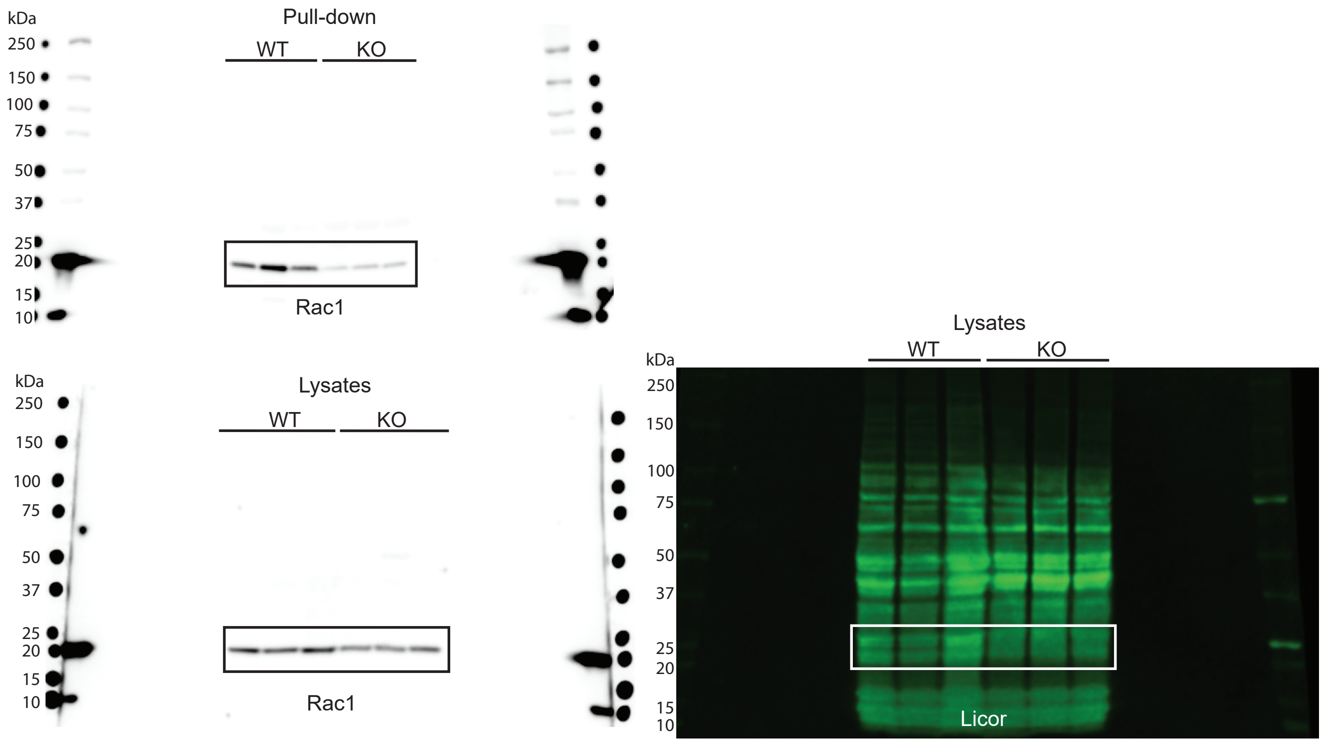

Full length blots for figure 7F

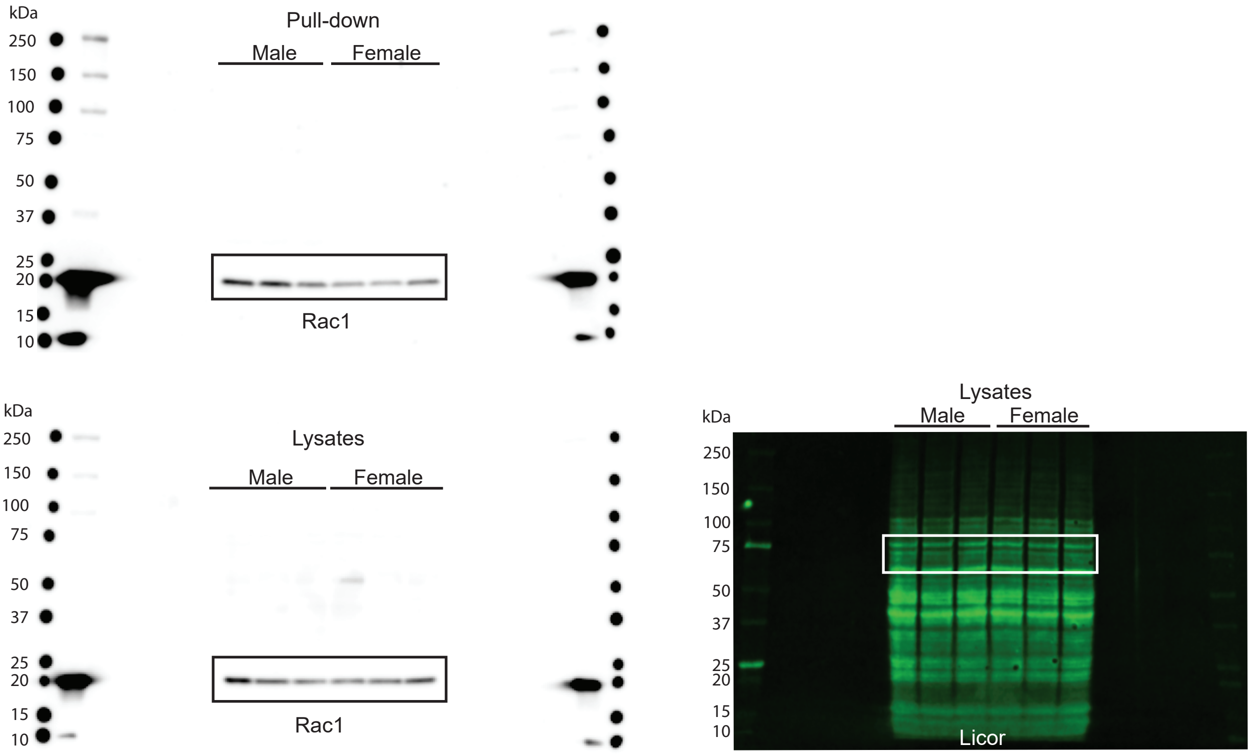

Full length blots for figure 7G

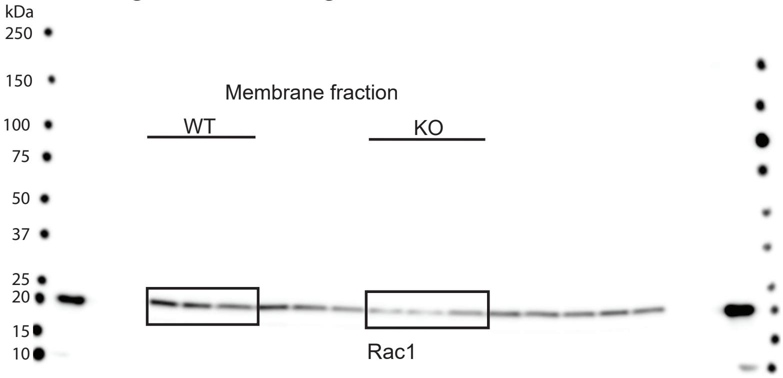

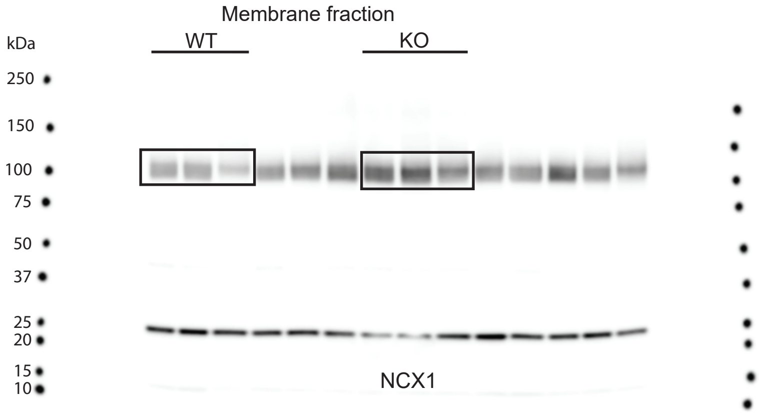

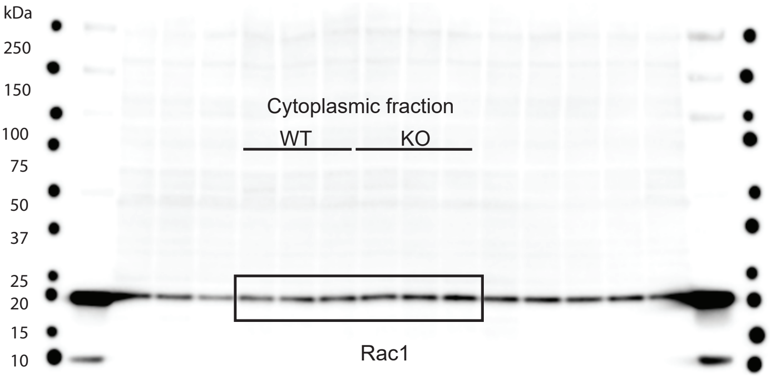

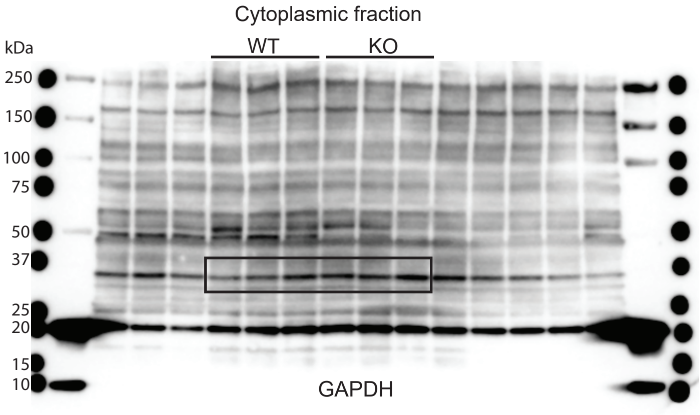

Full length blots for figure 7H

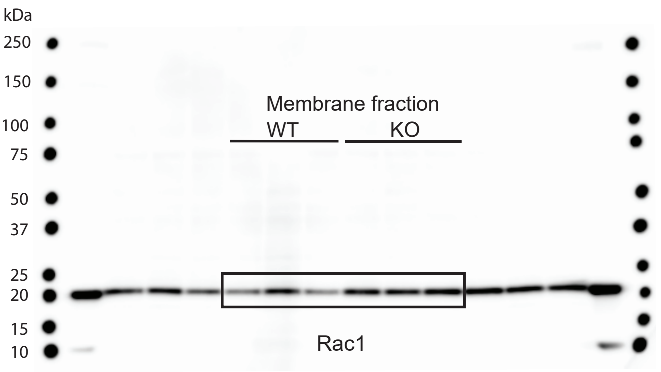

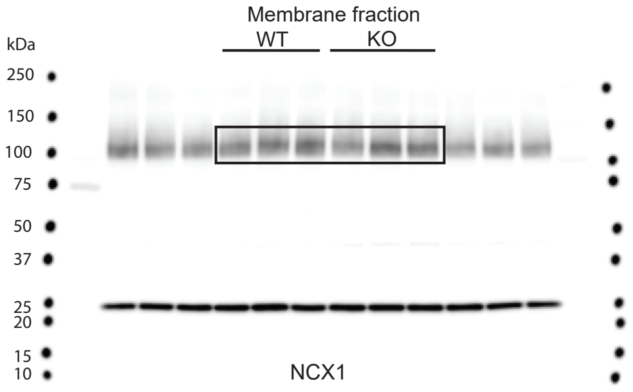

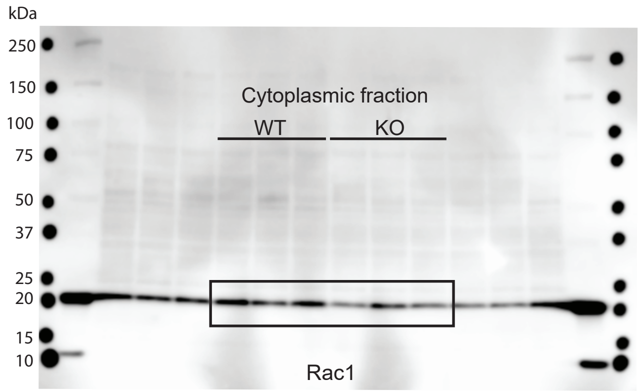

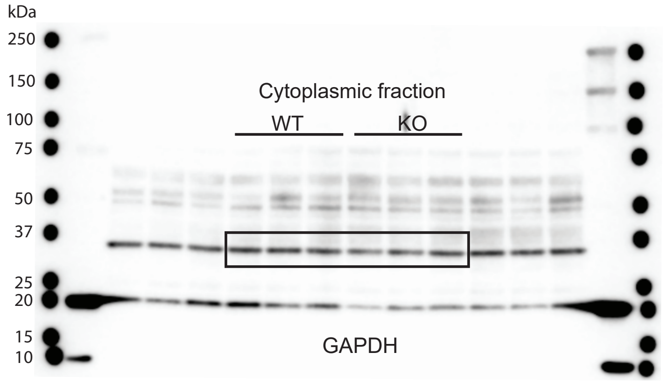

Full length blots for figure 7I

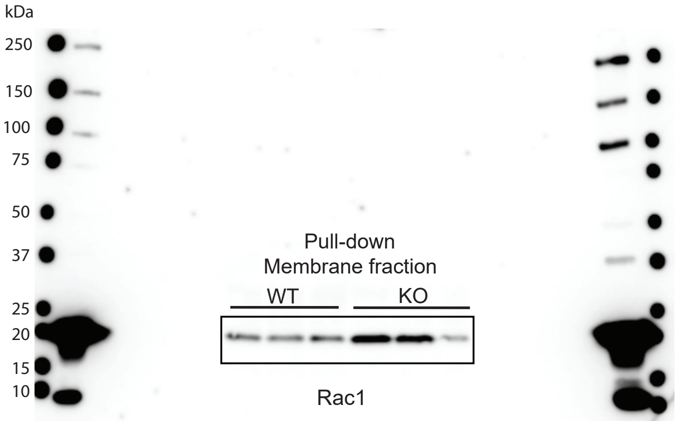

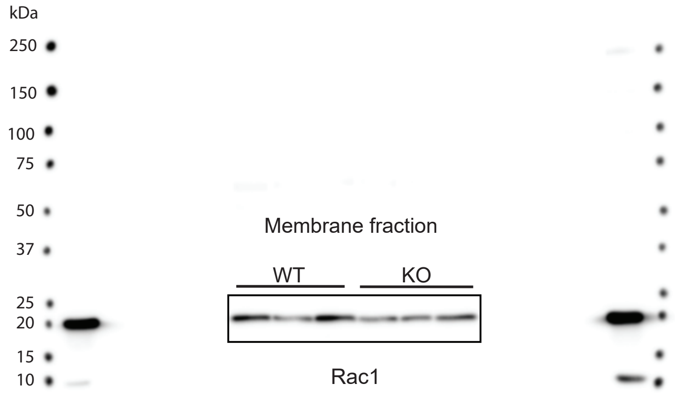

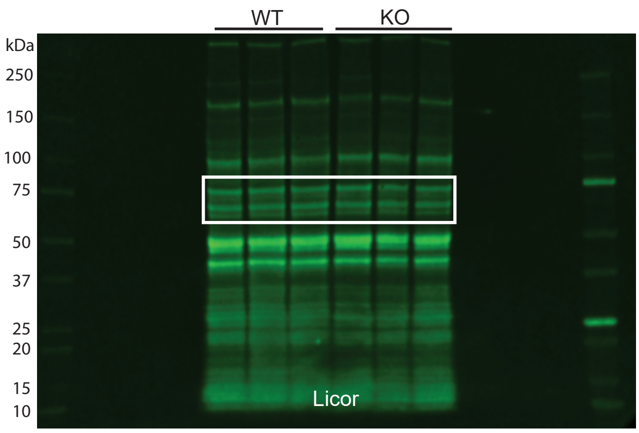

Full length blots for figure 7J

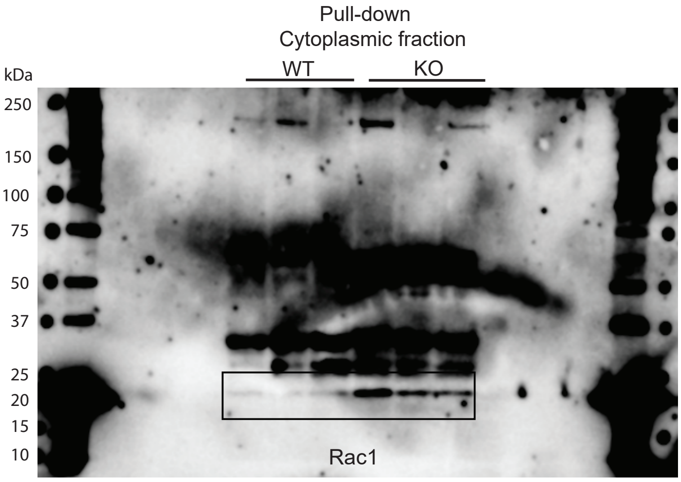

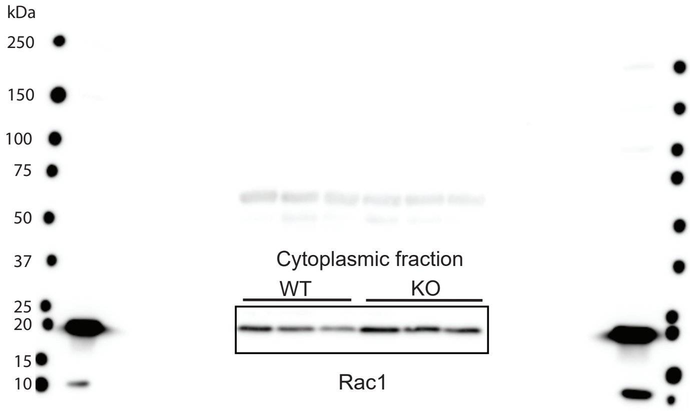

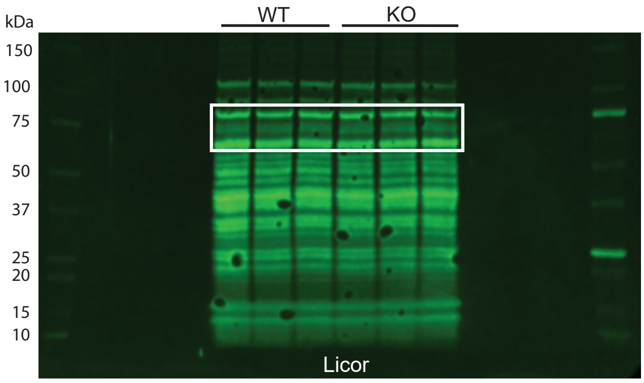

Full length blots for figure 7K

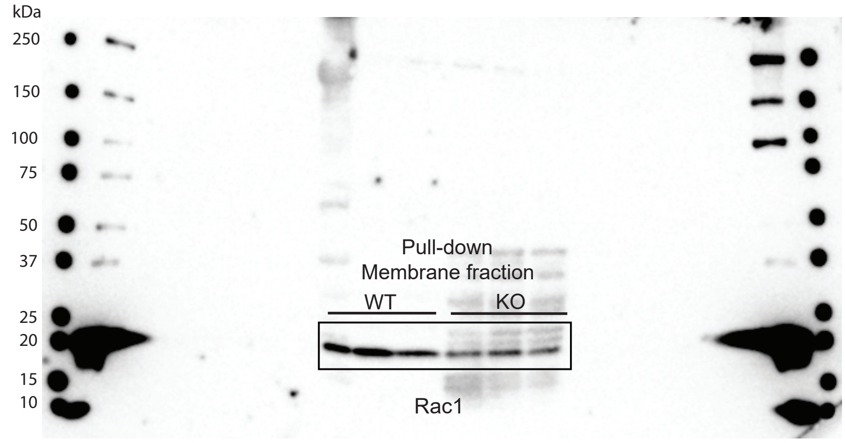

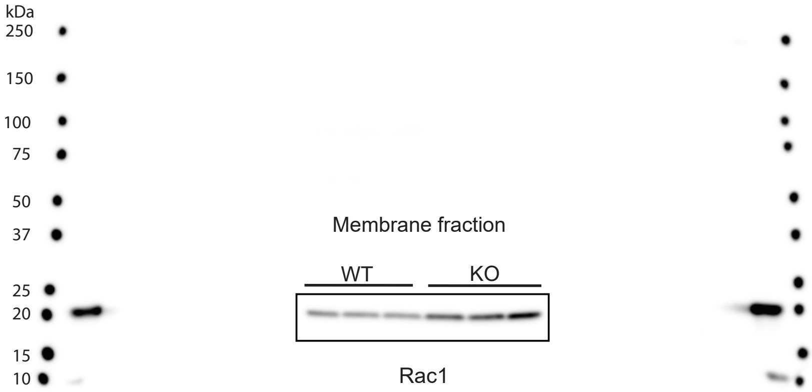

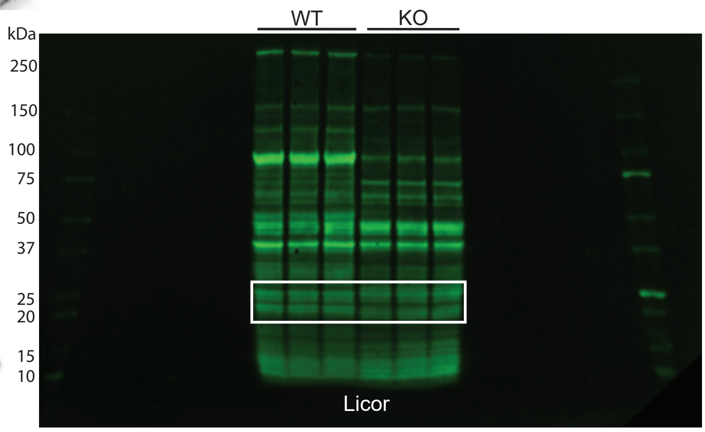

Full length blots for figure 7L

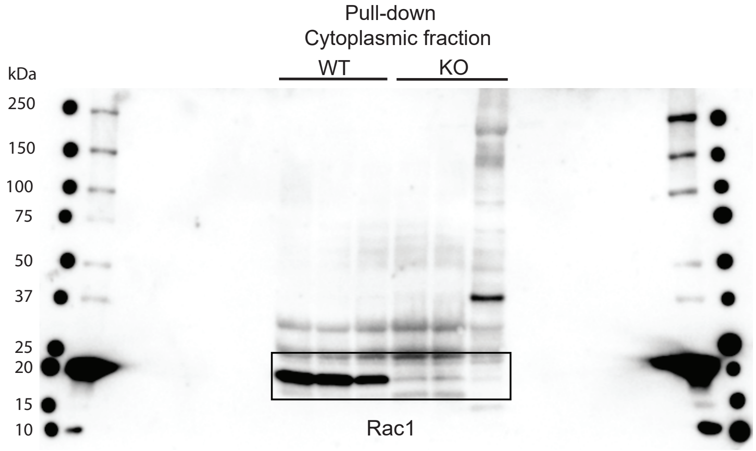

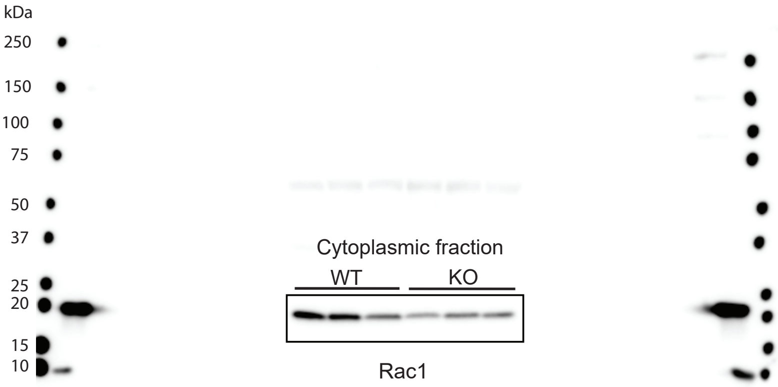

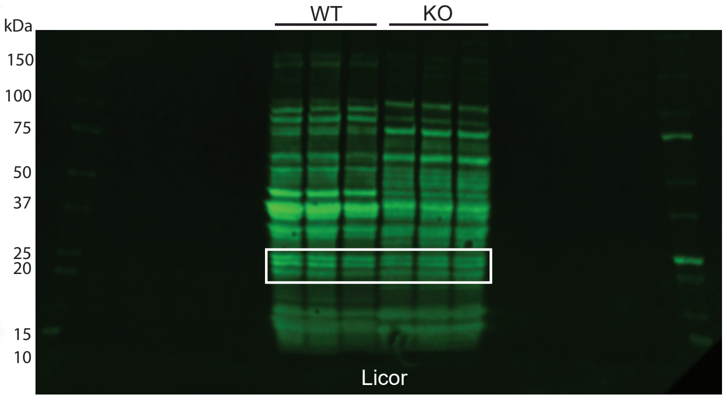

Full length blots for figure 8A

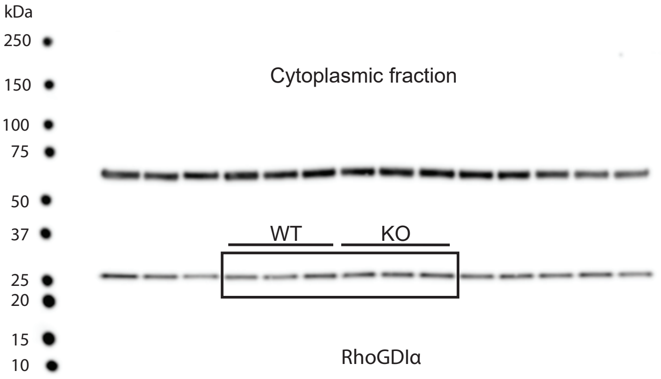

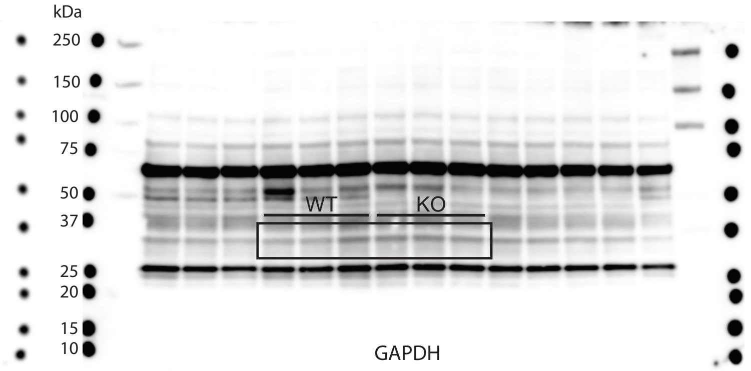

Full length blots for figure 8B

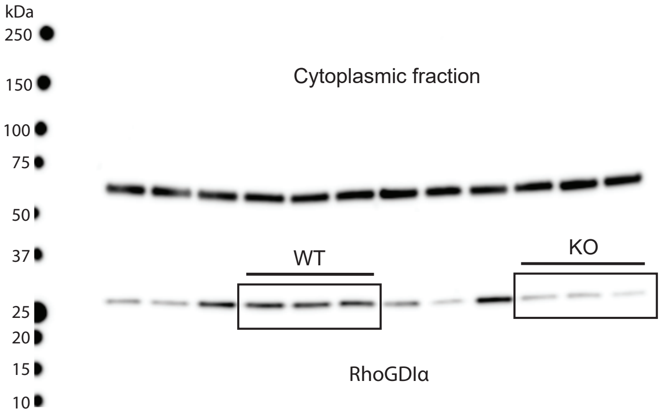

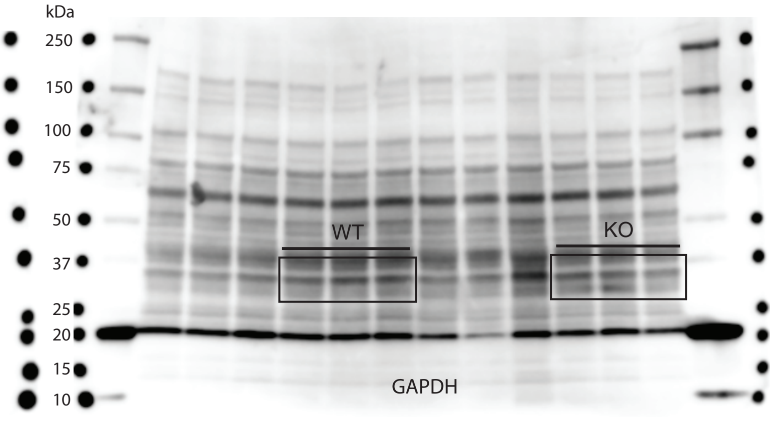

Full length blots for figure 8C

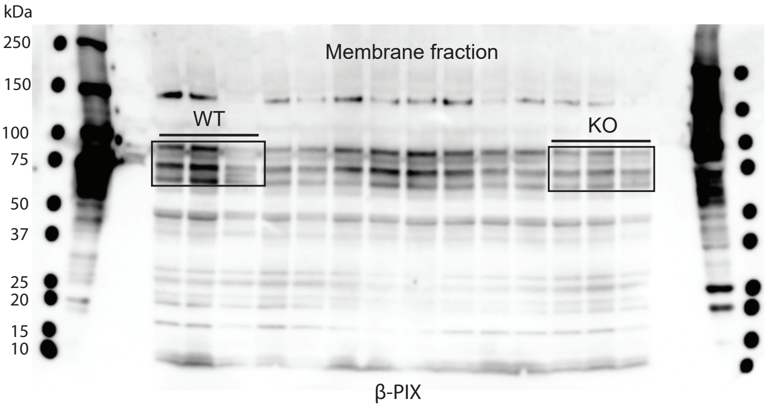

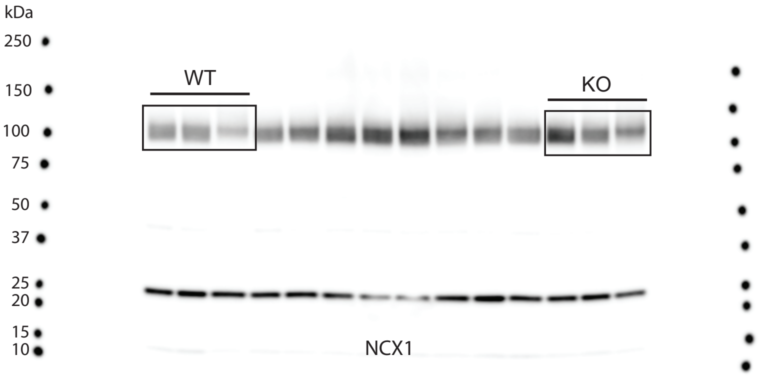

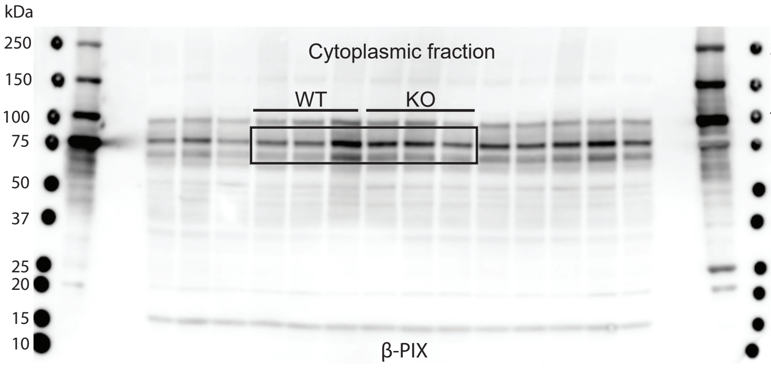

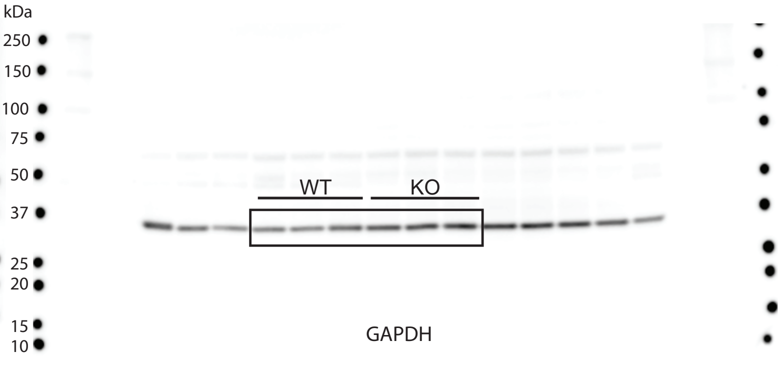

Full length blots for figure 8D

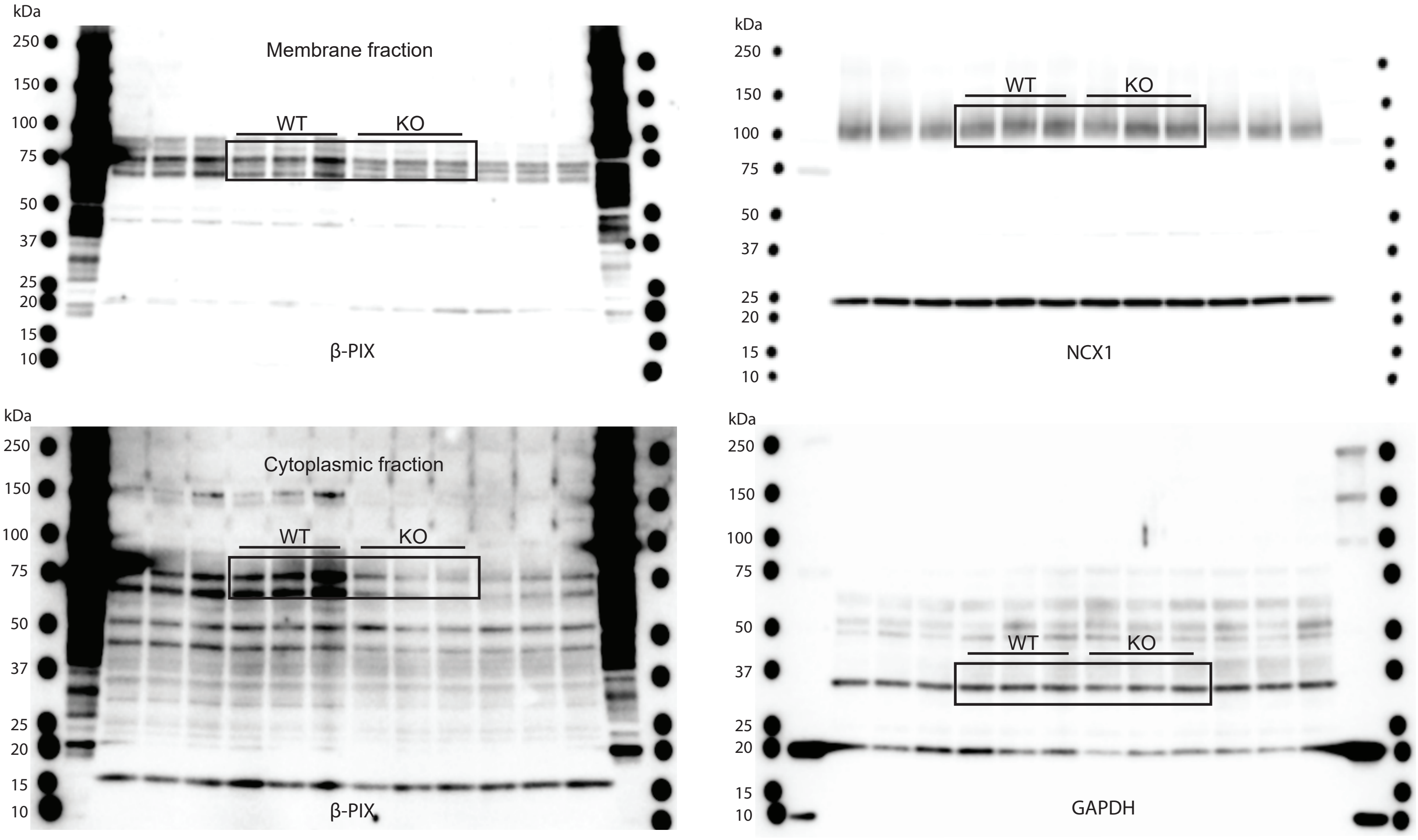

Full length blots for figure 8E

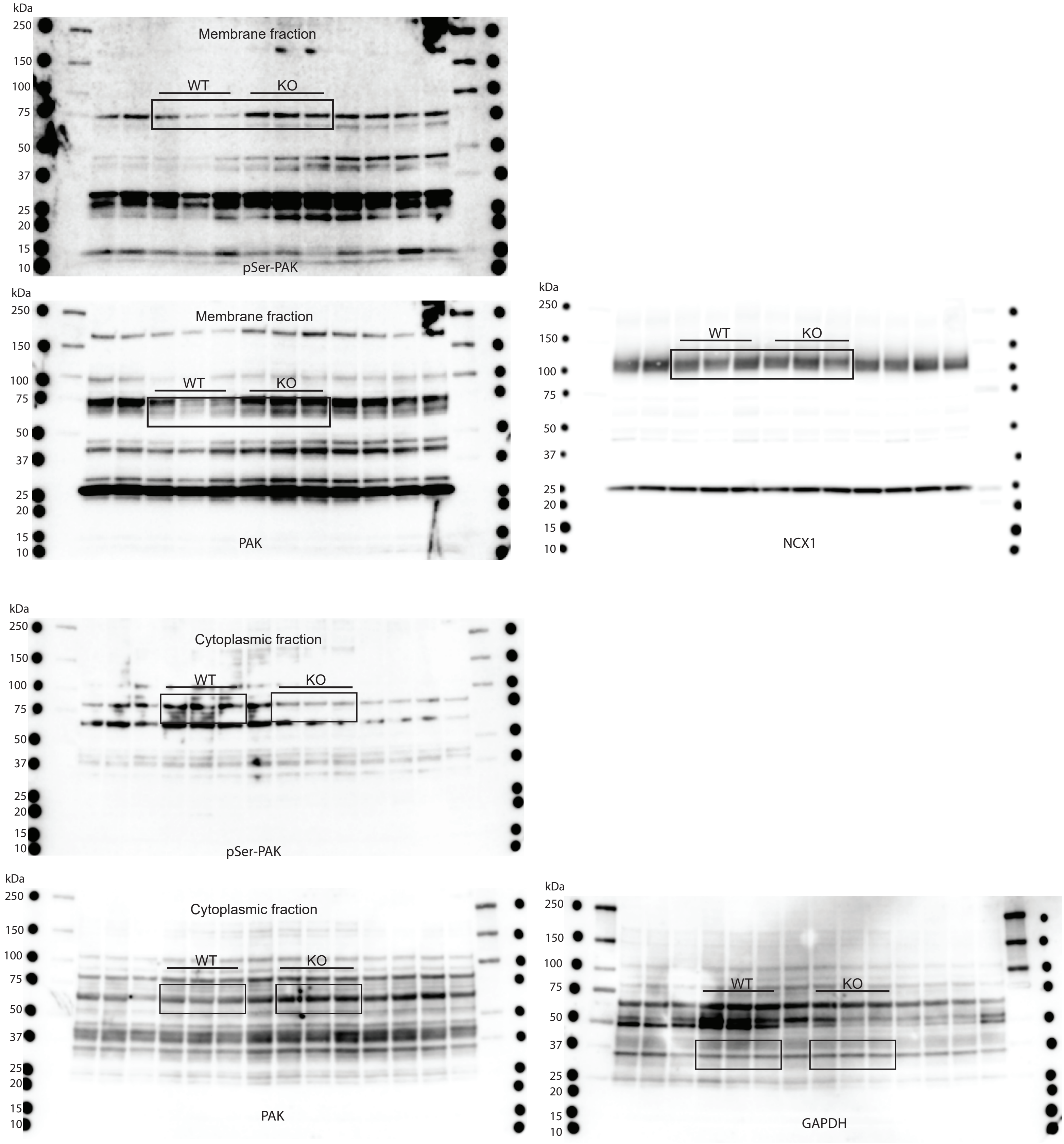

Full length blots for figure 8F

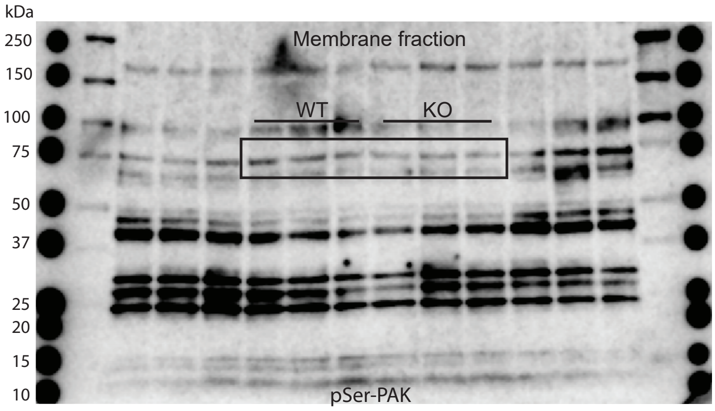

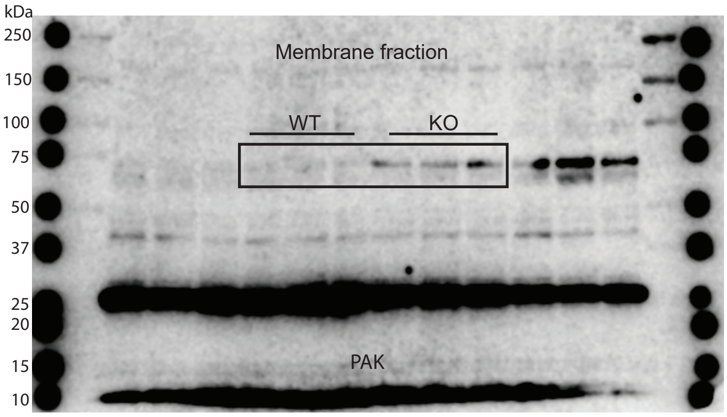

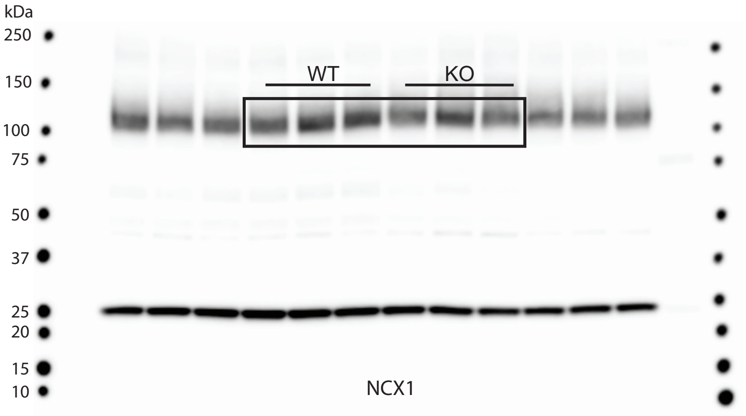

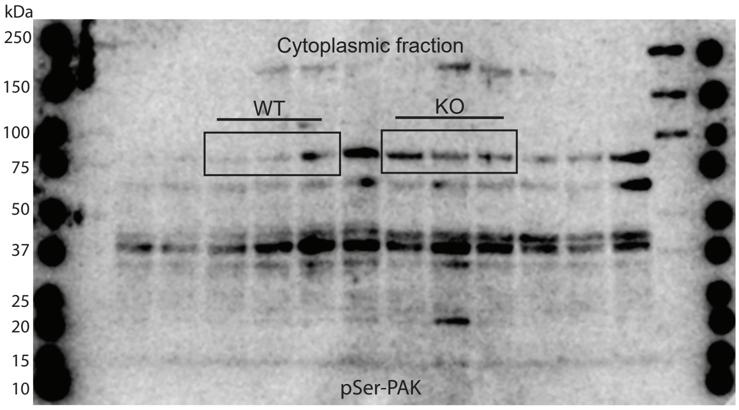

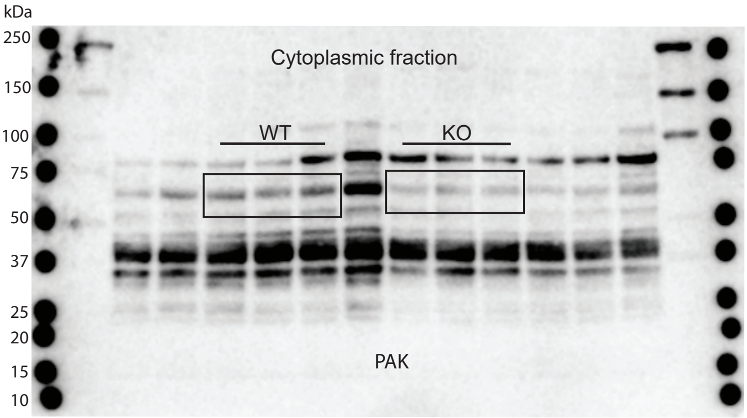

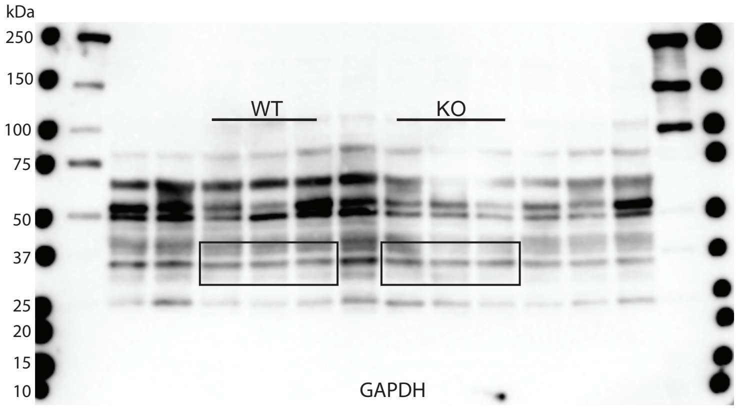

Full length blots for supplementary figure 1A

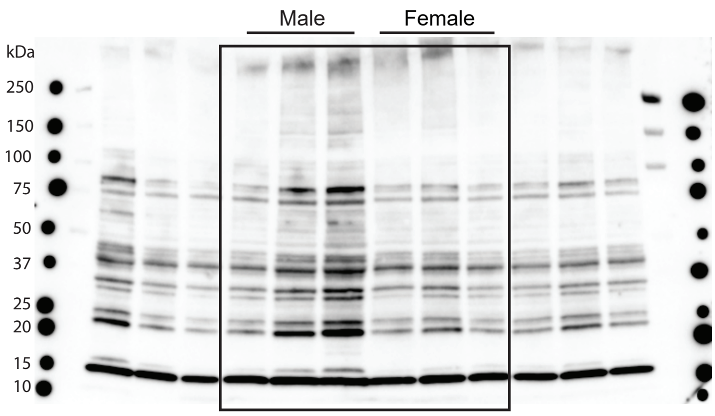

SDC4

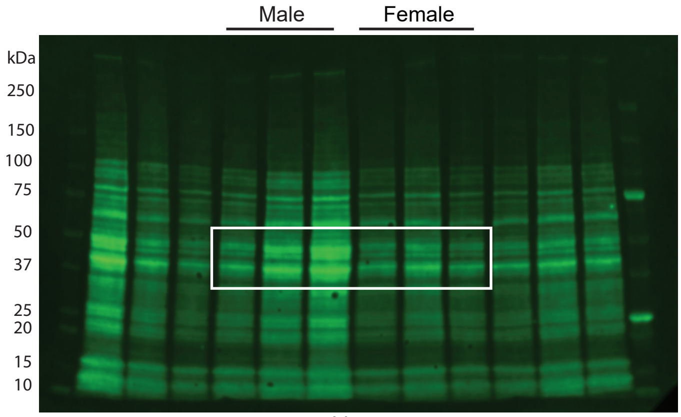

Licor

Full length blots for supplementary figure 1B

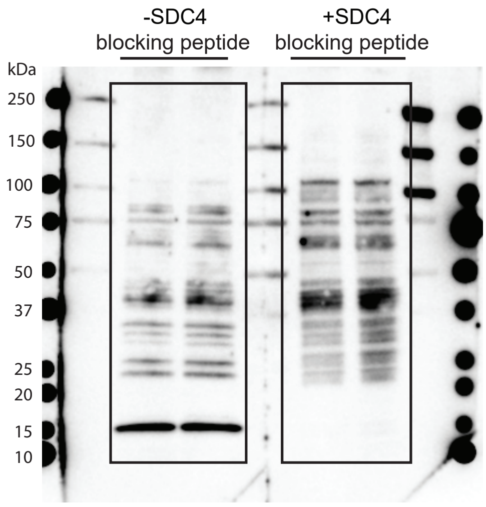

SDC4

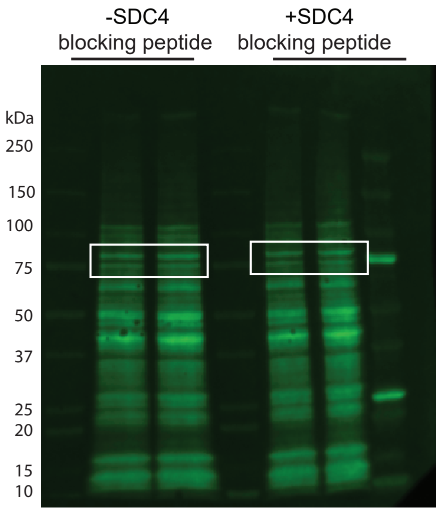

Licor

Full length blots for supplementary figure 2A

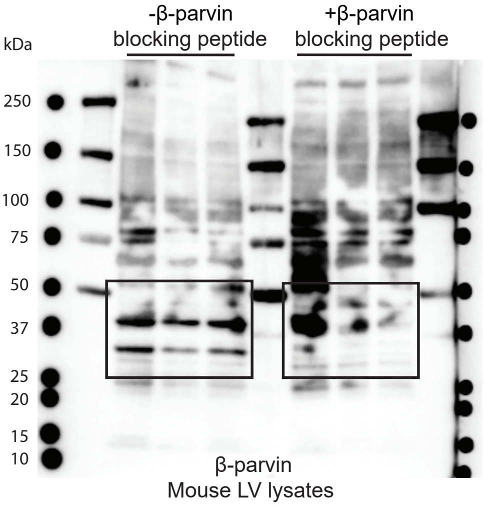

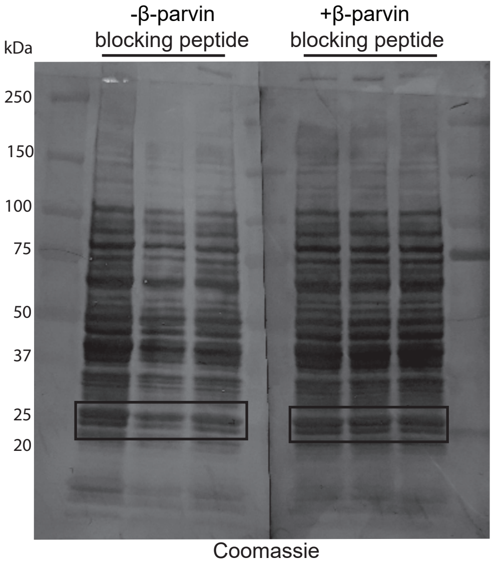

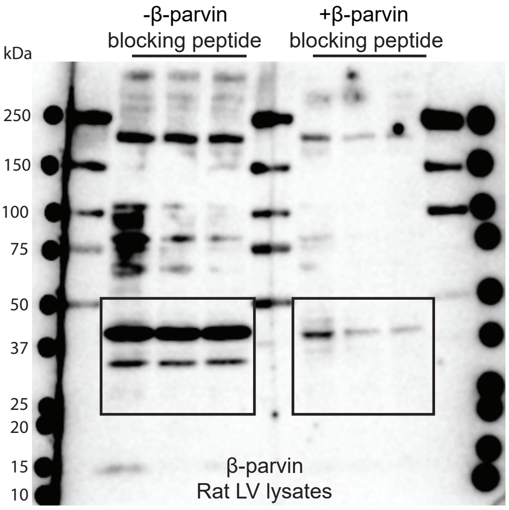

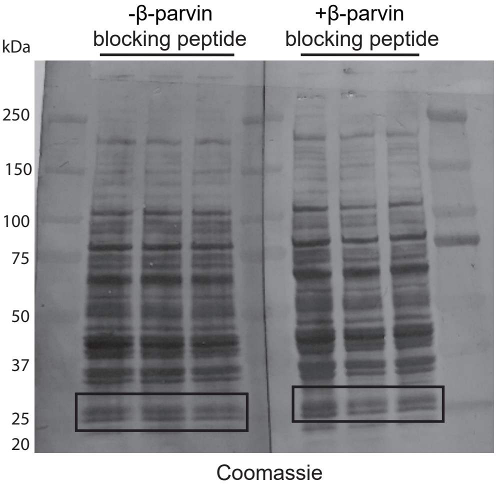

Full length blots for supplementary figure 2C

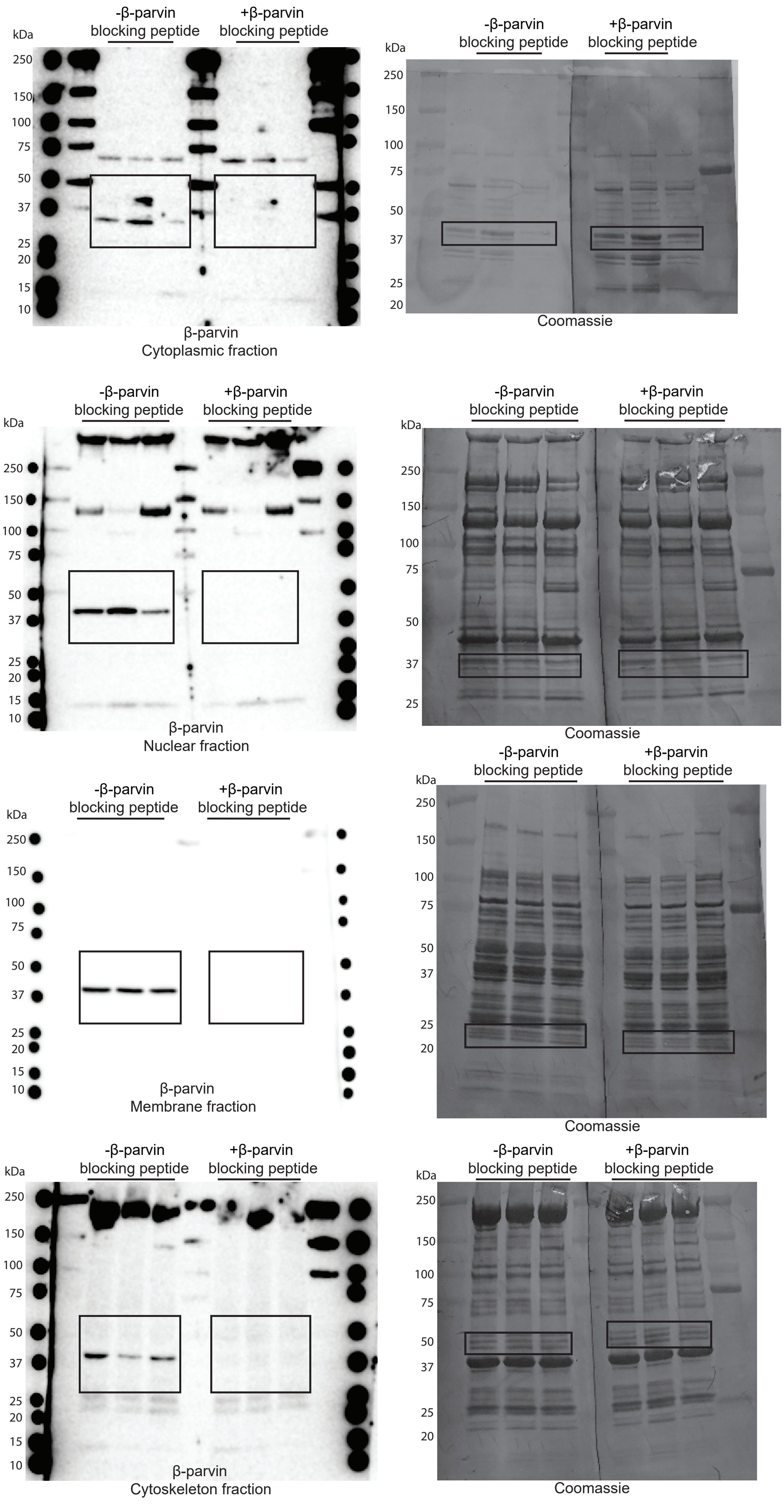

Full length blots for supplementary figure 2D

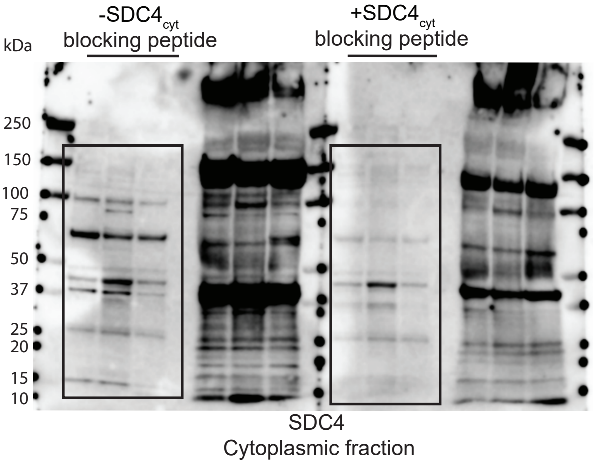

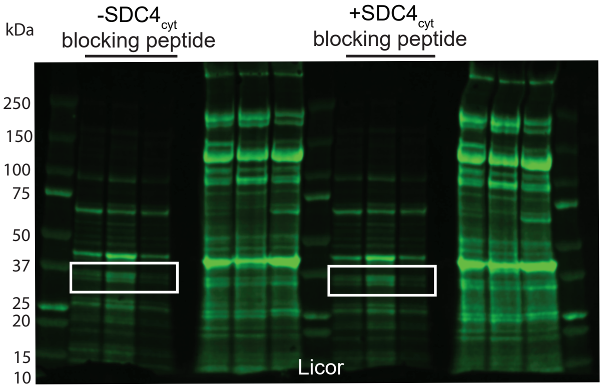

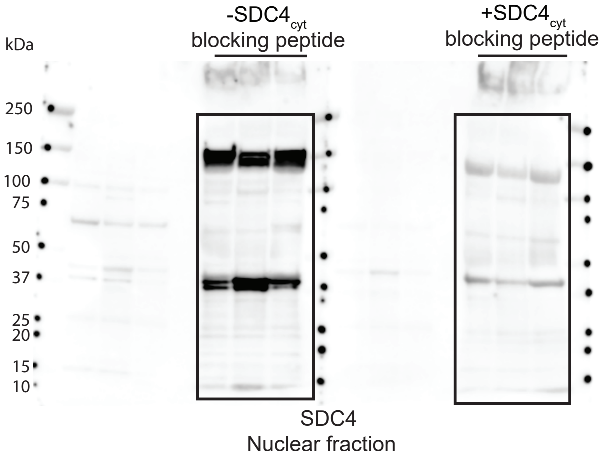

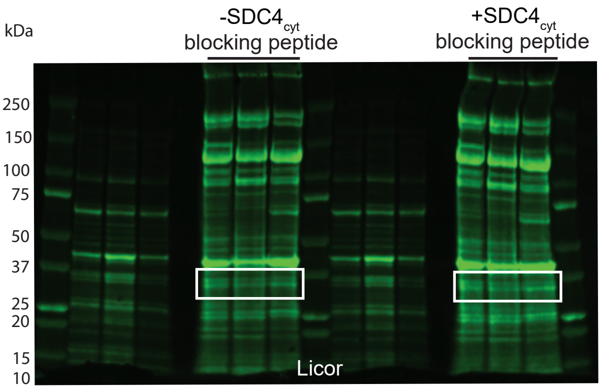

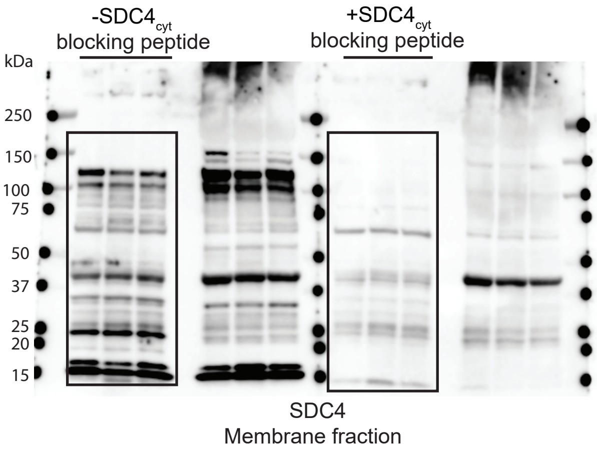

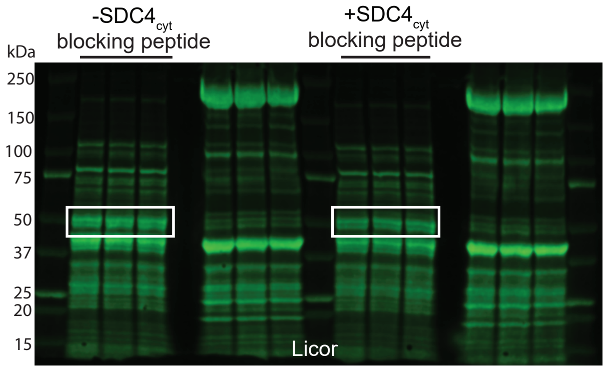

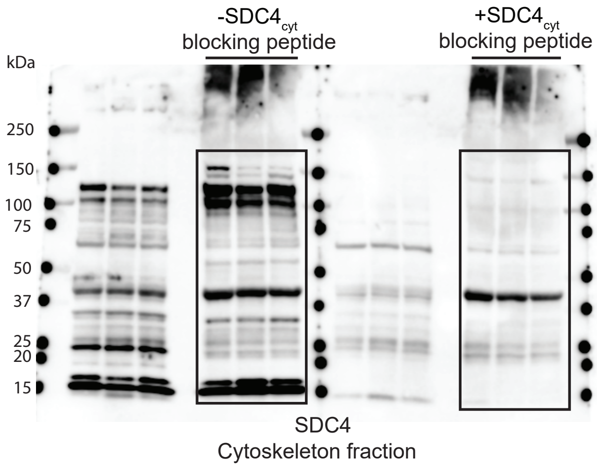

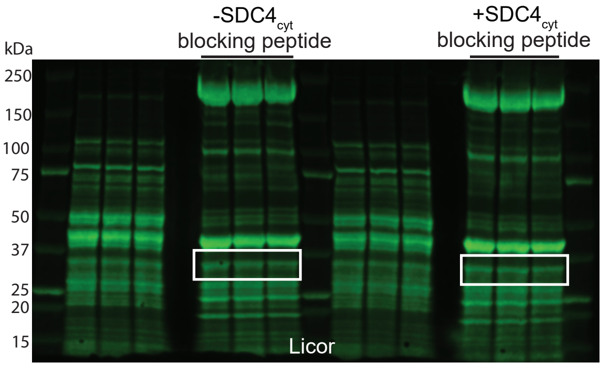

Full length blots for supplementary figure 2E

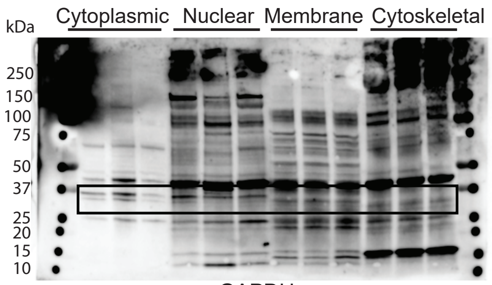

GAPDH

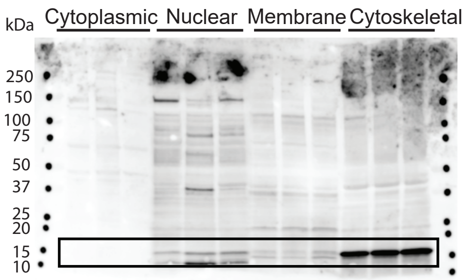

Histone H3

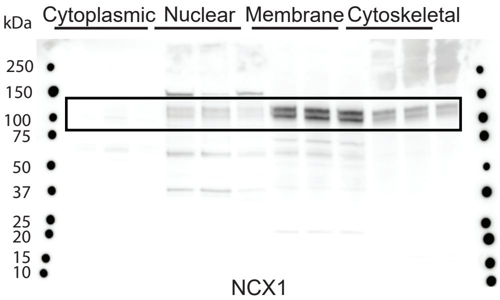

NCX1

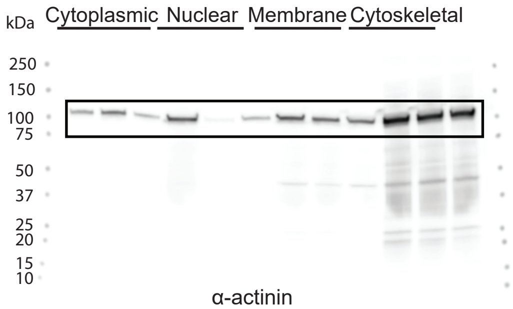

α-actinin

Full length blots for supplementary figure 4A

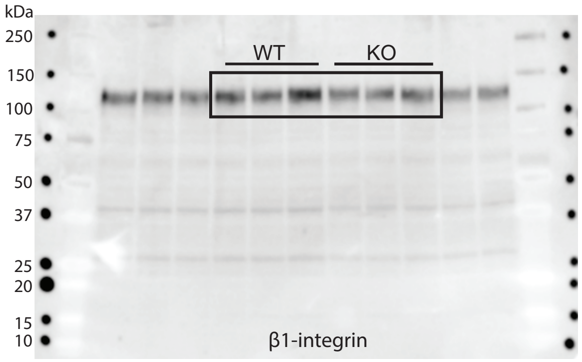

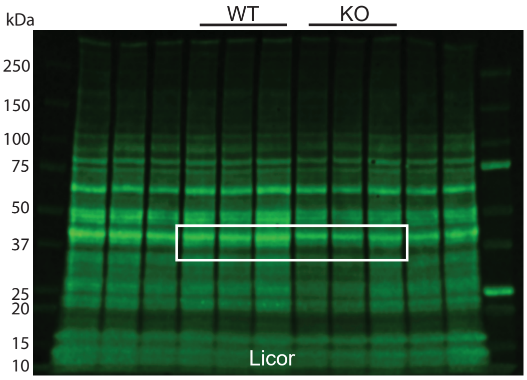

Full length blots for supplementary figure 4B

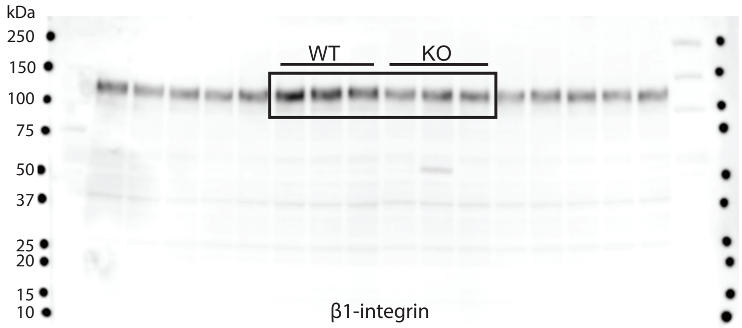

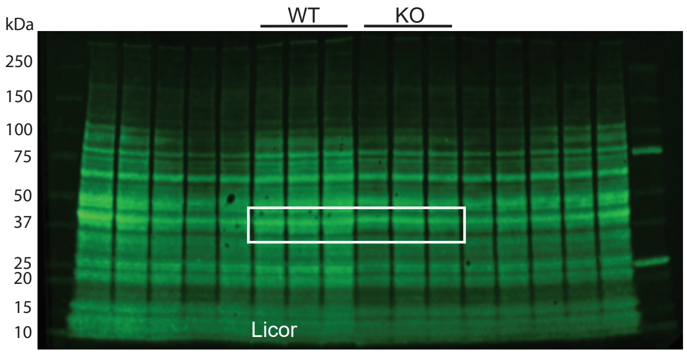

Full length blots for supplementary figure 4C

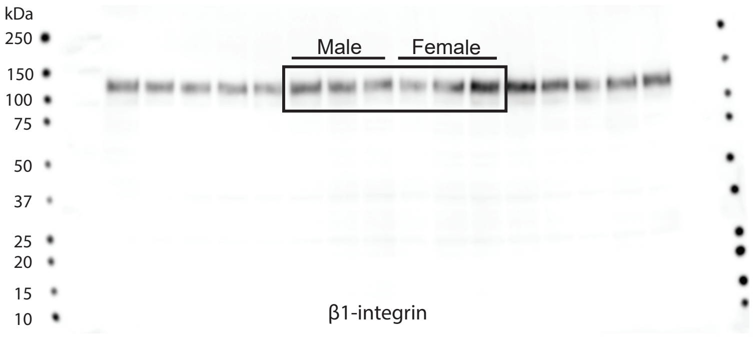

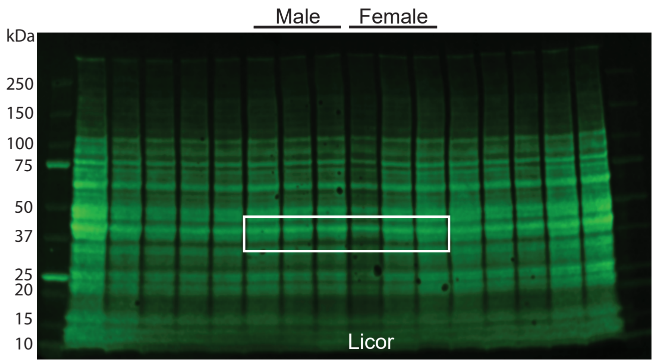

Full length blots for supplementary figure 4D

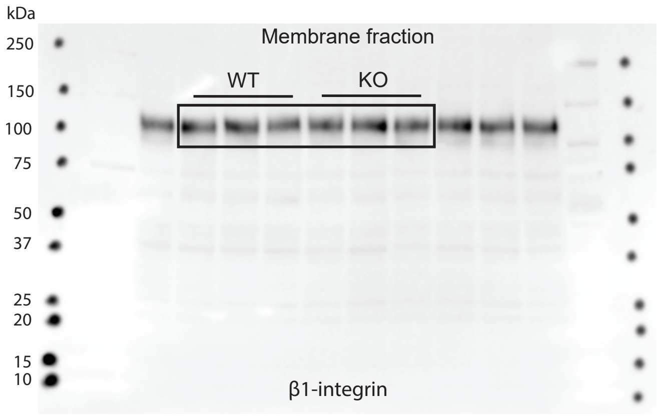

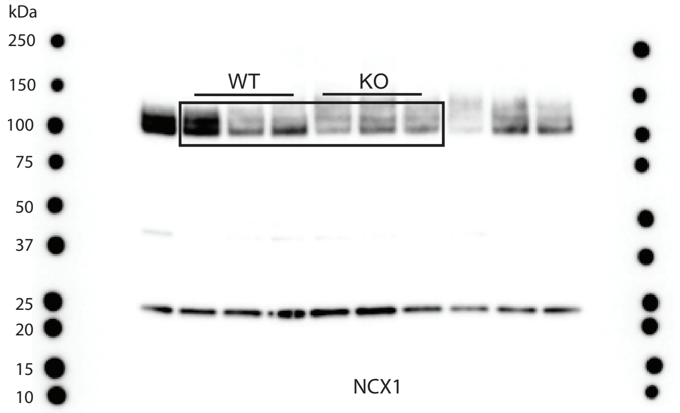

Full length blots for supplementary figure 4E

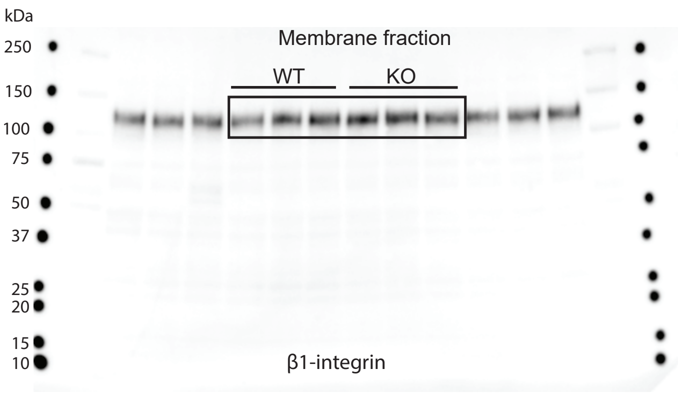

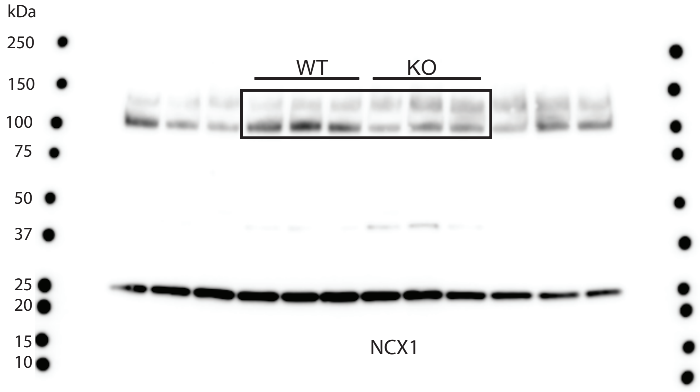

Full length blots for supplementary figure 4F

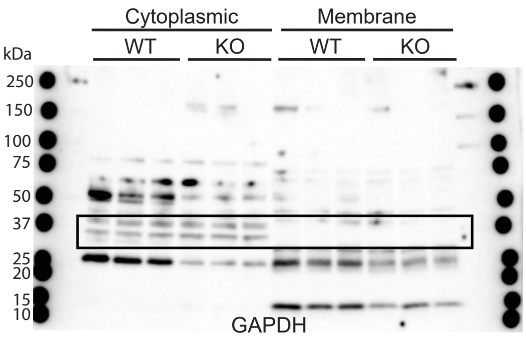

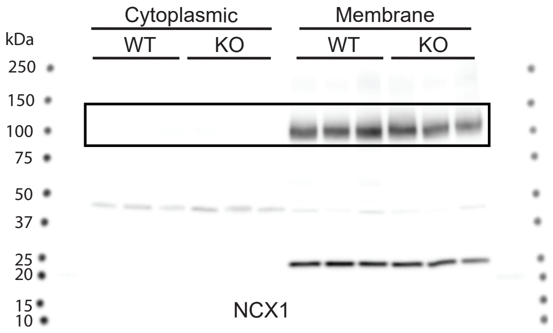

Full length blots for supplementary figure 4G

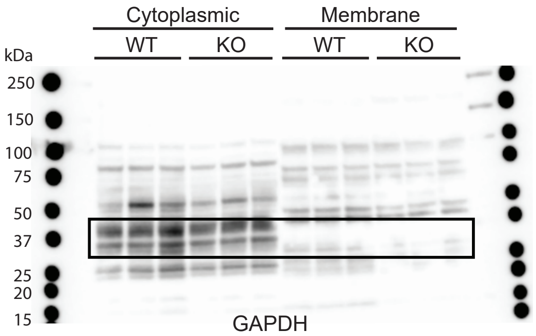

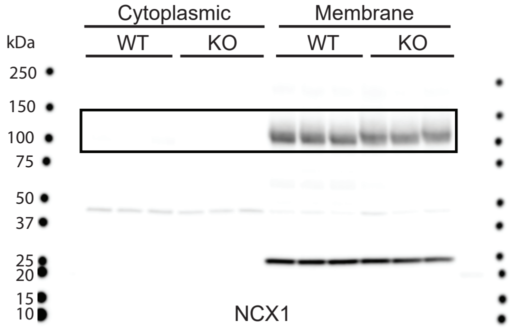

Full length blots for supplementary figure 5A

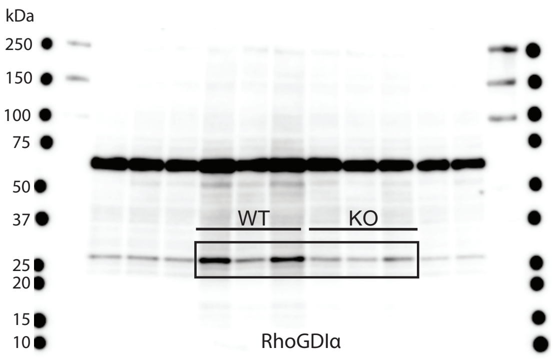

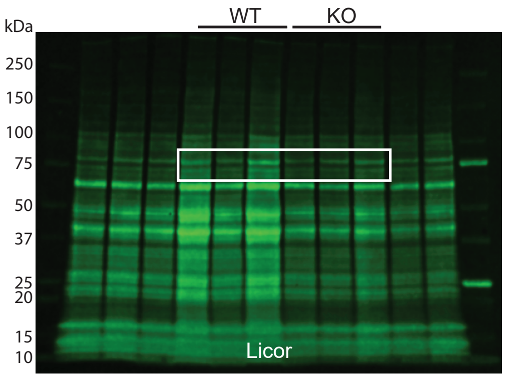

Full length blots for supplementary figure 5B

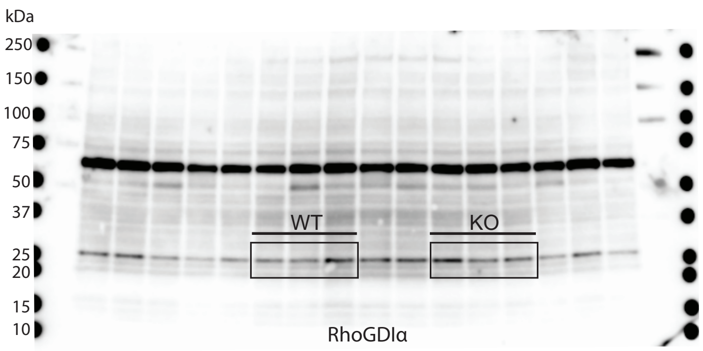

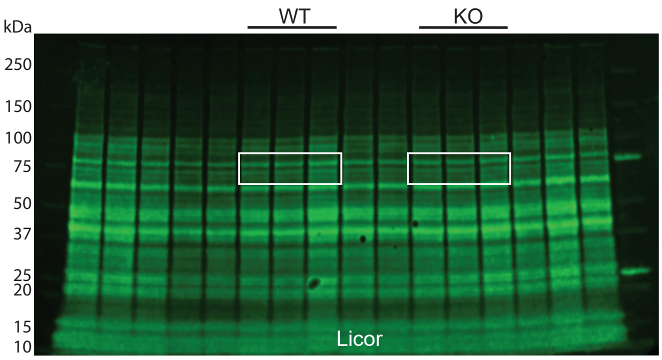

Full length blots for supplementary figure 5C

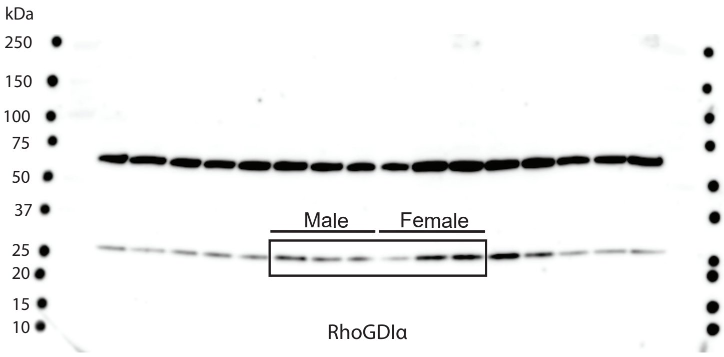

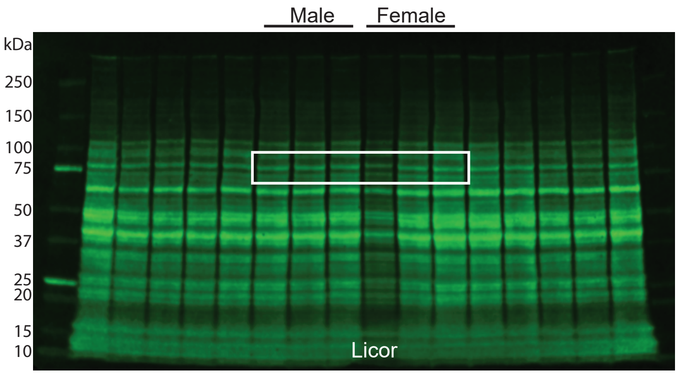

Full length blots for supplementary figure 5D

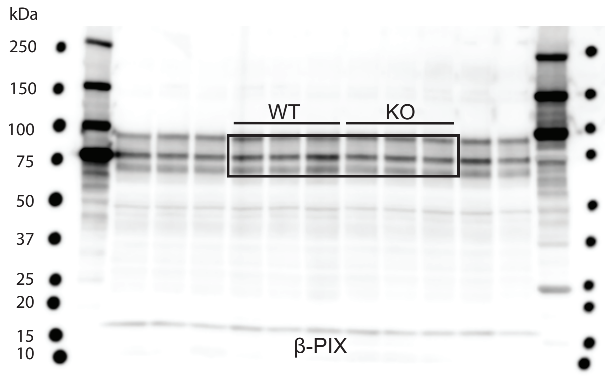

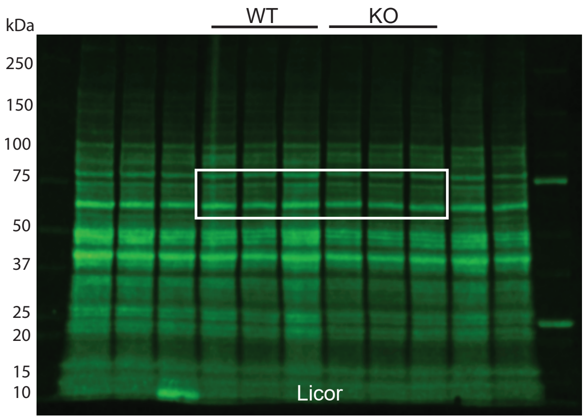

Full length blots for supplementary figure 5E

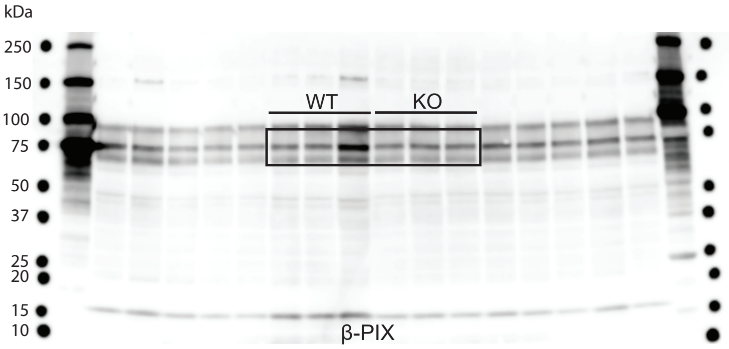

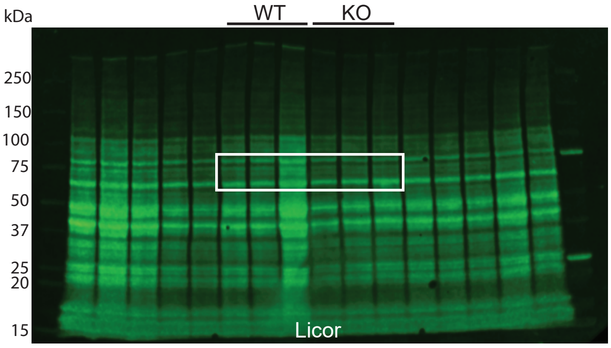

Full length blots for supplementary figure 5F

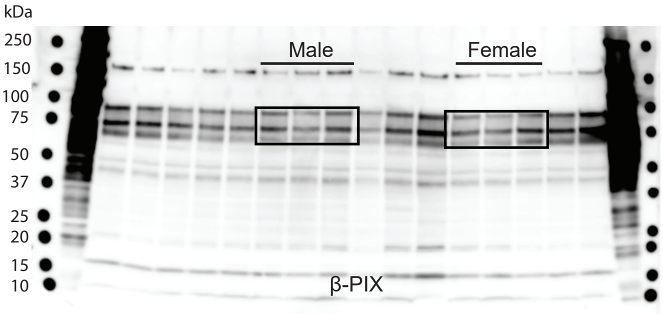

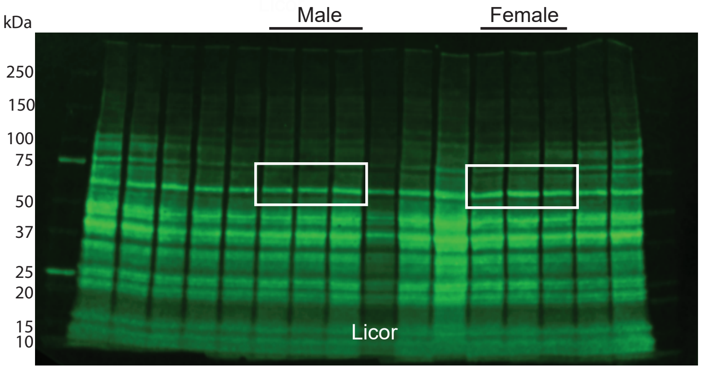

Full length blots for supplementary figure 5G

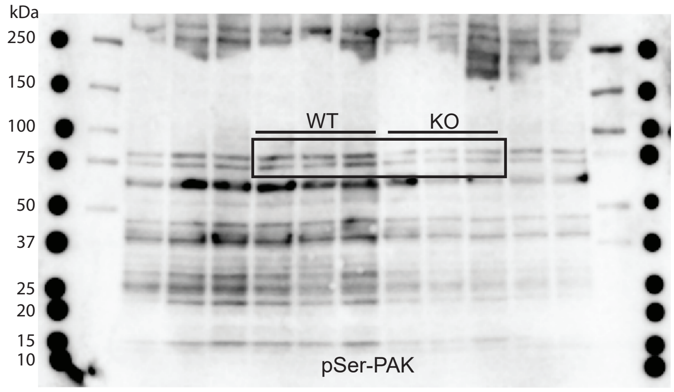

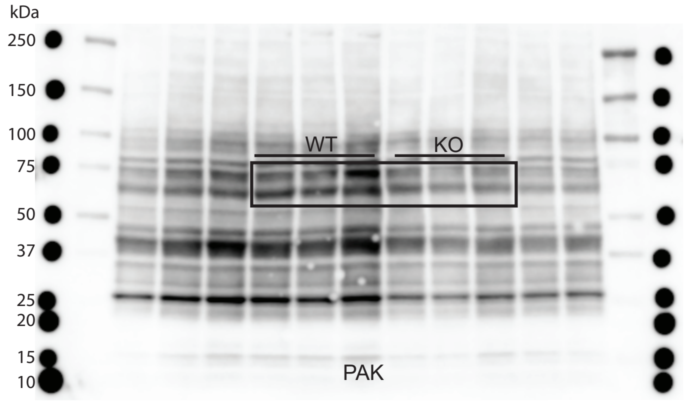

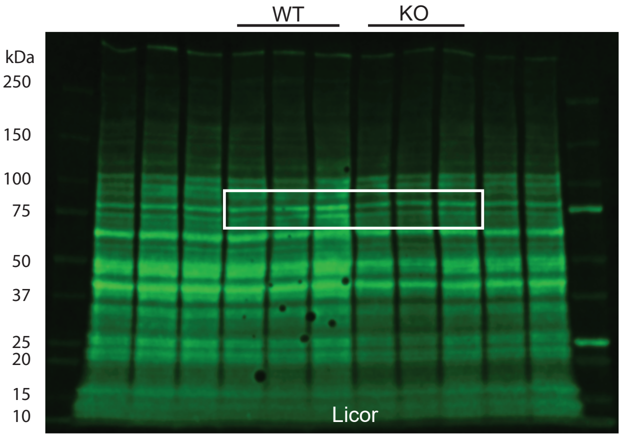

Full length blots for supplementary figure 5H

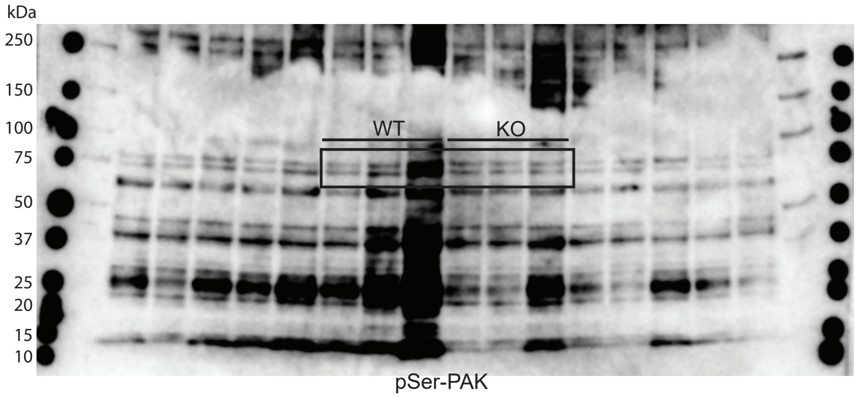

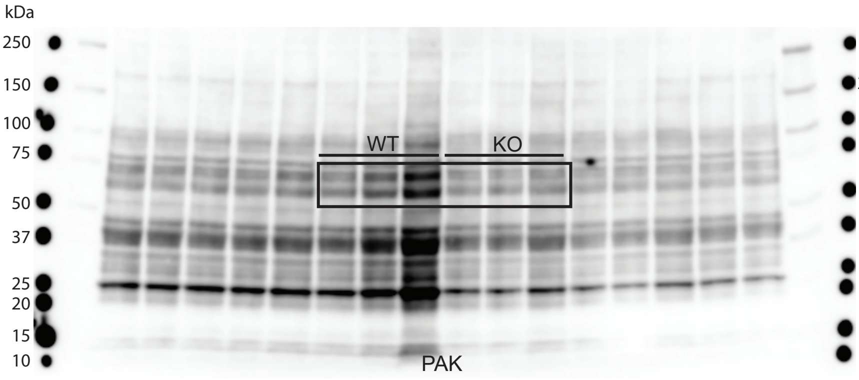

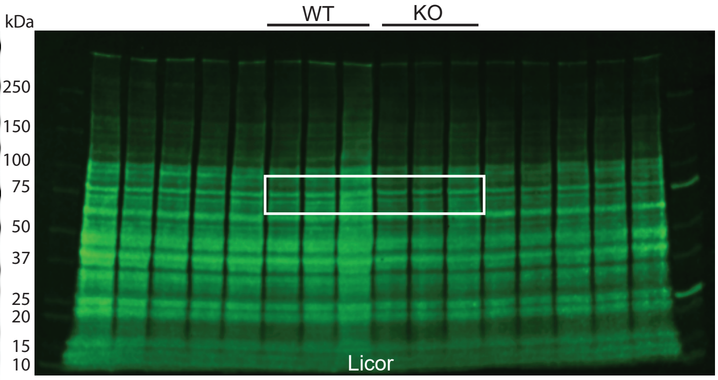

Full length blots for supplementary figure 5I

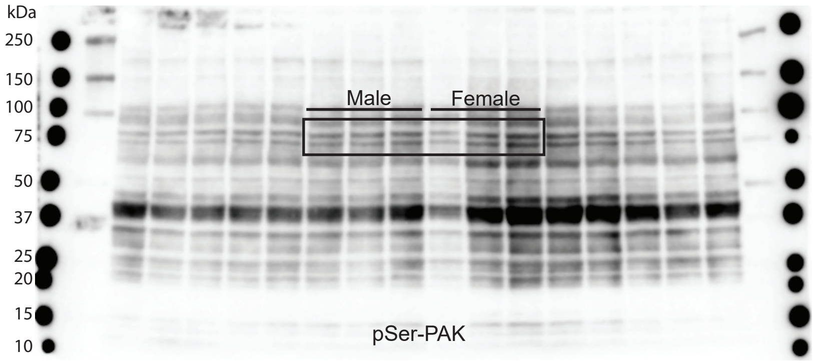

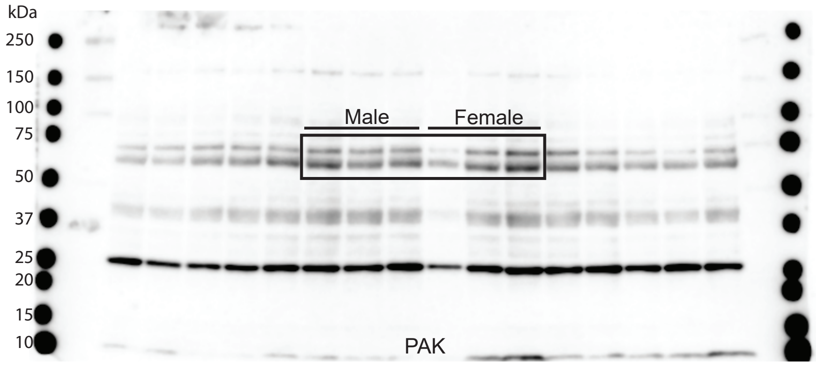

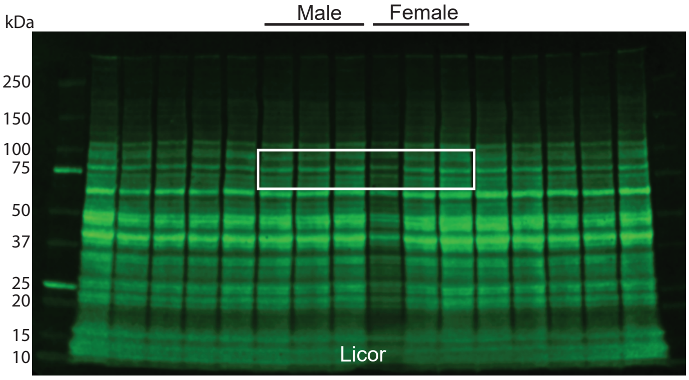

Supplement: Supplementary file 2 [file DataSheet1.pdf]
