# Supplementary material for: Syndecan-4 interacts directly with β-parvin and regulates the ILK-PINCH-β-parvin complex, the β-parvin-β-PIX-Rac1 axis, and cardiomyocyte geometry in a sex-dependent manner
Source: Front Cell Dev Biol. 2025 Aug 29;13:1569185. doi: 10.3389/fcell.2025.1569185 (PMC12447578; doi:10.3389/fcell.2025.1569185)
Supplement: Supplementary file 3 [file Image1.pdf]

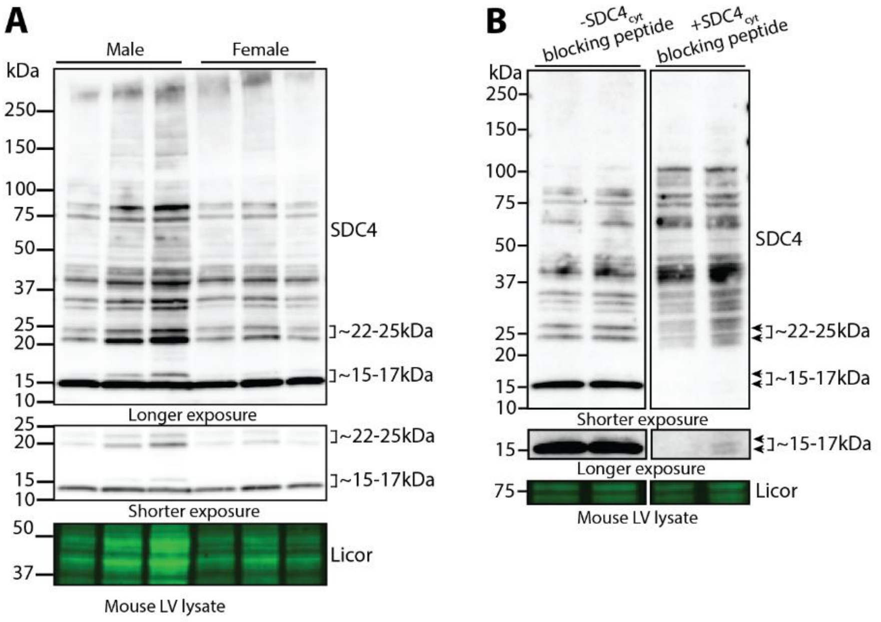

Supplementary figure 1. Syndecan-4 levels and antibody blocking experiments.

(**A**) Full-length blots of syndecan-4 immunoblotting in male and female WT LV lysate shown in Fig. 1C (n=3 hearts). Brackets on the right represent syndecan-4 positive bands detected in Suppl. Fig. 1B. (**B**) The specificity of syndecan-4 bands in the mouse LV lysate in Fig. 1C was analyzed using a blocking peptide containing the epitope of the syndecan-4 antibody. Membranes probed with the antibody and pre-incubated with the blocking peptide, are shown in the right panel, and membranes probed with the antibody only on the left. Syndecan-4 positive bands are annotated with arrows and brackets on the right. Equal loading of duplicate samples was analyzed by Licor staining (n=2 hearts).

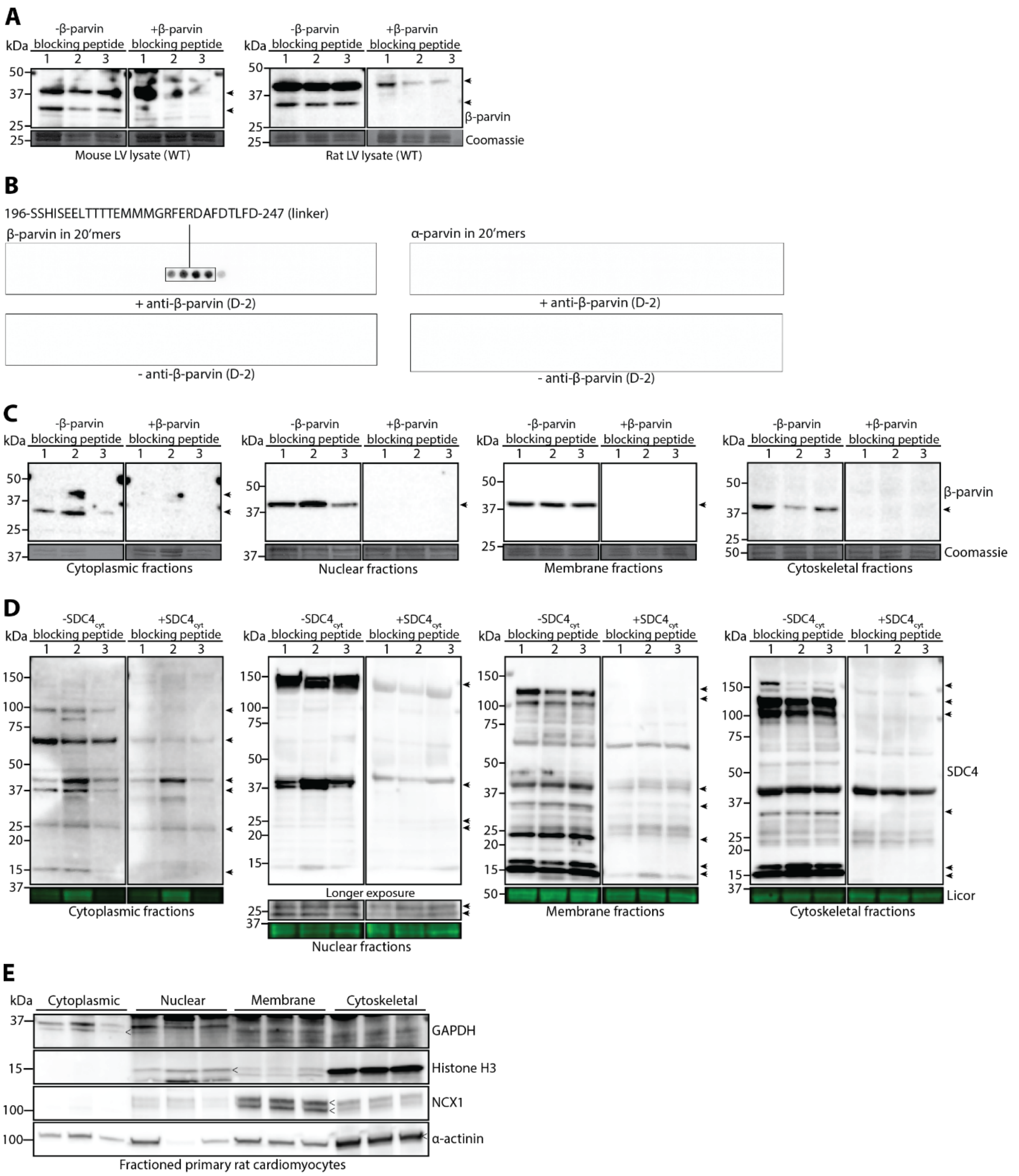

Supplementary figure 2. Antibody blocking experiments, β-parvin epitope mapping, and subcellular fraction enrichment analysis. (*continued*)

(*supplementary figure 2 continued*) (**A**) The specificity of β-parvin bands detected in the mouse and rat LV lysate in Fig. 2A-B and Fig. 5A-C was analyzed using a blocking peptide containing the epitope of the β-parvin antibody in mouse (left) and rat (right) LV lysates. Membranes probed with the antibody, pre-incubated with the blocking peptide, are shown in the right panel, and membranes probed with the antibody only on the left. β-parvin positive bands are annotated with arrows on the right. Equal loading of duplicate samples was analyzed by Coomassie staining (n=3 hearts). (**B**) The monoclonal antibody against β-parvin (D-2) was epitope mapped by overlaying the antibody onto membranes with 20`mer overlapping peptides of β-parvin or α-parvin (n=4 membranes). Peptide membranes without the primary antibody were used as a negative control. (**C-D**) The specificity of (**C**) β-parvin and (**D**) syndecan-4 bands detected in subcellular enriched fractions of isolated rat LV cardiomyocytes in Fig. 2B were analyzed using a blocking peptide containing the epitope of the β-parvin and syndecan-4 antibody, respectively. Membranes probed with the antibody, pre-incubated with the blocking peptide, are shown in the right panel of each fraction, and membranes probed with the antibody only on the left. β-parvin and syndecan-4 positive bands are annotated with arrows on the right in each panel. Equal loading of duplicate samples was analyzed by Coomassie (in C) or licor staining (in D) (n=3 hearts). (**E**) Subcellular compartment markers GAPDH (cytoplasm), histone H3 (nucleus), NCX1 (membrane) and α-actinin (cytoskeleton) were used to assess the enrichment of the fractions used in Fig. 2B (annotated with arrowheads) (n=3 hearts).

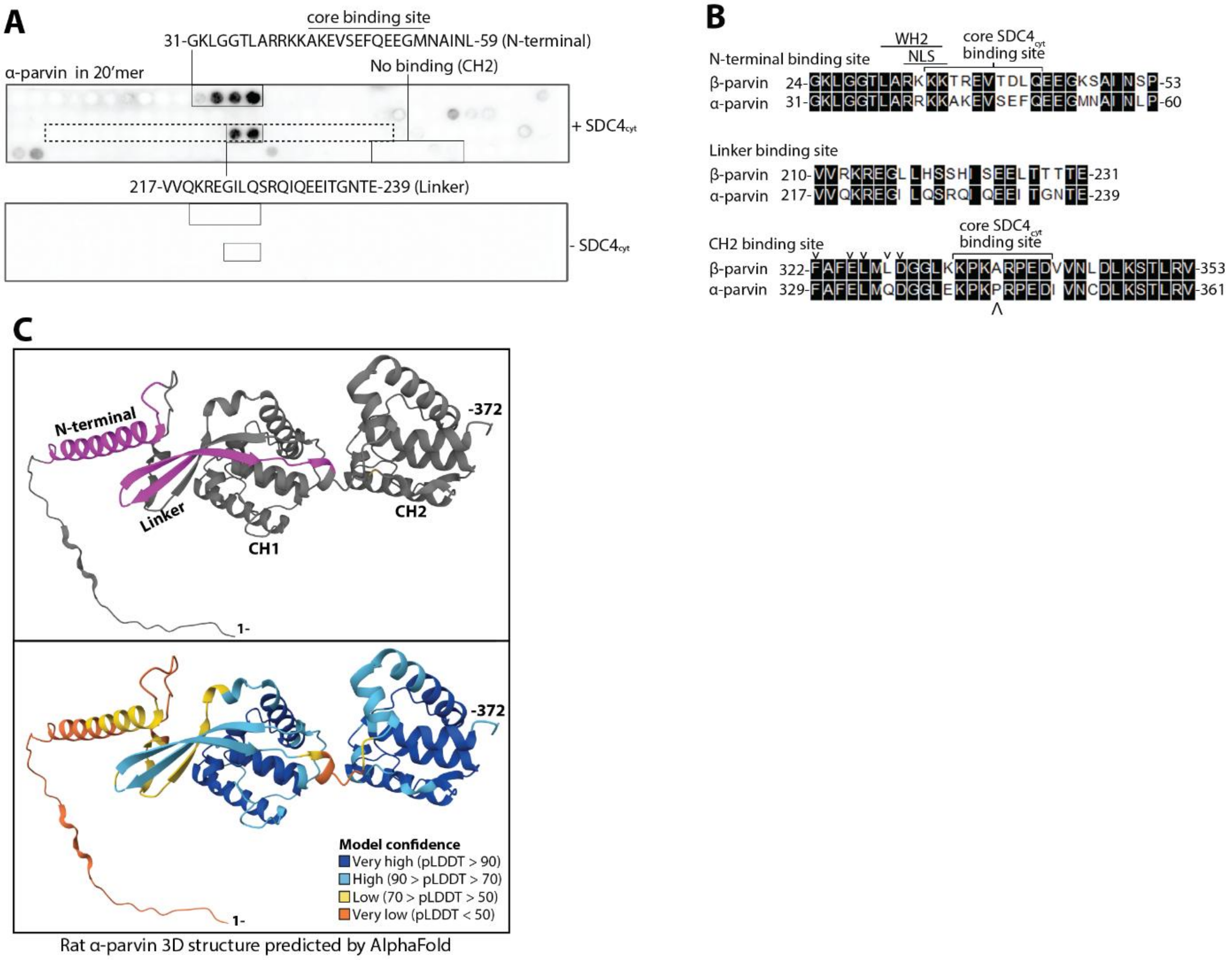

Supplementary figure 3. Mapping the syndecan-4-α-parvin interaction, alignment of the three syndecan-4 binding sites in α- and β-parvin, and AlphaFold prediction of α-parvin.

(**A**) 20`mers overlapping peptides covering FL α-parvin (rat) overlaid with biotin-ahx-SDC4$_{cyt}$ and binding detected with biotin-HRP antibodies (n=4 membranes). Peptide membranes without biotin-ahx-SDC4$_{cyt}$ incubation were used as a negative control. The peptide sequence corresponding to the linker domain in β-parvin is boxed (dotted square). Overlined sequences correspond to the syndecan-4 core binding site (common sequence of the positive peptides). (**B**) Alignment of the three syndecan-4 binding sites in β-parvin (N-terminal, linker, and CH2) with the corresponding sequences in α-parvin (DNASTAR Lasergene 17, Clustal W). A nuclear localization signal (NLS) (Olski et al., 2001) and a loosely conserved WASP-Homology 2 (WH2) (Vaynberg et al., 2018) have been identified in the N-terminal sequence (uppermost alignment). The arrowhead below the CH2 sequence (lowermost alignment) marks the proline residue which may break the α-parvin-ILK binding (Stiegler et al., 2012). (**C**) AlphaFold prediction of the protein folding structure of rat α-parvin (Varadi et al., 2021). Syndecan-4 binding sites are highlighted in magenta (top panel). The per-residue model confidence is estimated on a scale from 0-100 pLDDT, given on a color scale (lower panel).

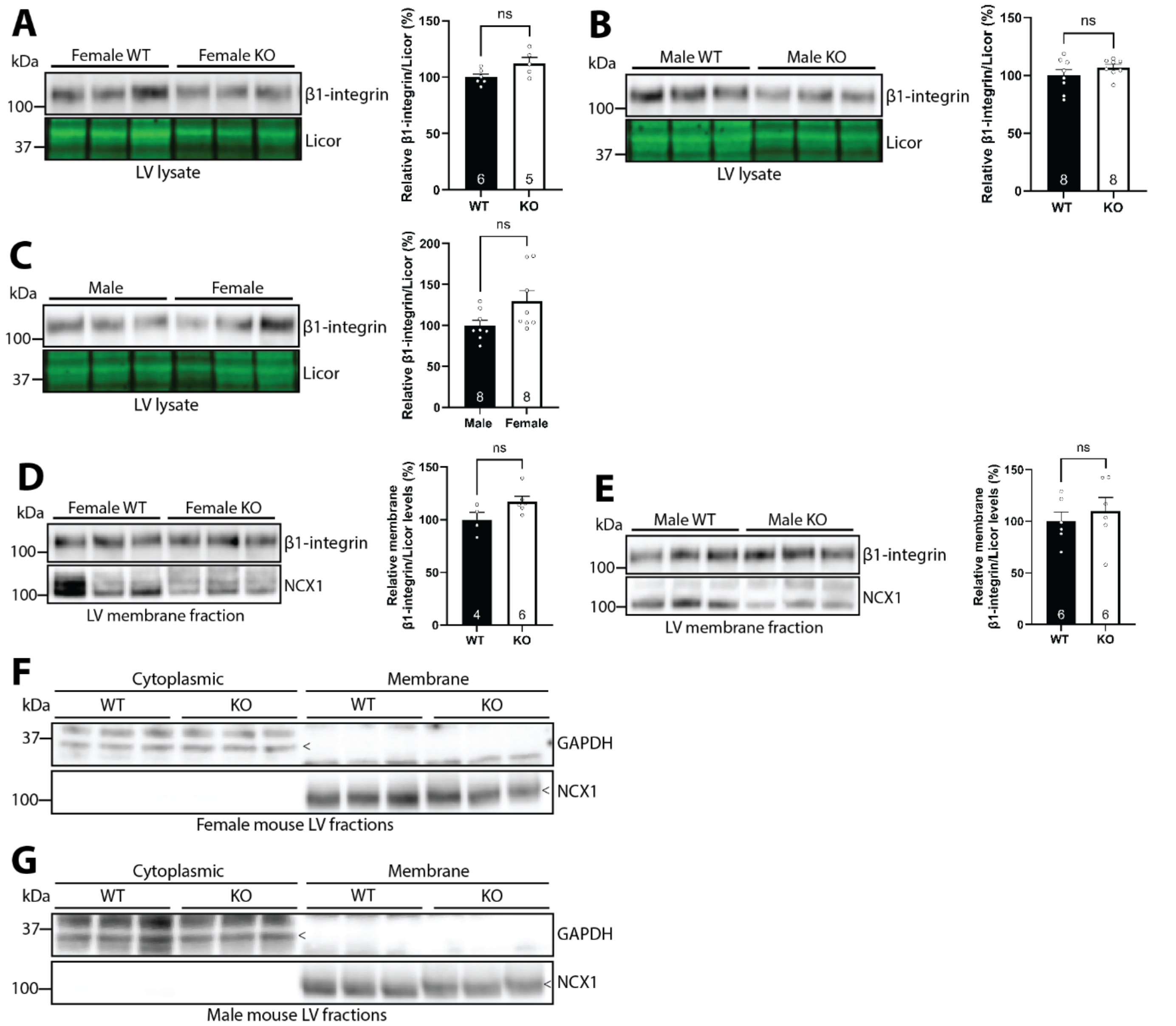

Supplementary figure 4. β1-integrin levels and subcellular fraction enrichment analysis.

β1-integrin levels in (**A**) female and (**B**) male syndecan-4 KO and WT, and (**C**) male and female WT LVs. Immunoblots of β1-integrin in (**D**) female and (**E**) male membrane-enriched fractions from syndecan-4 KO and WT hearts (n=4-6 hearts). All values are presented as mean percentages ± SEM, normalized to licor or subcellular marker levels, before being related to WT or male levels (set to 100%). Differences between the groups were analyzed using unpaired two-tailed *t*-tests (A, B, D, and E) or Mann-Whitney *U*-tests (C) due to normal or non-normal distribution analyzed by Shapiro-Wilk testing (ns: not significant). Subcellular compartment markers GAPDH (cytoplasm) and NCX1 (membrane) were used to assess the enrichment of (**F**) female and (**G**) male syndecan-4 WT and KO fractions used in Fig. 5D-E, 6D-E and I-J, 7G-H, and 8A-F (n=3 hearts).

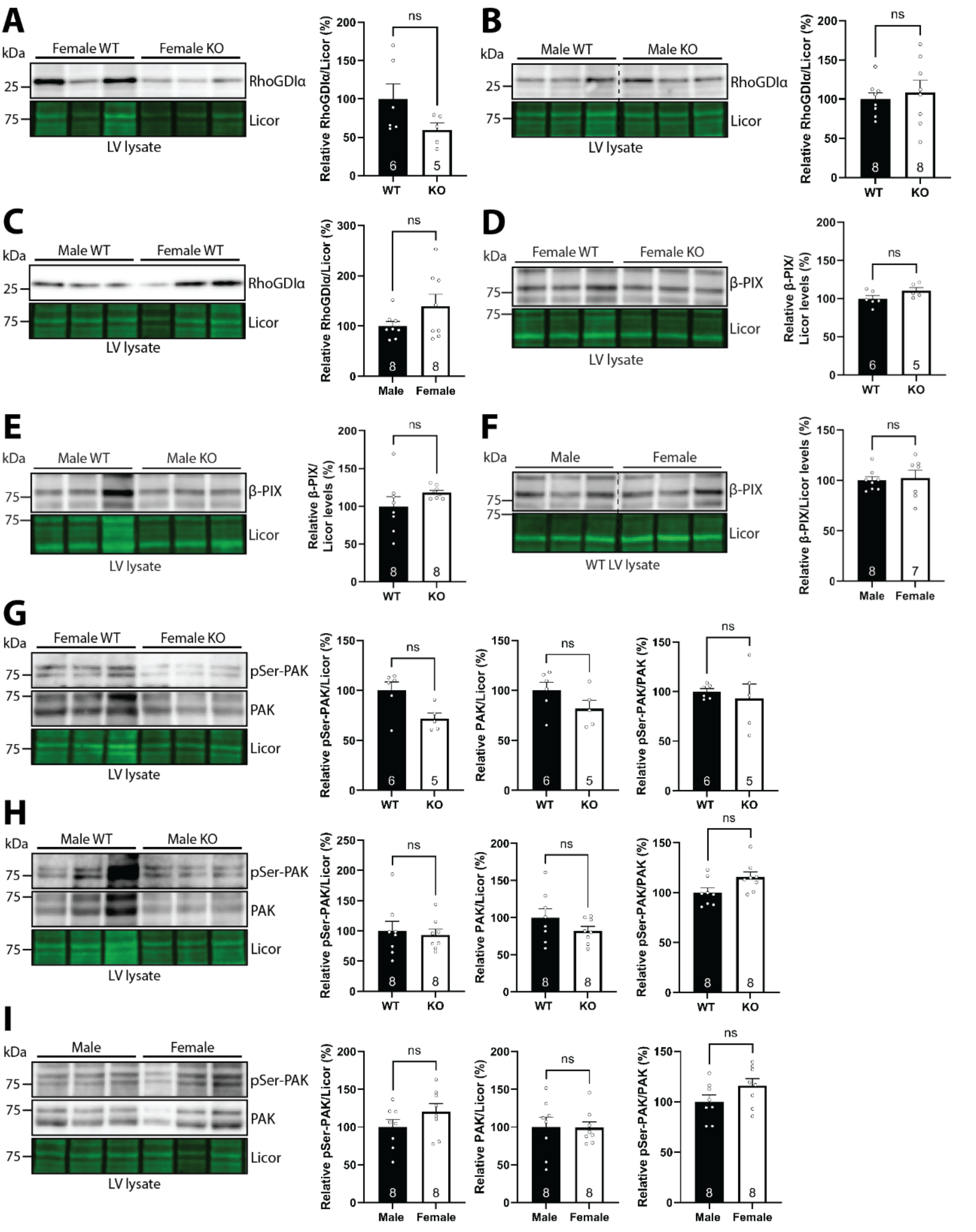

Supplementary figure 5. Levels of RhoGDIα, β-PIX, and pSer-PAK in male vs. female WT LVs or cytoplasmic-enriched fractions. (*continued*)

(*supplementary figure 5 continued*) Immunoblot of RhoGDIα in (**A**) female and (**B**) male syndecan-4 KO and WT, and (**C**) male and female WT LV lysates (n=5-8). Immunoblot of β-PIX in (**D**) female and (**E**) male syndecan-4 KO and WT, and (**F**) WT LV lysates (n=5-8 hearts). Immunoblot of pSer-PAK and PAK in LV lysates from (**G**) female and **(H)** male syndecan-4 KO and WT, and (**I**) male and female WT mice (n=5-8 hearts)**.** All values are presented as mean percentages ± SEM. Immunoblots were normalized to licor, before being related to levels in LVs of female or male WT LVs (set to 100%). Differences between the groups were analyzed using unpaired two-tailed *t*-tests (A, B, C, D, E, F, G, H, and I) or Mann-Whitney *U*-tests (G) due to normal or non-normal distribution analyzed by Shapiro-Wilk testing (ns: not significant).
